# Supplementary material for: Progression of chronic pain and associated health-related quality of life and healthcare resource use over 5 years after total knee replacement: evidence from a cohort study
Source: BMJ Open. 2022 Apr 25;12(4):e058044. doi: 10.1136/bmjopen-2021-058044 (PMC9039409; doi:10.1136/bmjopen-2021-058044)
Supplement: Supplementary data [file bmjopen-2021-058044supp001.pdf]

ONLINE SUPPLEMENTAL MATERIAL

Tables

Table A.1: Observed and imputed health utility and Oxford Knee Score pain subscale (OKS-PS) scores per year

Observed						Imputed							
year	n	mean	SD	min	max	year	n	Pooled mean	Pooled SE	95% confidence interval		min	max
Health utility (EQ5D-3L) index													
pre-op	491	0.447	0.291	-0.239	1	pre-op	550	0.462	0.112	0.243	0.682	-0.349	1
1	531	0.742	0.253	-0.319	1	1	550	0.738	0.104	0.534	0.943	-0.319	1
2	447	0.765	0.265	-0.429	1	2	550	0.754	0.106	0.546	0.962	-0.429	1
3	403	0.761	0.253	-0.358	1	3	550	0.748	0.103	0.547	0.950	-0.358	1
4	344	0.759	0.263	-0.135	1	4	550	0.744	0.103	0.542	0.947	-0.239	1
5	299	0.751	0.272	-0.239	1	5	550	0.740	0.104	0.536	0.943	-0.239	1
OKS pain subscale score													
pre-op	488	10.09	4.59	0	26	pre-op	550	10.85	0.48	9.90	11.79	0	28
1	525	22.06	6.14	1	28	1	550	22.09	0.51	21.09	23.09	1	28
2	447	23.09	5.71	0	28	2	550	22.66	0.51	21.67	23.66	0	28
3	405	23.37	5.36	3	28	3	550	22.91	0.50	21.94	23.89	1	28
4	350	23.61	5.37	3	28	4	550	23.38	0.49	22.41	24.35	1	28
5	298	23.45	5.52	6	28	5	550	23.43	0.50	22.45	24.40	2	28

Pre-op: pre-operative; SD: standard deviation; SE: standard error

Table A.2: Year 5 study participant demographics

Variable	Observed OKS-PS at Year 5 (n=286)			Missing OKS-PS at Year 5 (n=264)		
**Age at total knee replacement (n, mean, mode)**						
	286	70	73	264	70	73
**Gender (n, %)**						
Female	159	55.6		155	58.7	
Male	127	44.4		109	41.3	
**IMD 2010 score (n, mean, mode)**						
	285	10.9	7.17	264	14.8	15.71
Missing (n (%))	1	(0.35)		0	(0.00)	
**BMI (n, mean, mode)**						
	284	30.3	26.79	262	31.0	30.9
Missing (n (%))	2	(0.70)		2	(0.76)	
**OKS at pre-operation (n, mean, (SD))**						
	251	20.1	(7.17)	233	18.4	(7.78)
Missing (n (%))	35	(12.24)		31	(11.74)	
**EQ5D at pre-operation (n, mean, (SD))**						
	252	0.4736	(0.276)	239	0.4180	(0.304)
Missing (n (%))	34	(11.89)		25	(9.47)	

Table A.3: Number of missing items during the 5 years of follow-up

	Total		Non-Chronic Pain		Chronic Pain		No OKS Category	
	n	%	n	%	n	%	n	%
**Number of years of missing OKS items**								
0	175	31.8	163	35.6	12	17.9	0	0
1	141	25.6	120	26.2	18	26.9	3	12
2	113	20.5	94	20.5	9	13.4	10	40
3	57	10.4	42	9.2	12	17.9	3	12
4	56	10.2	37	8.1	15	22.4	4	16
5	8	1.5	2	0.4	1	1.5	5	20
**Number of years of missing EQ5D items**								
0	219	39.8	201	43.9	14	20.9	4	16
1	116	21.1	95	20.7	14	20.9	7	28
2	100	18.2	81	17.7	14	20.9	5	20
3	56	10.2	41	9	13	19.4	2	8
4	53	9.6	37	8.1	11	16.4	5	20
5	6	1.1	3	0.7	1	1.5	2	8

Cole S, *et al. BMJ Open* 2022; 12:e058044. doi: 10.1136/bmjopen-2021-058044

Table A.4: Logistic regression p-values: preoperative variables associated with missingness - significant at a 5% level

	Missing OKS-PS				
	**Year 1**	**Year 2**	**Year 3**	**Year 4**	**Year 5**
**Age at operation**	0.227	0.790	0.906	0.483	0.537
**Gender**	0.058*	0.172	0.410	0.079*	0.475
**EQ5D dimension 1**	0.986	0.241	0.395	0.670	0.348
**EQ5D dimension 2**	**0.021**	**0.015**	**0.007**	**0.009**	**0.020**
**EQ5D dimension 3**	0.205	**0.010**	**0.037**	0.088*	0.211
**EQ5D dimension 4**	**0.025**	**0.008**	**0.025**	**0.002**	0.116
**EQ5D dimension 5**	0.474	**0.030**	0.133	0.404	0.101
**OKS Q1**	0.939	**0.026**	**0.024**	**0.016**	0.165
**OKS Q4**	0.052*	**0.000**	**0.003**	**0.000**	**0.003**
**OKS Q5**	0.361	0.138	0.173	**0.025**	0.633
**OKS Q6**	**0.039**	**0.016**	**0.021**	0.056*	0.997
**OKS Q8**	0.839	0.080*	0.424	**0.014**	0.641
**OKS Q9**	0.184	**0.004**	0.140	**0.001**	0.105
**OKS Q10**	0.086*	**0.000**	**0.000**	**0.001**	**0.006**

*significant at a 10% level only

Table A.5: Health utility scores per year by chronic pain group

	Pooled mean	Pooled SE	95% confidence interval	
**Chronic pain 1-year after total knee replacement**				
Pre-op	0.307	0.197	-0.079	0.694
Year 1	0.399	0.194	0.019	0.778
Year 2	0.527	0.193	0.148	0.906
Year 3	0.523	0.194	0.143	0.904
Year 4	0.610	0.186	0.245	0.974
Year 5	0.659	0.183	0.300	1.018
**Non-chronic pain 1-year after total knee replacement**				
Pre-op	0.485	0.114	0.262	0.708
Year 1	0.787	0.097	0.597	0.976
Year 2	0.786	0.105	0.581	0.991
Year 3	0.781	0.100	0.584	0.977
Year 4	0.764	0.104	0.559	0.968
Year 5	0.751	0.106	0.543	0.960

Pre-op: pre-operative; SE: standard error

Table A.6: Oxford Knee Score pain subscale (OKS-PS) scores per year by chronic pain group

	Pooled mean	Pooled SE	Confidence interval	
**Chronic pain 1-year after total knee replacement**				
Pre-op	8.04	0.79	6.50	9.59
Year 1	9.73	0.66	8.45	11.02
Year 2	16.06	0.98	14.15	17.98
Year 3	17.05	1.00	15.09	19.00
Year 4	19.17	1.01	17.19	21.15
Year 5	20.80	0.98	18.88	22.71
**Non-chronic pain 1-year after total knee replacement**				
Pre-op	11.25	0.50	10.27	12.22
Year 1	23.86	0.42	23.03	24.69
Year 2	23.61	0.49	22.65	24.56
Year 3	23.76	0.48	22.81	24.70
Year 4	23.98	0.48	23.03	24.93
Year 5	23.80	0.50	22.82	24.79

Pre-op: pre-operative; SE: standard error

Table A.7a: Primary care healthcare resource use by chronic pain (CP) group, defined 1-year after total knee replacement (Pre-op, Year 1 and Year 2)

	Pre-op		Year 1		Year 2	
	**CP (n=70)**	**Non-CP (n=482)**	**CP (n=70)**	**Non-CP (n=482)**	**CP (n=70)**	**Non-CP (n=482)**
**Saw a general practitioner (n (%))**						
Yes	58 (82.86)	390 (80.91)	44 (62.86)	135 (28.01)	24 (34.29)	46 (9.54)
No	8 (11.43)	43 (8.92)	23 (32.86)	335 (69.5)	22 (31.43)	361 (74.9)
Missing	*4 (5.71)*	*49 (10.17)*	*3 (4.29)*	*12 (2.49)*	*24 (34.29)*	*75 (15.56)*
Number of visits – last 12 months (mean (total))						
NHS	3.8 (209)	3.43 (1323)	2.66 (154)	0.64 (288)	1.29 (54)	0.21 (81)
Private	0 (0)	0.08 (4)	0.25 (6)	0.02 (6)	0 (0)	0 (0)
**Saw a physiotherapist**						
Yes	25 (35.71)	153 (31.74)	42 (60)	215 (44.61)	15 (21.43)	71 (14.73)
No	40 (57.14)	251 (52.07)	25 (35.71)	253 (52.49)	29 (41.43)	330 (68.46)
Missing	*5 (7.14)*	*78 (16.18)*	*3 (4.29)*	*14 (2.9)*	*26 (37.14)*	*81 (16.8)*
Number of visits – last 12 months (mean (total))						
NHS	1.35 (81)	1.12 (422)	4.08 (241)	2.28 (973)	1.65 (66)	0.81 (314)
Private	0.02 (1)	0.34 (92)	2.97 (86)	0.6 (167)	0 (0)	0.12 (39)
**Saw a nurse/practitioner**						
Yes	14 (20)	102 (21.16)	18 (25.71)	84 (17.43)	4 (5.71)	16 (3.32)
No	48 (68.57)	292 (60.58)	41 (58.57)	369 (76.56)	36 (51.43)	373 (77.39)
Missing	*8 (11.43)*	*88 (18.26)*	*11 (15.71)*	*29 (6.02)*	*30 (42.86)*	*93 (19.29)*
Number of visits – last 12 months (mean (total))						
NHS	0.7 (42)	0.72 (271)	2.34 (131)	0.47 (204)	0.43 (17)	0.08 (31)
Private	0 (0)	0 (0)	0 (0)	0 (0)	0 (0)	0 (1)
**Saw an alternative practitioner**						
Yes	3 (4.29)	41 (8.51)	7 (10)	19 (3.94)	4 (5.71)	5 (1.04)
No	61 (87.14)	386 (80.08)	62 (88.57)	456 (94.61)	42 (60)	406 (84.23)
Missing	*6 (8.57)*	*55 (11.41)*	*1 (1.43)*	*7 (1.45)*	*24 (34.29)*	*71 (14.73)*
Number of visits – last 12 months (mean (total))						
NHS	0.03 (2)	0.12 (46)	0 (0)	0.01 (3)	0.12 (5)	0 (0)
Private	0.13 (8)	0.32 (134)	0.32 (21)	0.11 (51)	0.2 (9)	0.11 (46)

Cole S, *et al. BMJ Open* 2022; 12:e058044. doi: 10.1136/bmjopen-2021-058044

Table A.7b: Primary care healthcare resource use by chronic pain (CP) group, defined 1-year after total knee replacement (Years 3-5)

	Year 3		Year 4		Year 5	
	**CP (n=70)**	**Non-CP (n=482)**	**CP (n=70)**	**Non-CP (n=482)**	**CP (n=70)**	**Non-CP (n=482)**
**Saw a general practitioner (n (%))**						
Yes	13 (18.57)	28 (5.81)	7 (10)	17 (3.53)	6 (8.57)	23 (4.77)
No	29 (41.43)	355 (73.65)	24 (34.29)	312 (64.73)	16 (22.86)	271 (56.22)
Missing	*28 (40)*	*99 (20.54)*	*39 (55.71)*	*153 (31.74)*	*48 (68.57)*	*188 (39)*
Number of visits – last 12 months (mean (total))						
NHS	0.69 (27)	0.16 (61)	0.63 (19)	0.11 (36)	0.33 (6)	0.13 (36)
Private	0 (0)	0 (0)	0 (0)	0 (0)	0.06 (1)	0 (0)
**Saw a physiotherapist**						
Yes	7 (10)	20 (4.15)	3 (4.29)	10 (2.07)	2 (2.86)	11 (2.28)
No	34 (48.57)	353 (73.24)	25 (35.71)	307 (63.69)	17 (24.29)	275 (57.05)
Missing	*29 (41.43)*	*109 (22.61)*	*42 (60)*	*165 (34.23)*	*51 (72.86)*	*196 (40.66)*
Number of visits – last 12 months (mean (total))						
NHS	29 (41.43)	355 (73.65)	24 (34.29)	312 (64.73)	16 (22.86)	271 (56.22)
Private	*28 (40)*	*99 (20.54)*	*39 (55.71)*	*153 (31.74)*	*48 (68.57)*	*188 (39)*
**Saw a nurse/practitioner**						
Yes	6 (8.57)	3 (0.62)	1 (1.43)	3 (0.62)	0 (0)	1 (0.21)
No	35 (50)	365 (75.73)	26 (37.14)	314 (65.15)	19 (27.14)	283 (58.71)
Missing	*29 (41.43)*	*114 (23.65)*	*43 (61.43)*	*165 (34.23)*	*51 (72.86)*	*198 (41.08)*
Number of visits – last 12 months (mean (total))						
NHS	0.16 (6)	0.02 (7)	0.15 (4)	0.02 (7)	0 (0)	0 (0)
Private	0 (0)	0 (0)	0 (0)	0 (0)	0 (0)	0 (0)
**Saw an alternative practitioner**						
Yes	6 (8.57)	6 (1.24)	2 (2.86)	7 (1.45)	1 (1.43)	3 (0.62)
No	37 (52.86)	374 (77.59)	28 (40)	322 (66.8)	20 (28.57)	290 (60.17)
Missing	*27 (38.57)*	*102 (21.16)*	*40 (57.14)*	*153 (31.74)*	*49 (70)*	*189 (39.21)*
Number of visits – last 12 months (mean (total))						
NHS	0.03 (1)	0.01 (5)	0 (0)	0.02 (6)	0 (0)	0 (0)
Private	0.48 (19)	0.11 (42)	0.07 (2)	0.05 (15)	0 (0)	0 (0)

Cole S, *et al. BMJ Open* 2022; 12:e058044. doi: 10.1136/bmjopen-2021-058044

Table A.8a: Hospital care resource use by chronic pain (CP) group, defined 1-year after total knee replacement (Pre-op, Year 1 and Year 2)

	Pre-op		Year 1		Year 2	
	**CP (n=70)**	**Non-CP (n=482)**	**CP (n=70)**	**Non-CP (n=482)**	**CP (n=70)**	**Non-CP (n=482)**
**Saw a hospital doctor**						
Yes	53 (75.71)	354 (73.44)	36 (51.43)	106 (21.99)	23 (32.86)	43 (8.92)
No	12 (17.14)	65 (13.49)	27 (38.57)	355 (73.65)	22 (31.43)	357 (74.07)
Missing	*5 (7.14)*	*63 (13.07)*	*7 (10)*	*21 (4.36)*	*25 (35.71)*	*82 (17.01)*
Number of visits – last 12 months (mean (total))						
NHS	2.52 (151)	1.8 (687)	1.72 (103)	0.4 (177)	1.3 (56)	0.22 (88)
Private	0.08 (1)	0.17 (13)	0 (0)	0 (1)	0 (0)	0 (0)
**Visited an accident and emergency department**						
Yes	9 (12.86)	51 (10.58)	6 (8.57)	15 (3.11)	1 (1.43)	5 (1.04)
No	55 (78.57)	373 (77.39)	62 (88.57)	462 (95.85)	44 (62.86)	403 (83.61)
Missing	*6 (8.57)*	*58 (12.03)*	*2 (2.86)*	*5 (1.04)*	*25 (35.71)*	*74 (15.35)*
Number of visits – last 12 months (mean (total))						
NHS	0.21 (13)	0.17 (72)	0.24 (16)	0.03 (12)	0.02 (1)	0.02 (7)
**Re-admitted to the (site-specific) hospital**						
Yes			15 (21.43)	29 (6.02)	6 (8.57)	8 (1.66)
No			54 (77.14)	447 (92.74)	40 (57.14)	409 (84.85)
Missing			*1 (1.43)*	*6 (1.24)*	*24 (34.29)*	*65 (13.49)*
Number of visits – last 12 months (mean (total))						
Days			1.07 (73)	0.25 (117)	0.59 (27)	0.04 (18)
**Admitted to any other hospital**						
Yes			1 (1.43)	6 (1.24)	2 (2.86)	2 (0.41)
No			67 (95.71)	470 (97.51)	45 (64.29)	412 (85.48)
Missing			*2 (2.86)*	*6 (1.24)*	*23 (32.86)*	*68 (14.11)*
Number of visits – last 12 months (mean (total))						
Days			0.1 (7)	0.04 (17)	0.43 (20)	0.01 (5)

Cole S, *et al. BMJ Open* 2022; 12:e058044. doi: 10.1136/bmjopen-2021-058044

Table A.8b: Hospital care resource use by chronic pain (CP) group, defined 1-year after total knee replacement (Years 3-5)

	Year 3		Year 4		Year 5	
	**CP (n=70)**	**Non-CP (n=482)**	**CP (n=70)**	**Non-CP (n=482)**	**CP (n=70)**	**Non-CP (n=482)**
**Saw a hospital doctor**						
Yes	14 (20)	19 (3.94)	7 (10)	10 (2.07)	6 (8.57)	16 (3.32)
No	31 (44.29)	355 (73.65)	24 (34.29)	311 (64.52)	16 (22.86)	274 (56.85)
Missing	*25 (35.71)*	*108 (22.41)*	*39 (55.71)*	*161 (33.4)*	*48 (68.57)*	*192 (39.83)*
Number of visits – last 12 months (mean (total))						
NHS	0.7 (30)	0.11 (41)	0.45 (13)	0.06 (20)	0.29 (6)	0.08 (23)
Private	0 (0)	0 (0)	0 (0)	0 (0)	0 (0)	0 (0)
**Visited an accident and emergency department**						
Yes	2 (2.86)	5 (1.04)	0 (0)	3 (0.62)	0 (0)	3 (0.62)
No	41 (58.57)	375 (77.8)	31 (44.29)	323 (67.01)	21 (30)	288 (59.75)
Missing	*27 (38.57)*	*102 (21.16)*	*39 (55.71)*	*156 (32.37)*	*49 (70)*	*191 (39.63)*
Number of visits – last 12 months (mean (total))						
NHS	0.09 (4)	0.01 (2)	0 (0)	0 (1)	0 (0)	0 (0)
**Re-admitted to the (site-specific) hospital**						
Yes	3 (4.29)	6 (1.24)	1 (1.43)	3 (0.62)	0 (0)	4 (0.83)
No	40 (57.14)	383 (79.46)	32 (45.71)	331 (68.67)	24 (34.29)	290 (60.17)
Missing	*27 (38.57)*	*93 (19.29)*	*37 (52.86)*	*148 (30.71)*	*46 (65.71)*	*188 (39)*
Number of visits – last 12 months (mean (total))						
Days	0.14 (6)	0.12 (46)	0.06 (2)	0.09 (31)	0 (0)	0.03 (8)
**Admitted to any other hospital**						
Yes	0 (0)	1 (0.21)	0 (0)	0 (0)	0 (0)	1 (0.21)
No	42 (60)	386 (80.08)	33 (47.14)	332 (68.88)	23 (32.86)	291 (60.37)
Missing	*28 (40)*	*95 (19.71)*	*37 (52.86)*	*150 (31.12)*	*47 (67.14)*	*190 (39.42)*
Number of visits – last 12 months (mean (total))						
Days	0 (0)	0 (1)	0 (0)	0 (0)	0 (0)	0 (1)

Table A.9a: Yearly healthcare costs by chronic pain (CP) (Pre-op, Year 1 and Year 2)

	CP at year 1 (n = 70)		Non-CP at year 1 (n = 482)	
	**Mean**	**(SD)**	**Mean**	**(SD)**
**Pre-operative - Healthcare professionals**				
General practitioner	98.53	(124.17)	90.58	(104.96)
Physiotherapist	40.50	(93.39)	30.64	(73.90)
Hospital doctor	258.86	(559.74)	171.04	(225.45)
Nurse	9.30	(27.60)	8.71	(29.74)
Alternative practitioner	1.00	(8.37)	3.34	(50.00)
**Pre-op - Hospital visits**				
Accident and emergency	21.60	(56.64)	17.78	(51.73)
Re-admitted to hospital				
Admitted to another hospital				
**Pre-op - Total**	**429.79**	**(611.41)**	**322.09**	**(337.22)**
**Year 1 - Healthcare professionals**				
General practitioner	72.60	(133.02)	19.72	(53.62)
Physiotherapist	120.50	(179.62)	70.65	(132.22)
Hospital doctor	176.57	(300.76)	44.07	(119.12)
Nurse	29.01	(118.95)	6.56	(24.31)
Alternative practitioner	0.00	(0.00)	0.22	(2.76)
**Year 1 - Hospital visits**				
Accident and emergency	14.40	(47.37)	5.23	(29.20)
Re-admitted to hospital	1276.42	(2647.66)	303.54	(1325.34)
Admitted to another hospital	109.47	(915.92)	51.14	(489.30)
**Year 1 - Total**	**1,798.97**	**(2981.69)**	**501.12**	**(1511.82)**
**Year 2 - Healthcare professionals**				
General practitioner	25.46	(48.06)	5.55	(24.42)
Physiotherapist	33.00	(95.13)	22.80	(73.23)
Hospital doctor	96.00	(220.61)	21.91	(109.69)
Nurse	3.76	(20.00)	1.00	(7.58)
Alternative practitioner	2.50	(20.92)	0.00	(0.00)
**Year 2 - Hospital visits**				
Accident and emergency	2.40	(20.08)	1.74	(17.04)
Re-admitted to hospital	486.42	(1825.23)	86.54	(791.78)
Admitted to another hospital	158.00	(996.48)	22.95	(381.51)
**Year 2 - Total**	**807.54**	**(2233.38)**	**162.48**	**(970.07)**

Table A.9b: Yearly healthcare costs by chronic pain (CP) (Years 3-5)

	CP at year 1 (n = 70)		Non-CP at year 1 (n = 482)	
	**Mean**	**(SD)**	**Mean**	**(SD)**
**Year 3 - Healthcare professionals**				
General practitioner	12.73	(36.97)	4.18	(24.51)
Physiotherapist	9.50	(46.89)	4.72	(28.11)
Hospital doctor	51.43	(136.40)	10.21	(64.63)
Nurse	1.33	(6.32)	0.23	(4.29)
Alternative practitioner	0.50	(4.18)	0.36	(7.97)
**Year 3 - Hospital visits**				
Accident and emergency	4.80	(28.19)	1.74	(17.04)
Re-admitted to hospital	328.42	(1563.26)	77.69	(728.20)
Admitted to another hospital	0.00	(0.00)	15.90	(349.05)
**Year 3 - Total**	**408.71**	**(1630.76)**	**115.02**	**(865.95)**
**Year 4 - Healthcare professionals**				
General practitioner	8.96	(33.20)	2.46	(21.45)
Physiotherapist	11.00	(57.94)	2.03	(22.70)
Hospital doctor	22.29	(89.74)	4.98	(42.79)
Nurse	0.89	(7.41)	0.23	(3.53)
Alternative practitioner	0.00	(0.00)	0.44	(8.13)
**Year 4 - Hospital visits**				
Accident and emergency	0.00	(0.00)	1.05	(13.23)
Re-admitted to hospital	48.53	(406.00)	22.95	(381.51)
Admitted to another hospital	0.00	(0.00)	0.00	(0.00)
**Year 4 - Total**	**91.66**	**(471.78)**	**34.13**	**(431.45)**
**Year 5 - Healthcare professionals**				
General practitioner	2.83	(17.54)	2.46	(17.63)
Physiotherapist	4.50	(27.90)	1.38	(14.12)
Hospital doctor	10.29	(39.53)	5.73	(43.74)
Nurse	0.00	(0.00)	0.00	(0.00)
Alternative practitioner	0.00	(0.00)	0.00	(0.00)
**Year 5 - Hospital visits**				
Accident and emergency	0.00	(0.00)	1.05	(13.23)
Re-admitted to hospital	0.00	(0.00)	63.59	(695.91)
Admitted to another hospital	0.00	(0.00)	7.05	(154.72)
**Year 5 - Total**	**17.61**	**(65.72)**	**81.26**	**(754.21)**

Cole S, *et al. BMJ Open* 2022; 12:e058044. doi: 10.1136/bmjopen-2021-058044

Table A.10: Mean costs for fluctuating and stable groups over chronic pain (CP) status between 1 and 2 years after total knee replacement – Year 1 CP

	Year 1 CP to Year 2 CP (n=25)				Year 1 CP to Year 2 Non-CP (n=19)			
	Year 1		Year 2		Year 1		Year 2	
	mean	SD	mean	SD	mean	SD	mean	SD
**Staff costs**								
General practitioner	52.8	82.50	42.2	49.06	78.2	188.36	33.0	65.08
Physiotherapist	96.6	159.84	60.2	135.37	71.8	89.97	38.7	88.00
Hospital doctor	201.6	399.08	158.4	253.90	113.7	176.52	138.9	283.59
Nurse	47.7	186.69	3.7	12.88	23.7	72.77	9.0	35.54
Alternative practitioner	0.0	0.00	0.0	0.00	0.0	0.00	9.2	40.15
**Hospital visit costs**								
Accident and emergency	13.4	46.52	0.0	0.00	8.8	38.54	8.8	38.54
Re-admitted to hospital	884.8	2244.93	748.9	2188.64	1210.0	2870.87	806.6	2416.20
Admitted to another hospital	306.5	1532.63	442.4	1650.37	0.0	0.00	0.0	0.00
**Total costs**	1603.5	2891.87	1455.9	2710.89	1506.2	3100.76	1044.3	2785.68

Table A.11: Mean costs for fluctuating and stable groups over chronic pain (CP) status between 1 and 2 years after total knee replacement – Year 1 Non-CP

	Year 1 Non-CP to Year 2 Non-CP (n=382)				Year 1 Non-CP to Year 2 CP (n=22)			
	Year 1		Year 2		Year 1		Year 2	
	mean	SD	mean	SD	mean	SD	mean	SD
**Staff costs**								
General practitioner	20.0	57.16	5.5	23.99	43.5	54.26	24.0	52.11
Physiotherapist	66.5	124.34	24.3	75.25	101.8	164.49	63.6	120.10
Hospital doctor	41.8	116.11	20.4	100.63	125.5	223.66	125.5	280.76
Nurse	6.2	25.23	0.9	7.52	8.5	18.97	2.8	10.30
Alternative practitioner	0.3	3.09	0.0	0.00	0.0	0.00	0.0	0.00
**Hospital visit costs**								
Accident and emergency	3.5	24.09	1.8	17.12	15.3	49.43	7.6	35.82
Re-admitted to hospital	307.3	1345.38	60.2	677.32	657.1	1855.51	851.1	2320.22
Admitted to another hospital	35.6	346.23	20.1	392.08	348.3	1633.78	154.4	724.22
**Total costs**	481.2	1488.25	133.2	835.38	1300.0	2512.02	1229.0	2755.09

Figure legends

Figure A. 1 Multiple imputation boxplot based on the mean health utility score across all 50 imputations for each participant

Figure A. 2: Multiple imputation histogram based on the mean Oxford Knee Score pain subscale (OKS-PS) across all 50 imputations for each participant

Figure A.3 Number of years of OKS-PS missing items by CP status

Figure A.4 Number of years of EQ-5D missing items by CP status

Figure A. 5: Oxford Knee Score pain subscale (OKS-PS) mean scores over the 5 years after total knee replacement surgery for those in chronic pain and those not in chronic pain 1-year after surgery (based on an OKS-PS threshold score).

Figures

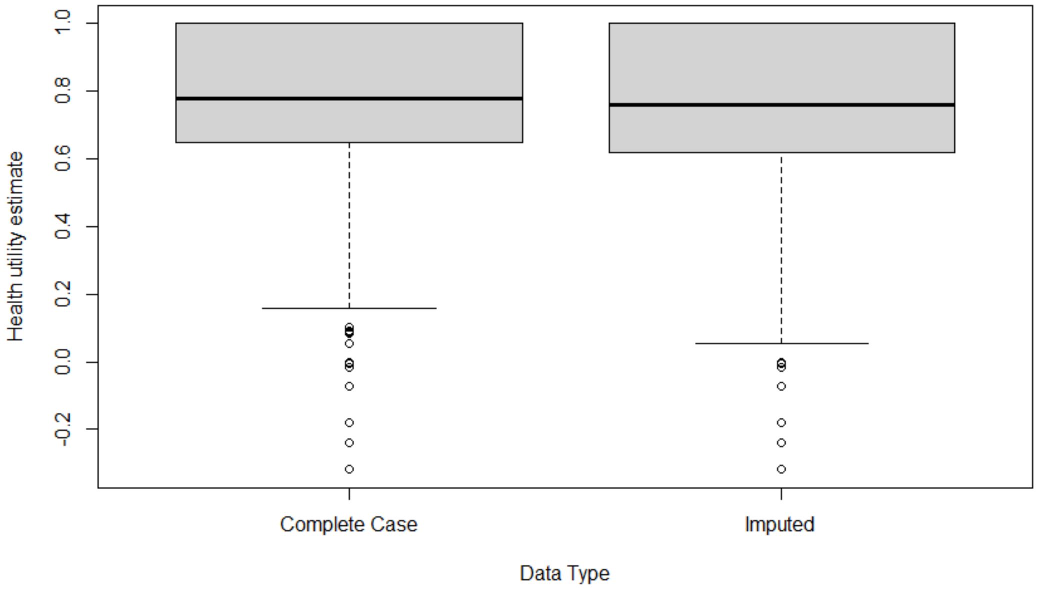
Health utility estimate
-0.2
0.0
0.2
0.4
0.6
0.8
1.0
Complete Case
Imputed
Data Type

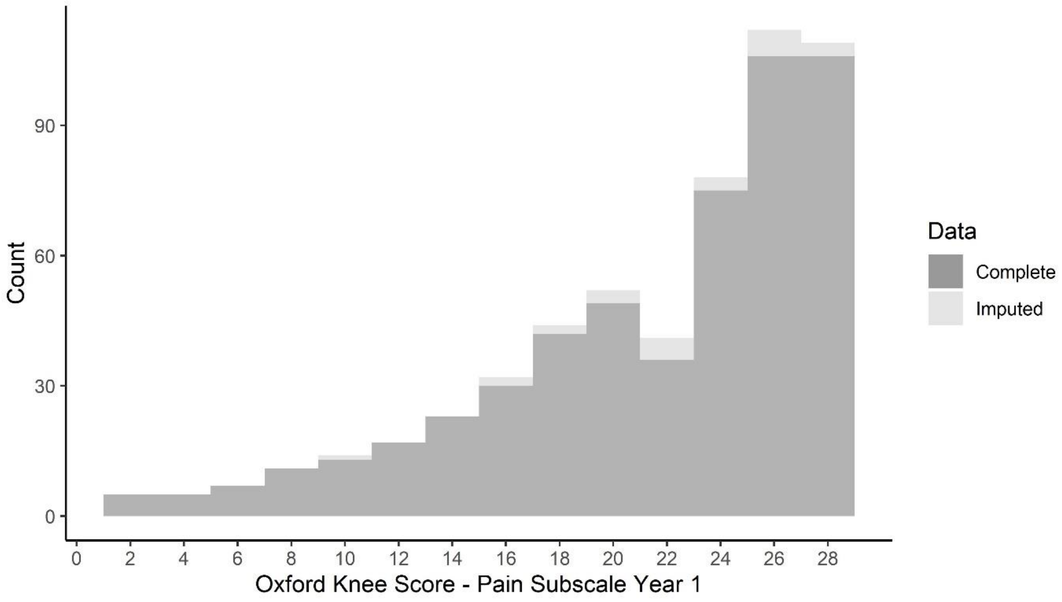
Count
90
60
30
0
0 2 4 6 8 10 12 14 16 18 20 22 24 26 28
Oxford Knee Score - Pain Subscale Year 1
Data
Complete
Imputed

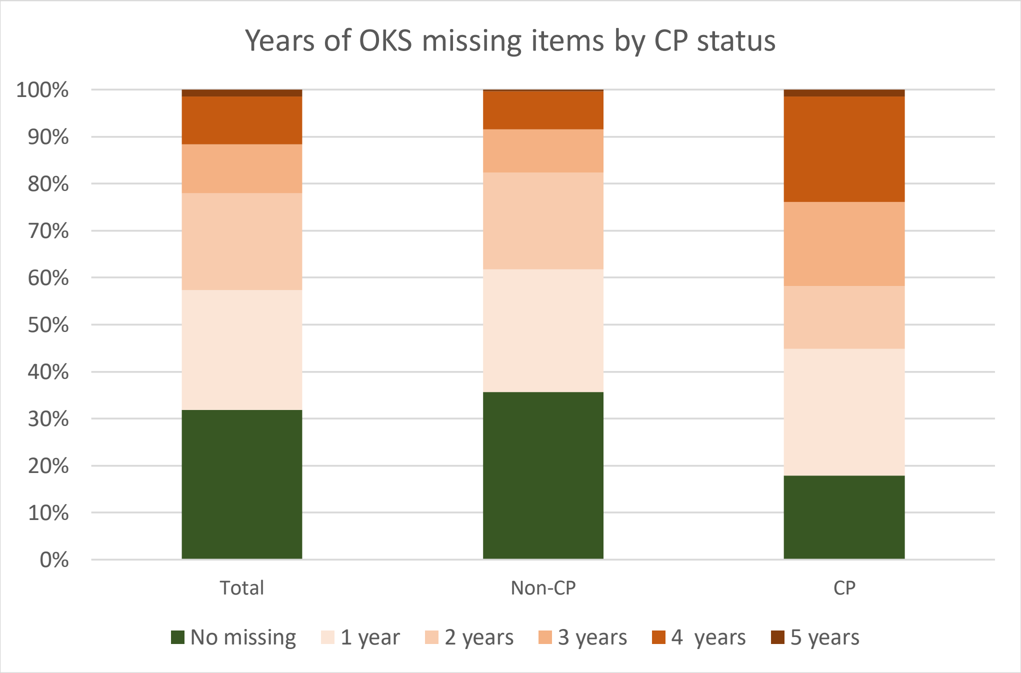
Years of OKS missing items by CP status
100%
90%
80%
70%
60%
50%
40%
30%
20%
10%
0%
Total
Non-CP
CP
No missing
1 year
2 years
3 years
4 years
5 years

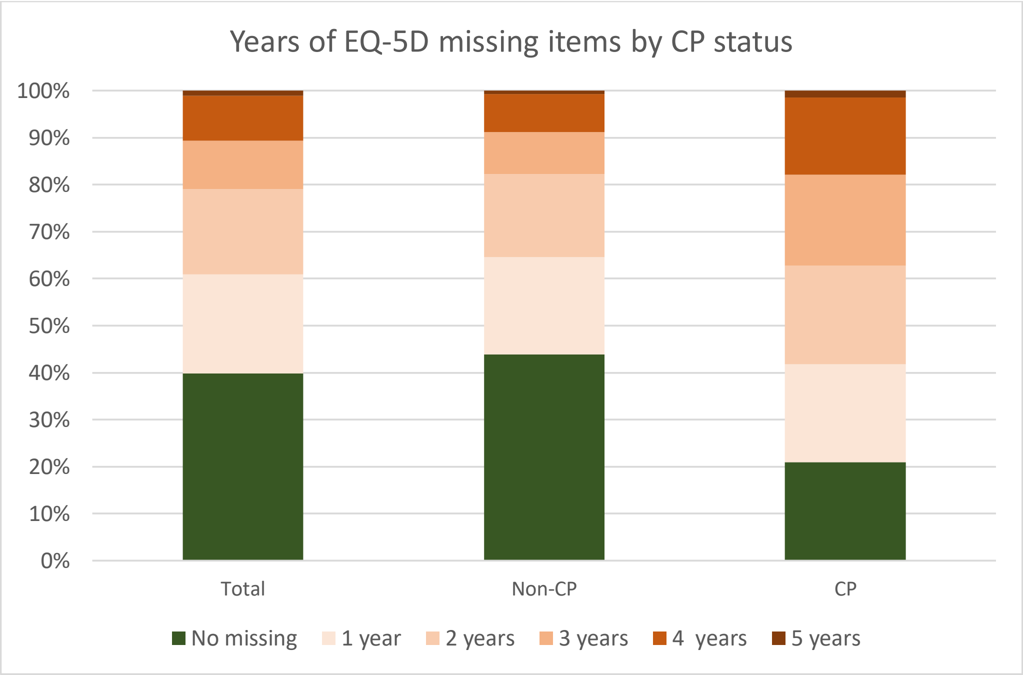
Years of EQ-5D missing items by CP status
100%
90%
80%
70%
60%
50%
40%
30%
20%
10%
0%
Total
Non-CP
CP
No missing
1 year
2 years
3 years
4 years
5 years

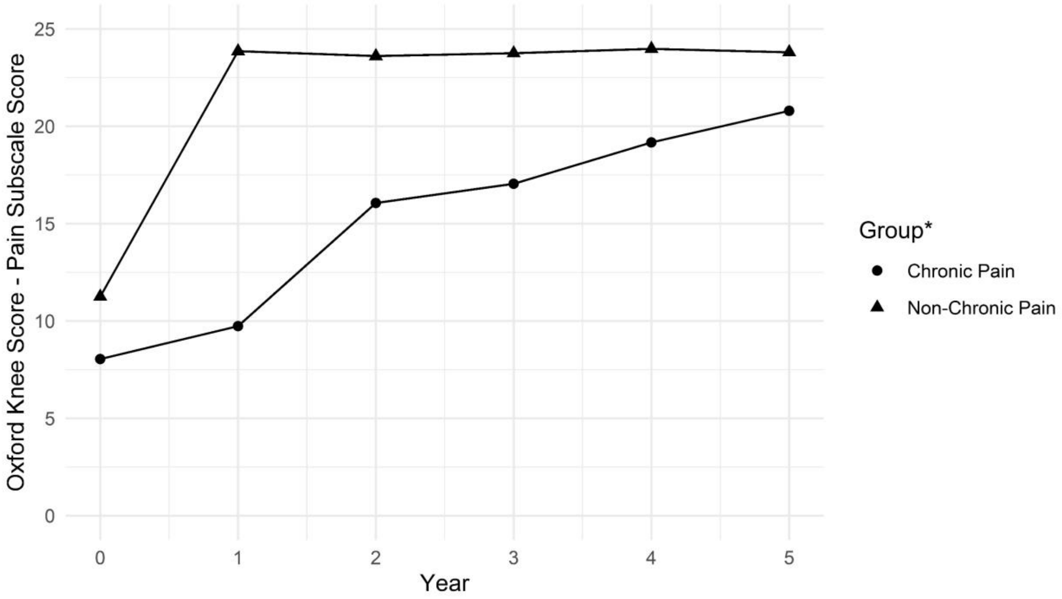
Oxford Knee Score - Pain Subscale Score
25
20
15
10
5
0
0
1
2
3
4
5
Year
Group*
Chronic Pain
Non-Chronic Pain
