# Supplementary material for: Progression of chronic pain and associated health-related quality of life and healthcare resource use over 5 years after total knee replacement: evidence from a cohort study
Source: BMJ Open. 2022 Apr 25;12(4):e058044. doi: 10.1136/bmjopen-2021-058044 (PMC9039409; doi:10.1136/bmjopen-2021-058044)
Supplement: Author's manuscript [file bmjopen-2021-058044.draft_revisions.pdf]

BMJ Open is committed to open peer review. As part of this commitment we make the peer review history of every article we publish publicly available.

When an article is published we post the peer reviewers' comments and the authors' responses online. We also post the versions of the paper that were used during peer review. These are the versions that the peer review comments apply to.

The versions of the paper that follow are the versions that were submitted during the peer review process. They are not the versions of record or the final published versions. They should not be cited or distributed as the published version of this manuscript.

BMJ Open is an open access journal and the full, final, typeset and author-corrected version of record of the manuscript is available on our site with no access controls, subscription charges or pay-per-view fees (http://bmjopen.bmj.com).

If you have any questions on BMJ Open's open peer review process please email info.bmjopen@bmj.com

BMJ Open

Progression of chronic pain, quality of life and healthcare resource use over 5 years after total knee replacement: findings from an observational cohort

Journal:	*BMJ Open*
Manuscript ID	bmjopen-2021-058044
Article Type:	Original research
Date Submitted by the Author:	05-Oct-2021

Complete List of Authors:	Cole, Sophie; University of Oxford, Nuffield Department of Orthopaedics, Rheumatology and Musculoskeletal Sciences<br>Kolovos, Spyros; University of Oxford, Nuffield Department of Orthopaedics, Rheumatology and Musculoskeletal Sciences<br>Soni, Anushka; University of Oxford, Nuffield Department of Orthopaedics, Rheumatology and Musculoskeletal Sciences<br>Delmestri, Antonella; University of Oxford, Nuffield Department of Orthopaedics, Rheumatology and Musculoskeletal Sciences<br>Sanchez Santos, Maria ; University of Oxford, Nuffield Department of Orthopaedics, Rheumatology and Musculoskeletal Sciences<br>Judge, Andrew; University of Oxford, Nuffield Department of Orthopaedics, Rheumatology and Musculoskeletal Sciences; University of Bristol, National Institute for Health Research Bristol Biomedical Research Centre<br>Arden, Nigel; University of Oxford, Nuffield Department of Orthopaedics, Rheumatology and Musculoskeletal Sciences<br>Beswick, Andrew; University of Bristol, National Institute for Health Research Bristol Biomedical Research Centre; University of Bristol, Musculoskeletal Research Unit, Translational Health Sciences, Bristol Medical School<br>Wylde, Vikki; University of Bristol, National Institute for Health Research Bristol Biomedical Research Centre; University of Bristol, Musculoskeletal Research Unit, Translational Health Sciences, Bristol Medical School<br>Gooberman-Hill, Rachael; University of Bristol, National Institute for Health Research Bristol Biomedical Research Centre; University of Bristol, Musculoskeletal Research Unit, Translational Health Sciences, Bristol Medical School<br>Pinedo-Villanueva, Rafael; University of Oxford, Nuffield Department of Orthopaedics, Rheumatology and Musculoskeletal Sciences

Keywords:	HEALTH ECONOMICS, Health policy < HEALTH SERVICES ADMINISTRATION & MANAGEMENT, Quality in health care < HEALTH SERVICES ADMINISTRATION & MANAGEMENT, Knee < ORTHOPAEDIC & TRAUMA SURGERY, PAIN MANAGEMENT, EPIDEMIOLOGY

SCHOLARONE™
Manuscripts

I, the Submitting Author has the right to grant and does grant on behalf of all authors of the Work (as defined in the below author licence), an exclusive licence and/or a non-exclusive licence for contributions from authors who are: i) UK Crown employees; ii) where BMJ has agreed a CC-BY licence shall apply, and/or iii) in accordance with the terms applicable for US Federal Government officers or employees acting as part of their official duties; on a worldwide, perpetual, irrevocable, royalty-free basis to BMJ Publishing Group Ltd ("BMJ") its licensees and where the relevant Journal is co-owned by BMJ to the co-owners of the Journal, to publish the Work in this journal and any other BMJ products and to exploit all rights, as set out in our licence.

The Submitting Author accepts and understands that any supply made under these terms is made by BMJ to the Submitting Author unless you are acting as an employee on behalf of your employer or a postgraduate student of an affiliated institution which is paying any applicable article publishing charge ("APC") for Open Access articles. Where the Submitting Author wishes to make the Work available on an Open Access basis (and intends to pay the relevant APC), the terms of reuse of such Open Access shall be governed by a Creative Commons licence – details of these licences and which Creative Commons licence will apply to this Work are set out in our licence referred to above.

Other than as permitted in any relevant BMJ Author's Self Archiving Policies, I confirm this Work has not been accepted for publication elsewhere, is not being considered for publication elsewhere and does not duplicate material already published. I confirm all authors consent to publication of this Work and authorise the granting of this licence.

For peer review only

Progression of chronic pain, quality of life and healthcare resource use over 5 years after total knee replacement: findings from an observational cohort

Sophie Cole, MSc[1], Spyros Kolovos, PhD[1], Anushka Soni, DPhil, BMBCh, MA(Hons)[1], Antonella Delmestri, PhD[1], Maria T Sanchez-Santos, MSc[1], Andrew Judge, PhD[1,2], Nigel Arden, MD[1], Andrew Beswick, BSc[2,3], Vikki Wylde, PhD[2,3], Rachael Gooberman-Hill, PhD[2,3], Rafael Pinedo-Villanueva, PhD[1]

Corresponding author: Rafael Pinedo-Villanueva[1]

Email: rafael.pinedo@ndorms.ox.ac.uk

Postal address: NDORMS, Botnar Research Centre, Old Road, Oxford, OX3 7LD

[1] Nuffield Department of Orthopaedics, Rheumatology and Musculoskeletal Sciences, Nuffield Orthopaedic Centre, University of Oxford, Windmill Road, Oxford, OX3 7LD, UK

[2] National Institute for Health Research Bristol Biomedical Research Centre, University Hospitals Bristol NHS Foundation Trust and University of Bristol, Bristol, UK

[3] Musculoskeletal Research Unit, Translational Health Sciences, Bristol Medical School, University of Bristol, Learning and Research Building, Level 1, Southmead Hospital, Bristol, BS10 5NB, UK

Word count: 4443

For peer review only

ARTICLE SUMMARY

Strengths and limitations of this study

- A strength of the study is that participant-level data were collected over 5 years after TKR enabling a detailed analysis of changes over time.
- The study cohort offered data on patient-reported outcome measures, allowing for a classification of participants according to their chronic pain status following surgery, and linked healthcare resource use to include in the analysis.
- This study followed pain trajectories of participants with post-surgical CP which has not previously been explored.
- An important limitation was that many follow-up questionnaires were not returned, which generated an important level of missing data, addressed using standard methods of multiple imputation.
- The longitudinal study questionnaires did not ask participants about informal care, productivity losses, or their use of privately funded healthcare other than physiotherapists, which may play an important role in associated costs.

For peer review only

ABSTRACT

Objective: As part of the STAR Programme, we investigated pain and function, health-related quality of life (HRQL), and healthcare resource use over 5 years after total knee replacement (TKR), comparing those with and without chronic pain 1-year post-surgery.

Methods: We used data from the COASt prospective cohort study, which followed patients undergoing TKR from two English hospitals for 5 years. Chronic pain was defined using the Oxford Knee Score pain subscale (OKS-PS) 1-year post-surgery. Pain and function were measured with the OKS, HRQL using the EQ-5D, resource use from yearly questionnaires, and costs estimated from a healthcare system perspective. We analysed the changes in OKS-PS, HRQL and resource use over a 5-year follow-up period. Multiple imputation accounted for missing data.

Results: Chronic pain was reported in 70/552 operated knees (12.7%) 1-year post-surgery. The chronic pain group had worse pain, function, and HRQL pre- and post-surgery than the non-chronic pain group. Those without chronic pain markedly improved right after surgery, then plateaued. Those with chronic pain improved slowly but steadily. Participants with chronic pain reported greater healthcare resource use and costs than those without, especially 1-year post-surgery, and mostly from hospital readmissions. 64.7% of those in chronic pain recovered during the following 4 years, while 30.9% fluctuated in and out of chronic pain.

Conclusion: Although TKR is often highly beneficial, some patients experienced chronic pain post-surgery. Although many recovered over time, identifying people most likely to have chronic pain and supporting their recovery would benefit patients and healthcare systems.

For peer review only

INTRODUCTION

Total knee replacement (TKR) provides pain relief and increases function for many people with osteoarthritis, which improves quality of life. However, a distinct, important group of people report long-term (chronic) pain after their TKR [1-5]. Chronic post-surgical pain is defined as pain that persists at least 3 months after surgery, beyond the healing process [6]. There is currently limited understanding of how chronic pain (CP) changes over time, shapes people's lives, and affects quality of life and healthcare service use.

Although pain trajectories after TKR have previously been explored [7], those who report post-surgical CP have not been followed specifically. It's not yet known what proportion of individuals who experience CP in the first year after TKR find that their pain dissipates over the following few years, remain in CP, or find that their pain fluctuates. As CP is a complex construct, it would be useful to compare people who transition between these groups to understand whether they are more similar to the group they left or the group they joined. Better understanding of CP trajectories would help discern how CP evolves, giving patients clearer information about how likely they are to recover from their CP and helping clinicians to design effective interventions to treat or manage CP.

Assessing clinical and cost-effectiveness is a critical step in informing decision-making about the wider implementation of new interventions. For cost-effectiveness studies to be carried out, our understanding of the progression of CP must be accompanied by an assessment of health-related quality of life (HRQL) and costs. HRQL is affected by the severity of pain and its consequences and is expected to vary as people's pain improves, worsens, or fluctuates. Changes in pain are also likely to impact healthcare use, such as follow-up hospital visits, GP appointments, or prescriptions of analgesia. Healthcare resource use and costs after a TKR have been previously described [8 9] but to our knowledge these have not been examined

For peer review only

specifically for those in CP, hence leaving an important research gap which needs to be closed to enable cost-effectiveness assessments of interventions addressing CP after TKR. This study aimed to characterise pain and function, HRQL, and healthcare resource use by patients over the 5 years after TKR, comparing those with and without CP 1-year after surgery, using data collected in a published prospective cohort study [9]. It aimed to map how the CP status of those with CP changed and assess how changes in HRQL and healthcare costs for those recovering from CP after the first year compared to those who did not recover or never had CP.

PATIENTS AND METHODS

COASt

The Clinical Outcomes in Arthroplasty Study (COASt), a prospective cohort study, tested outcome prediction models' performance [9] by collecting pre- and post-operative outcome data from patients undergoing hip and knee replacements with yearly follow-up. It was extended to 5 years to capture long-term pain and function, quality of life, and healthcare resource use.

COASt recruited patients receiving a knee replacement from the Nuffield Orthopaedic Centre in Oxford from 2010 and the Southampton General Hospital from 2011 (both in England). Patients were invited to participate after they were placed on the waiting list for a knee replacement. Baseline information was collected in pre-operative questionnaires that covered participant demographics such as the Index of Multiple Deprivation (IMD) (a measure of relative deprivation at a small local area level in England) and patient-reported outcome measures (PROMs): HRQL (EuroQuol EQ5D-3L) and pain and function (Oxford Knee

For peer review only

Score, OKS). COASt participants were followed-up with yearly postal questionnaires for 5 years post-surgery.

We included COASt participants who underwent TKR, returned their questionnaires 1-year after surgery, and completed the OKS pain subscale in that questionnaire for the resource use analysis. For the pain, function, and HRQoL analysis we also included participants who did not complete their OKS-PS at 1-year, in which cases missing data were imputed. The COASt study was approved by the Oxford Research Ethics Committee A (reference number 10/H0604/91). Patients listed for hip or knee replacement surgeries in the two participating centres (Southampton and Oxford) were potentially eligible for inclusion. Once identified, potential participants were sent a recruitment pack, consisting of a patient information sheet, a sample consent form, and a recruitment letter. They were later contacted by telephone by a member of the COASt team around two weeks after the pack was sent to discuss study details and answer any question they might have had. The COASt team member then took verbal consent if patients were satisfied and willing to participate in the study [9]. Research complied with the Helsinki Declaration.

Patient and Public Involvement

Patient and Public Involvement (PPI) members were actively involved in the design and recruitment of the STAR programme. PPI members participated in over 20 meetings of the STAR Patient Forum to discuss and improve the participant experience. They gave feedback and recommended changes for both recruitment and study materials for the STAR trial, which was further informed by the results of this study. Examples of this included recommended improvements for plain English summaries of reported results, the length of questionnaires, and phoning patients who had not completed the questionnaire instead of sending another one. The corresponding author met face-to-face with PPI members to

For peer review only

discuss the methods and preliminary findings of this analysis prior to completion, and they contributed new questions of interest which were later adopted and included in the study. Findings were disseminated to study participants via regular programme bulletins and through STAR Patient Forum meetings.

Pain and function

Pain and functional ability were measured with the OKS, a patient-reported questionnaire consisting of 12 items that assesses a patient's perspective of their joint pain and function after a TKR [10]. Each item has five possible responses, with higher values indicating better outcomes, and contributes 0-4 to the total score, which ranges 0-48.

The OKS pain subscale (OKS-PS) was used to identify those in CP, following work by some of our co-authors. The OKS-PS includes the seven OKS questions relating to pain and is summarised by a score of 0-28 [11]. Participants reporting an OKS-PS of 14 or lower 1-year after surgery were classified as having CP, while those scoring above 14 were classified as non-CP [4].

Quality of life

HRQL was measured using the EQ5D-3L questionnaire, which examines five dimensions of health: mobility, self-care, usual activities, pain/discomfort, and anxiety/depression. This instrument was completed at baseline (just before surgery) and annually after the TKR for 5 years. A health utility estimate, anchored at 0 representing death and with 1 representing full health, was calculated by applying a social preference tariff collected from a sample of the UK general population [12].

Healthcare resource use

Healthcare resource use was measured using participants' answers to questions about their visits to general practitioners, nurses, physiotherapists, alternative practitioners, and

For peer review only

admissions to hospitals, because of their operated knee. These questions were included in the baseline and all follow-up questionnaires and covered the preceding 12 months.

The value of healthcare resources was estimated from the NHS perspective by applying mean unit costs sourced from the Personal Social Services Research Unit [13] and National Cost Collection [14]. To calculate the cost of readmittance to hospital, we used a binary variable and information from a free text section in the questionnaire explaining the readmittance. The submitted free text was used to identify participants who were treated for a knee infection or underwent a knee procedure. Unit costs were applied according to this categorisation and costs estimated by taking a weighted average across a number of reported knee procedures and treatments for knee infections [14].

Missing data

Loss to follow-up over the 5 years led to increasing amounts of missing data. Multiple imputation assuming data missing at random was implemented to address the missing data. Fifty imputed datasets were generated, with missing OKS-PS and EQ-5D values replaced by imputed ones [15]. We used the imputation by chain equation and applied the predictive mean matching two-level imputation method to account for each participant completing the follow-up questionnaires multiple times. To ensure that imputation did not introduce bias into the results, we compared the outcome distribution in the observed and imputed datasets [16]. We also analysed the participant demographics for the observed and missing data at year 5 follow-up, how many years the participants had missing OKS-PS or EQ-5D values, and investigated our assumption of the missing data being missing at random with logistic regression. We censored participants who died within the 5 years of follow-up (n = 30) so that we did not impute data beyond their death. Summary statistics for the imputed dataset's outcomes were combined with Rubin's rule [17].

For peer review only

Analysis

We characterised outcomes, resource use, and costs by CP group (CP and non-CP, based on 1-year post-operative outcomes) over 5 years by reporting the mean and standard deviation of the imputed OKS, OKS-PS, health utility, number of healthcare visits, and healthcare costs from pre-surgery to 5 years post-surgery.

To investigate the trajectory of CP, we tracked CP-group participants' movement in and out of CP and report the percentage that remained in CP for the entire period, recovered from CP at any point, and fluctuated between CP and non-CP over the 5 years. A participant's CP group status at each time point was determined by their observed OKS-PS, if available, or the mean across the 50 imputed scores, if missing. Transitions in and out of CP are illustrated in a Sankey diagram.

To investigate whether leaving the CP group after year 1 led to changes in quality of life or costs, we compared observed health utility and healthcare costs of CP-group participants who recovered from CP by the second year after surgery and those who did not.

All analyses were conducted in R [18] using multiple packages for data cleaning and statistical analysis [19-26], performing multiple imputation [27-30], and producing figures [31-33]. Figure 2 was created with Tableau Desktop 2020.2. We followed the STROBE statement as a reporting guideline for this study [34].

RESULTS

Of the 1,025 knee procedures enrolled in COASt, all 580 corresponding to a TKR returned their year-1 follow-up questionnaire. We excluded 28 who did not report their OKS-PS in that questionnaire, giving a final sample of 552. Seventy (of 552, 12.7%) were classified as in

For peer review only

CP 1-year after surgery using the OKS-PS. We refer to those classified as in CP 1-year after TKR as the CP group, and those not in CP 1-year after TKR as the non-CP group.

Table 1 shows that the CP and non-CP groups had similar demographics. Both had a mean age of 70 years at surgery and similar mean BMIs, although higher in the CP group. A greater proportion of the CP group were female (69%) than the non-CP group (54%). Both groups comprised a higher proportion of people living in less deprived areas than the national distribution. The CP group reported worse pre-operative pain, function, and HRQL scores than the non-CP group (Table 2). The amount of missing data due to loss to follow-up is reported in Tables 1 and 2.

For peer review only

Table 1: Study participants demographics

Table 1 – Study participant demographics

**Variable**	**CP at Y1 (n = 70)**			**Non-CP at Y1 (n = 482)**			**Total at Y1 (n = 552)**		
**Age at total knee replacement (n, mean, (range))**									
Total	70	70	(42-88)	482	70	(38-90)	552	70	(38-90)
Below 50	*1*	*42*	*(42)*	*8*	*43*	*(38-47)*	*9*	*43*	*(38-47)*
50-60	*6*	*55.8*	*(54-58)*	*47*	*56*	*(50-60)*	*53*	*56*	*(50-60)*
60-70	*28*	*66*	*(61-70)*	*182*	*66*	*(61-70)*	*210*	*66*	*(61-70)*
70-80	*27*	*75*	*(71-79)*	*200*	*75*	*(71-80)*	*227*	*75*	*(71-80)*
Above 80	*8*	*84*	*(81-88)*	*45*	*84*	*(81-90)*	*53*	*84*	*(81-90)*
**Gender (n, %)**									
Female	48	68.6		260	53.9		308	55.8	
Male	22	31.4		222	46.1		244	44.2	
**IMD 2010 decile (n, %)**									
1 (least deprived)	15	21.4		107	22.2		122	22.1	
2	4	5.7		93	19.3		97	17.6	
3	8	11.4		77	16.0		85	15.4	
4	9	12.9		48	10.0		57	10.3	
5	12	17.1		43	8.9		55	10.0	
6	12	17.1		43	8.9		55	10.0	
7	3	4.3		40	8.3		43	7.8	
8	0	0.0		14	2.9		14	2.5	
9	5	7.1		13	2.7		18	3.3	
10 (most deprived)	2	2.9		3	0.6		5	0.9	
Missing (n, (%))	*0*	*(0.0)*		*1*	*(0.2)*		*1*	*(0.2)*	
**BMI (n, mean, (SD))**									
Total	70	32.4	(5.9)	478	30.4	(5.4)	548	30.7	(5.5)
Below 18.5	1	18.0	(0.0)	0	0.0	(0.0)	1	18.0	(0.0)
18.5 - 24.9	5	23.0	(1.7)	70	23.1	(1.5)	75	23.1	(1.5)
25 - 29.9	18	27.9	(1.4)	181	27.6	(1.4)	199	27.6	(1.4)
30 - 34.9	21	32.0	(1.5)	127	32.2	(1.4)	148	32.2	(1.4)
35 - 39.9	21	37.2	(1.6)	75	37.1	(1.3)	96	37.1	1,36
Above 39.9	4	45.0	(6.1)	25	42.7	(3.7)	29	43.0	(4.0)
Missing (n, (%))	*0*	*(0.0%)*		*4*	*(0.8%)*		*4*	*(0.7%)*	

BMI: body mass index; CP: chronic pain; IMD: Index of Multiple Deprivation; SD: standard deviation; Y: year

For peer review only

Table 2: Study participants health outcomes

Table 2 – Study participants health outcomes

Variable	CP at Y1 (n=70)			Non-CP at Y1 (n=482)			Total at Y1 (n=552)		
**OKS (n, mean, (SD))**									
Baseline	62	14.1	(6.8)	428	20.2	(7.5)	490	19.4	(7.7)
Missing (n, (%))	*8*		*(11.4%)*	*54*		*(11.2%)*	*62*		*(11.2%)*
Year 1	67	16.9	(6.0)	472	38.9	(6.9)	539	36.2	(10.0)
Missing (n, (%))	*3*		*(4.3%)*	*10*		*(2.1%)*	*13*		*(2.4%)*
Year 3	44	22.1	(8.7)	391	39.6	(7.8)	435	37.8	(9.5)
Missing (n, (%))	*26*		*(37.1%)*	*91*		*(18.9%)*	*117*		*(21.2%)*
Year 3	41	24.4	(9.3)	345	39.8	(7.4)	386	38.2	(9.0)
Missing (n, (%))	*29*		*(41.4%)*	*137*		*(28.4%)*	*166*		*(30.1%)*
Year 4	30	26.0	(10.4)	290	39.9	(7.6)	320	38.6	(8.9)
Missing (n, (%))	*40*		*(57.1%)*	*192*		*(39.8%)*	*232*		*(42.0%)*
Year 5	20	25.3	(9.6)	254	39.4	(8.3)	274	38.3	(9.2)
Missing (n, (%))	*50*		*(71.4%)*	*228*		*(47.3%)*	*278*		*(50.4%)*
**OKS-PS (n, mean, (SD)))**									
Baseline	62	7.1	(4.0)	431	10.6	(4.6)	493	10.2	(4.7)
Missing (n, (%))	*8*		*(11.4%)*	*51*		*(10.6%)*	*59*		*(10.7%)*
Year 1	70	9.7	(3.6)	482	23.8	(4.0)	552	22.0	(6.1)
Missing (n, (%))	*0*		*(0.0%)*	*0*		*(0.0%)*	*0*		*(0.0%)*
Year 2	44	13.4	(5.8)	404	24.2	(4.6)	448	23.1	(5.7)
Missing (n, (%))	*26*		*(37.1%)*	*78*		*(16.2%)*	*104*		*(18.8%)*
Year 3	41	14.8	(6.1)	361	24.3	(4.5)	402	23.3	(5.5)
Missing (n, (%))	*29*		*(41.4%)*	*121*		*(25.1%)*	*150*		*(27.2%)*
Year 4	31	15.9	(6.6)	310	24.5	(4.5)	341	23.7	(5.4)
Missing (n, (%))	*39*		*(55.7%)*	*172*		*(35.7%)*	*211*		*(38.2%)*
Year 5	21	15.7	(5.9)	265	24.1	(5.0)	286	23.5	(5.5)
Missing (n, (%))	*49*		*(70.0%)*	*217*		*(45.0%)*	*266*		*(48.2%)*
**Health utility estimate (n, mean, (SD)))**									
Baseline	64	0.2732	(0.315)	430	0.4789	(0.283)	494	0.452	(0.295)
Missing (n, (%))	*6*	*(8.6%)*		*52*	*(10.8%)*		*58*	*(10.5%)*	
Year 1	67	0.3857	(0.310)	471	0.7909	(0.199)	538	0.740	(0.254)
Missing (n, (%))	*3*	*(4.3%)*		*11*	*(2.3%)*		*14*	*(2.5%)*	
Year 2	47	0.4422	(0.321)	402	0.8040	(0.235)	449	0.766	(0.269)
Missing (n, (%))	*23*	*(32.9%)*		*80*	*(16.6%)*		*103*	*(18.7%)*	
Year 3	41	0.4209	(0.314)	357	0.7987	(0.216)	398	0.760	(0.255)
Missing (n, (%))	*29*	*(41.4%)*		*125*	*(25.9%)*		*154*	*(27.9%)*	
Year 4	31	0.4953	(0.298)	305	0.7877	(0.247)	336	0.761	(0.265)
Missing (n, (%))	*39*	*(55.7%)*		*177*	*(36.7%)*		*216*	*(39.1%)*	
Year 5	23	0.5398	(0.326)	266	0.7748	(0.257)	289	0.756	(0.270)
Missing (n, (%))	*47*	*(67.1%)*		*216*	*(44.8%)*		*263*	*(47.6%)*	

CP: chronic pain; OKS: Oxford Knee Score; OKS-PS: Oxford Knee Score pain subscale; SD: standard deviation; Y: year

For peer review only

Characterisation of outcomes, resource use, and costs

Observed and imputed data showed similar mean health utility and OKS-PS scores (Electronic Supplementary Material Table A.1 and Figures A.1 and A.2). Observed and missing data at year 5 of follow-up reported similar preoperative demographics and mean health utility and OKS-PS scores (Electronic Supplementary Material Table A.2). 77.9% of participants had between zero and 2 missing years of OKS-PS across the 5 years of follow-up (Supplementary material Table A.3). Those in CP reported a higher number of years of missing items for both OKS-PS and EQ-5D (Electronic Supplementary Material Figures A.3and A.4). Logistic regression showed that dimensions of EQ-5D and OKS-PS measured preoperatively had a statistically significant association with the missing OKS-PS across the 5 years of follow-up (Electronic Supplementary Material Table A.4).

The CP and non-CP groups had different health utility scores before surgery (Figure 1). Participants in CP 1-year after TKR had a pre-operative health utility estimate of 0.307, whereas those who would not be in CP had a pre-operative score of 0.485. The two groups had noticeably different changes in health utility over the next 5 years. On average, the non-CP group improved significantly during the first year after surgery, reaching a score of 0.787, and stayed at a similarly high level until follow-up ended (0.751 after 5 years). The CP group slowly improved their average health utility (except between years 2 and 3), going from 0.399 1-year after surgery to 0.656 5 years after surgery (Figure 1 and Electronic Supplementary Material Table A.5).

Figure 1: EQ5D-3L scores over time

There were similar patterns in pain progression, measured with the OKS-PS (Electronic Supplementary Material Figure A.5). The non-CP group started with a higher average pre-operative score (11.3), improved significantly during the first year after surgery (23.9), and

then stabilised (23.8 after 5 years). The CP group started with a lower average pre-operative score (8.0) and slowly but steadily improved, from 9.7 after 1-year to 20.8 after 5 years (Electronic Supplementary Material Table A.6).

Tables A.7a-A.8b in the Electronic Supplementary Material show participants' use of healthcare resources. In the year before surgery, the groups made comparable numbers of visits to an NHS general practitioner (GP), with the CP group reporting an average 3.8 visits and the non-CP group an average 3.4. However, their use patterns differed after surgery. One year after surgery, mean yearly visits were 2.7 for the CP group and 0.6 for the non-CP group. The mean number of NHS GP visits then fell steadily for both groups to an average of 0.3 visits for the CP group and 0.1 for the non-CP group 5 years after surgery.

The groups' average annual number of visits to physiotherapists, hospital doctors, nurses, and alternative practitioners differed by healthcare specialist, but showed similar patterns. The CP and non-CP groups made similar numbers of visits before surgery; numbers surged for both groups during the 12 months after surgery, especially for the CP group. Visits then progressively decreased over the rest of the follow-up period for both groups. Although most physiotherapy visits were to NHS practitioners, during the first year post-surgery the CP group reported an average of 4 visits to NHS physiotherapists and 3 visits to private physiotherapists, whereas the non-CP group respectively made 2.2 and 0.6 visits. The groups' number of visits to A&E, readmissions to the same hospital, and admission to any other hospital all showed similar patterns over time as the visits to non-GP healthcare professionals.

Participants in the two groups had similar mean healthcare costs in the year before surgery: £430 (SD = 611.41) for the CP group and £322 (SD = 337.22) for the non-CP group. Figure 2 shows the change in mean yearly costs by category over time. Consistent with the changes

For peer review only

in resource use, mean costs increased to £1,799 (SD = 2981.69) for those in the CP group 1-year after TKR and £501 (SD = 1511.82) for those in the non-CP group. Readmission to hospital was responsible for 75% of the differences between the groups (£973 of £1,298) during this first year after surgery. After the first year, mean healthcare costs fell steadily for both cohorts. Electronic Supplementary Material Tables A.9a and A.9b show yearly healthcare costs by CP status from pre-operation to 5 years after TKR.

Figure 2 – Yearly healthcare costs by chronic pain (CP) status 1-year after total knee replacement surgery

Progression of chronic pain status

Figure 3 shows the change in CP status over the 5 years after TKR, based on yearly OKS-PS scores. Only 4.4% of those who reported experiencing CP after 1-year remained in CP throughout the 5 years. Almost a third of participants (30.9%) fluctuated in and out of CP. Most (64.7%) of those in CP 1-year post-surgery left CP within the next 4 years and did not experience CP again. Most of those who recovered from CP did so during the second year after surgery (26/44), with fewer people leaving CP each year thereafter except for the final year.

Figure 3: Sankey diagram of those in CP 1-year after surgery

Changes between year one and year two

Mean health utility remained stable for those who remained in the same chronic pain group between years 1 and 2, whilst those who moved into CP saw their health utility drop and those who recovered from it reported a clear improvement (Table 3).

For peer review only

Table 3: Mean health outcomes for fluctuating and stable groups over chronic pain (CP) status between 1 and 2 years after total knee replacement

Table 3: Mean health outcomes for fluctuating and stable groups over chronic pain (CP) status between 1 and 2 years after total knee replacement

	n	Mean	SD
**Health utility estimate**			
Year 1 CP to Year 2 CP			
Year 1	25	0.365	0.308
Year 2	25	0.326	0.328
Year 1 CP to Year 2 Non-CP			
Year 1	17	0.524	0.276
Year 2	19	0.619	0.216
Year 1 Non-CP to Year 2 Non-CP			
Year 1	374	0.811	0.189
Year 2	368	0.826	0.212
Year 1 Non-CP to Year 2 CP			
Year 1	21	0.604	0.175
Year 2	21	0.461	0.317
**OKS-PS**			
Year 1 CP to Year 2 CP			
Year 1	25	9.4	3.73
Year 2	25	9.4	3.93
Year 1 CP to Year 2 Non-CP			
Year 1	19	11.2	3.08
Year 2	19	18.7	2.75
Year 1 Non-CP to Year 2 Non-CP			
Year 1	382	24.3	3.63
Year 2	382	24.9	3.56
Year 1 Non-CP to Year 2 CP			
Year 1	22	17.2	2.48
Year 2	22	11.7	2.34

OKS-PS: Oxford Knee Score pain subscale; SD: standard deviation

For peer review only

The groups' healthcare costs followed similar patterns to their health utility scores (Electronic Supplementary Material Table A.10 and Table A.11). Participants who remained in CP had the highest costs in both the first (£1,600) and second (£1,450) years post-surgery. Those who recovered from CP after the first year reported a drop in costs from £1,500 in the first year to £1,000 in the second year. Those who developed CP in the second year saw their costs drop from £1,300 to £1,200. Those who remained free of CP had the lowest costs in both the first (£481) and second (£133) years.

DISCUSSION

Our analysis of data from COASt found that, on average, TKR led to large HRQL improvements, with health utility scores improving from 0.447 before surgery to 0.742 1-year after surgery. This improvement is consistent with what has been reported in previous studies [35], including average health utility gains of 0.334 over 6 months in 2018/2019 reported by the national English NHS PROMs [36]. However, these results were for all participants and hide the experience of those in CP for months after TKR. We found that 12.7% of participants reported CP 1-year after surgery, which is consistent with previous studies that used different mechanisms to ascertain CP [1-3 5].

Our characterisation of pain and function, HRQL, and healthcare resource use and costs by participants with and without CP 1-year after surgery suggests that these groups are distinct. However, confidence intervals are wide and overlap. Study participants in the non-CP group reported rapid, significant improvements in OKS-PS, OKS, and EQ-5D over the 5 years after surgery, whereas those with CP improved much less and much more slowly. It's not surprising that the pain, composite pain and function, and HRQL measures changed in similar ways, given their close association [37]. A key distinction between the groups was their

For peer review only

pre-operative health status. Those in the CP group had lower pre-operative scores, indicating worse health, than those without CP for pain (mean OKS-PS = 8 and 11, respectively) and HRQL (mean health utility = 0.307 and 0.485, respectively). Those in the CP group accessed more community healthcare and hospital services than those in the non-CP group at all time points, most notably during the first year after surgery (average healthcare costs: £1,800 for CP, £500 for non-CP). The main driver for the difference was readmission to the same hospital. Although the questionnaires used in COASt did not record the reason for readmissions, they were likely due to re-operations.

Two-thirds (64.7%) of participants with CP recovered and were no longer in CP within 5 years after surgery. This result suggests that CP after TKR is not a permanent condition and can improve with time as patients access routine care. This improvement could have been linked to individual characteristics, day-to-day life, or the severity of the original condition. Most of those who recovered from CP did so during the second year after surgery. This longer time to resolve post-surgical pain may have been due to recovery of different pain mechanisms, such as centrally mediated pain sensitisation present before surgery [38 39]. Altered brain morphology associated with pain in osteoarthritis has been shown to be reversible after arthroplasty [40].

Only 4.4% of participants reported OKS-PS scores that indicated CP for all 5 years after surgery. In a 2007 study, Brander et al. [41] also found most patients with heightened, unexplained pain 1-year after knee replacement demonstrated subsequent improvement over several years. They found that depression was an important determinant of long-term outcomes.

A third (30.9%) of participants reported fluctuating OPS-PS scores that classified them as experiencing CP at only certain points during the study period. Relief from CP may not

For peer review only

always be permanent, which is consistent with the variability in temporal fluctuation in knee pain seen without surgical intervention [42]. Although most participants eventually recovered from CP and made associated gains in HRQL, those who experienced CP 1-year after surgery did not reach the same level of health utility 5 years after surgery as without CP (0.659 vs 0.787, respectively). To our knowledge, this is the first study applying a CP criterion to examine whether and when people with CP after TKR recover.

Participants in CP after the first year after surgery who recovered by the second year improved their health utility more than those who stayed in CP, but not as much as those who were not in CP in either year. Improved OKS-PS is a sign of less pain. As it's likely associated with improved mobility, self-care, and conduct of usual activities, it would be expected to lead to improved EQ-5D scores. However, participants switching CP groups also reported changes in use of healthcare resources that separated them from the CP group and brought them closer, albeit not directly in line with, participants without CP. These findings suggest progressive improvement as people come out of CP. The OKS-PS cut-off used to identify CP may therefore be sensitive enough to identify distinct groups as they transition between CP categories.

Our results suggest that under current clinical practice, many patients leave CP over time by gradually improving. Identifying these patients early will allow clinicians to provide them with tailored support to make that transition as quickly as possible. Better understanding which patients remain in CP or only recover after many years of struggling with pain will also help clinicians to design interventions to improve and manage these patients' pain, allow them to continue with their lives with the least interference possible.

This study has several potential limitations. As our analyses used data collected from patients recruited from two hospitals in Southampton and Oxford, the findings are not

For peer review only

necessarily generalisable to the rest of the country. However, the participants' health gains after TKR agreed with those reported by the NHS PROMs programme[36], which invites all patients undergoing a knee replacement funded by the NHS in England to take part, suggesting a degree of generalisability.

Our categorisation of CP was made using an OKS-PS cut-off score. As pain is a complex construct, it might not be appropriate to dichotomise it via a threshold on an instrument designed to measure improvement after knee replacement, rather than capturing the diverse dimensions of the pain experience. However, the use of this cut-off point to identify people with and without CP effectively distinguished between groups with significantly different health outcomes, gains, resource use, and healthcare costs [4], which can impact future guidelines for clinical care for people with CP.

The questionnaires used in COASt resulted in many study limitations, only some of which could be mitigated against. Many follow-up questionnaires were not returned, which generated an important level of missing data. It's difficult to know the direction of the impact this may have had as participants may not return questionnaires for multiple reasons, including both being very dissatisfied with their surgery or doing so well they could not be bothered to respond. We addressed this issue by applying multiple imputation to reduce bias and improve our study's power [16 43]. We also analysed the observed and missing data at 5-year follow-up and did not find a significant difference between the groups. Previous research has shown that assuming that missing data are either missing at random or missing completely at random, even with loss to follow-up of 60%, does not necessarily lead to significant bias of the results [44]. Research has also shown that loss to follow-up can be predicted by measures of pain and functioning, which supports our findings that dimensions of EQ-5D and OKS-PS are associated with missing data [45].

For peer review only

The questionnaires did not allow us to identify specific interventions received by participants after TKR, only unspecified hospital readmissions. We were therefore unable to identify whether the improvements observed were due to any particular interventions or treatments. Moreover, lacking specific intervention details was a limitation to estimate costs to the NHS, but we addressed this by using average unit costs weighted by their relative use in the NHS [14].

As the resource-use questionnaires asked patients to report events that had occurred over the previous 12 months, they were potentially subject to recall bias. However, we are confident that most hospitalisations are sufficiently significant events for participants to have recalled them all. However, this may not be the case for less significant events such as a routine GP visit.

The questionnaires did not ask participants about informal care, productivity losses, or their use of privately funded healthcare other than physiotherapists. Although these issues are relevant for TKR patients, excluding this information was consistent with the perspective adopted for this analysis, i.e. that of the healthcare payer (NHS).

This study's main strength is the rich longitudinal dataset used. Unlike the national PROMs programme, which only collects PROMs before and 6 months after surgery, COASt collected data for 5 years after TKR. It also offered data on both PROMs and healthcare resource use, allowing us to explore the patterns in both.

Further research building on our findings is warranted. Identifying how best to assess pre- and post-surgical factors may help to provide more detailed understanding of variation in and potential predictors of CP and the likelihood and speed of recovery. We believe that including measures such as neuropathic pain, catastrophising, sleep disturbance, anxiety, depression, pain medication intake, self-prescription, and physical activity, among others,

For peer review only

would help build a comprehensive, informative picture. In the meantime, clinicians can reassure patients that CP after TKR is generally not permanent and that improvement is likely for many, if not most.

CONCLUSION

We investigated patients' experience of CP and resource use after TKR. One year after TKR, 12.7% of participants reported being in CP. The significant, rapid improvements in health-related quality of life observed after TKR when examining all patients hid the slow, gradual improvement experienced by people with CP, who differed from those not in CP in terms of pain and function, health utility, healthcare resource use, and costs. Most participants eventually recovered from CP, reducing their need for healthcare resources. Further research is needed to understand who is most likely to develop CP after TKR and how best to support them in long-term recovery, benefiting patients, their families, and healthcare systems.

For peer review only

CONTRIBUTORSHIP STATEMENT

All authors contributed to the conceptualization and methodology of the study. SC, SK, AD, MSS, and RPV contributed to the data curation. SC, AS, MSS, and RPV conducted the formal analysis, and all authors wrote the original draft or contributed to the review and editing of the manuscript.

ACKNOWLEDGMENTS

We thank the participants of the COASt study in Southampton General Hospital and Nuffield Orthopaedic Centre in Oxford as well as all healthcare professionals and researchers involved. As a study conducted as part of the STAR programme, we acknowledge all members and affiliated members of the team: Aideen Ahern, Andrew Beswick, Ashley Blom, Amanda Burston, Jane Dennis, Kirsty Garfield, Rachael Gooberman-Hill, Andrew Judge, Athene Lane, Fiona MacKichan, Sian Noble, Andrew Moore, Tim Peters, Emily Sanderson, Joanne Simon, Jodi Taylor, Vikki Wylde, Paul Dieppe, Chris Eccleston, Nick Ambler, Wendy Bertram, Susan Bridgewater, Nick Howells, Leigh Morrison, Gemma Munkenbeck, Candida McCabe, Andrew Toms, Rowenna Stroud, Kate Button, Simon White, Nigel Arden, Andrew Price, Rafael Pinedo-Villanueva, Anushka Soni, David Walsh, Julie Bruce, Stewart Long, Joanne Adams, Ben Burston, Vikran Desai, Tim Board, Colin Esler, Michael Parry, and the Patient Experience Partnership in Research (PEP-R) at the University of Bristol. The STAR Programme was funded by the National Institute for Health Research (NIHR) [Programme Grant for Applied Research (Grant Reference Number RP-PG-0613-20001)] and supported by the NIHR Biomedical Research Centre at University Hospitals Bristol and Weston NHS Foundation Trust and the University of Bristol. The views expressed are those of the authors

For peer review only

and not necessarily those of the NIHR or the Department of Health and Social Care. We acknowledge English language editing by Dr Jennifer A. de Beyer of the Centre for Statistics in Medicine, University of Oxford.

COMPETING INTERESTS

Prof Arden reports consultancy fees outside the scope of this work. All other authors report no competing interests.

DATA SHARING STATEMENT

No additional data available.

REFERENCES

1. Beswick AD, Wylde V, Gooberman-Hill R, et al. What proportion of patients report long-term pain after total hip or knee replacement for osteoarthritis? A systematic review of prospective studies in unselected patients. *BMJ Open* 2012;2(1):e000435. doi: 10.1136/bmjopen-2011-000435
2. Rice DA, Kluger MT, McNair PJ, et al. Persistent postoperative pain after total knee arthroplasty: a prospective cohort study of potential risk factors. *Br J Anaesth* 2018;121(4):804-12. doi: 10.1016/j.bja.2018.05.070 [published Online First: 2018/09/22]
3. Gungor S, Fields K, Aiyer R, et al. Incidence and risk factors for development of persistent postsurgical pain following total knee arthroplasty: A retrospective cohort study. *Medicine (Baltimore)* 2019;98(28):e16450. doi: 10.1097/md.0000000000016450 [published Online First: 2019/07/16]
4. Pinedo-Villanueva R, Khalid S, Wylde V, et al. Identifying individuals with chronic pain after knee replacement: a population-cohort, cluster-analysis of Oxford knee scores in 128,145 patients from the English National Health Service. *BMC Musculoskelet Disord* 2018;19(1):354. doi: 10.1186/s12891-018-2270-9 [published Online First: 2018/10/05]
5. Vina ER, Hannon MJ, Kwoh CK. Improvement following total knee replacement surgery: Exploring preoperative symptoms and change in preoperative symptoms. *Semin Arthritis Rheum* 2016;45(5):547-55. doi: 10.1016/j.semarthrit.2015.10.002 [published Online First: 2015/11/18]
6. Schug SA, Lavand'homme P, Barke A, et al. The IASP classification of chronic pain for ICD-11: chronic postsurgical or posttraumatic pain. *Pain* 2019;160(1):45-52. doi: 10.1097/j.pain.0000000000001413 [published Online First: 2018/12/27]
7. Lenguerrand E, Wylde V, Gooberman-Hill R, et al. Trajectories of Pain and Function after Primary Hip and Knee Arthroplasty: The ADAPT Cohort Study. *PLoS One* 2016;11(2):e0149306. doi: 10.1371/journal.pone.0149306 [published Online First: 2016/02/13]

For peer review only

8. Bozic KJ, Stacey B, Berger A, et al. Resource utilization and costs before and after total joint arthroplasty. *BMC Health Serv Res* 2012;12:73. doi: 10.1186/1472-6963-12-73 [published Online First: 2012/03/27]
9. Arden N, Altman D, Beard D, et al. Lower limb arthroplasty: can we produce a tool to predict outcome and failure, and is it cost-effective? An epidemiological study. Southampton (UK): NIHR Journals Library 2017.
10. Dawson J, Fitzpatrick R, Murray D, et al. Questionnaire on the perceptions of patients about total knee replacement. *J Bone Joint Surg Br* 1998;80(1):63-9. doi: 10.1302/0301-620x.80b1.7859 [published Online First: 1998/02/14]
11. Harris K, Dawson J, Doll H, et al. Can pain and function be distinguished in the Oxford Knee Score in a meaningful way? An exploratory and confirmatory factor analysis. *Quality of life research : an international journal of quality of life aspects of treatment, care and rehabilitation* 2013;22(9):2561-8. doi: 10.1007/s11136-013-0393-x [published Online First: 2013/03/26]
12. Dolan P. Modeling valuations for EuroQol health states. *Med Care* 1997;35(11):1095-108. doi: 10.1097/00005650-199711000-00002 [published Online First: 1997/11/21]
13. Curtis L, Burns A. Unit Costs of Health and Social Care 2018, Personal Social Services Research Unit. University of Kent, Canterbury, 2018.
14. NHS. 2018/19 National Cost Collection data - National schedule of NHS costs 2020 [Available from: https://improvement.nhs.uk/resources/national-cost-collection/ accessed 17/03/2020.
15. Sterne JA, White IR, Carlin JB, et al. Multiple imputation for missing data in epidemiological and clinical research: potential and pitfalls. *BMJ* 2009;338:b2393. doi: 10.1136/bmj.b2393 [published Online First: 2009/07/01]
16. Nguyen CD, Carlin JB, Lee KJ. Model checking in multiple imputation: an overview and case study. *Emerg Themes Epidemiol* 2017;14:8. doi: 10.1186/s12982-017-0062-6 [published Online First: 2017/08/31]
17. Rubin DB. Multiple Imputation for Nonresponse in Surveys. United States: Wiley 2004.
18. R: A Language and Environment for Statistical Computing [program]. Vienna, Austria: R Foundation for Statistical Computing, 2020.
19. Dowle M, Srinivasan A. data.table: Extension of `data.frame`. R package version 1.13.2. https://CRAN.R-project.org/package=data.table. 2020
20. Wickham H, François R, Henry L, et al. dplyr: A Grammar of Data Manipulation. R package version 1.0.2. https://CRAN.R-project.org/package=dplyr. 2020
21. Wickham H. tidyr: Tidy Messy Data. R package version 1.1.2. https://CRAN.R-project.org/package=tidyr. 2020
22. naniar: Data Structures, Summaries, and Visualisations for Missing Data. R package version 0.6.0. https://CRAN.R-project.org/package=naniar [program], 2020.
23. Wickham H. Reshaping Data with the {reshape} Package. *Journal of Statistical Software* 2007;21(12):1-20.
24. Zeileis A, Grothendieck G. zoo: S3 Infrastructure for Regular and Irregular Time Series. *Journal of Statistical Software* 2005;14(6):1-27. doi: 10.18637/jss.v014.i06
25. Lüdecke D. sjmisc: Data and Variable Transformation Functions. *Journal of Open Source Software* 2018;3(26):754. doi: 10.21105/joss.00754
26. Morton F, Singh Nijjar J. eq5d: Methods for Calculating 'EQ-5D' Utility Index Scores. R package version 0.8.0. https://CRAN.R-project.org/package=eq5d. 2020
27. van Buuren S, Groothuis-Oudshoorn K. {mice}: Multivariate Imputation by Chained Equations in R. *Journal of Statistical Software* 2011;45(3):1-67.
28. Robitzsch A, Grund S. miceadds: Some Additional Multiple Imputation Functions, Especially for 'mice'. R package version 3.10-28. https://CRAN.R-project.org/package=miceadds. 2020

For peer review only

29. Bates D, Maechler M, Bolker B, et al. Fitting Linear Mixed-Effects Models Using {lme4}. *Journal of Statistical Software* 2015;67(1):1-48. doi: 10.18637/jss.v067.i01
30. JH. Z, Schafer JL. pan: Multiple imputation for multivariate panel or clustered dataR package version 1.6. 2018
31. Wickham H. ggplot2: Elegant Graphics for Data Analysis: Springer-Verlag New York 2016.
32. Wickham H. scales: Scale Functions for Visualization. R package version 1.1.1. https://CRAN.R-project.org/package=scales. 2020
33. Sievert C. Interactive Web-Based Data Visualization with R, plotly, and shiny: Chapman and Hall/CRC 2020.
34. von Elm E, Altman DG, Egger M, et al. The Strengthening the Reporting of Observational Studies in Epidemiology (STROBE) statement: guidelines for reporting observational studies. *PLoS Med* 2007;4(10):e296. doi: 10.1371/journal.pmed.0040296 [published Online First: 2007/10/19]
35. Dakin H, Gray A, Fitzpatrick R, et al. Rationing of total knee replacement: a cost-effectiveness analysis on a large trial data set. *BMJ Open* 2012;2(1):e000332. doi: 10.1136/bmjopen-2011-000332
36. NHS Digital. Health Gain for Knee Replacements. Finalised Patient Reported Outcome Measures (PROMs) in England for Hip & Knee Replacements, April 2018 – March 2019 2020 [Available from: https://digital.nhs.uk/data-and-information/publications/statistical/patient-reported-outcome-measures-proms/finalised-hip--knee-replacements-april-2018---march-2019/average-health-gain-copy accessed 15/12/2020.
37. Dakin H, Gray A, Murray D. Mapping analyses to estimate EQ-5D utilities and responses based on Oxford Knee Score. *Quality of life research : an international journal of quality of life aspects of treatment, care and rehabilitation* 2013;22(3):683-94. doi: 10.1007/s11136-012-0189-4 [published Online First: 2012/05/05]
38. Soni A, Wanigasekera V, Mezue M, et al. Central Sensitization in Knee Osteoarthritis: Relating Presurgical Brainstem Neuroimaging and PainDETECT-Based Patient Stratification to Arthroplasty Outcome. *Arthritis Rheumatol* 2019;71(4):550-60. doi: 10.1002/art.40749 [published Online First: 2018/10/09]
39. Brummett CM, Urquhart AG, Hassett AL, et al. Characteristics of fibromyalgia independently predict poorer long-term analgesic outcomes following total knee and hip arthroplasty. *Arthritis Rheumatol* 2015;67(5):1386-94. doi: 10.1002/art.39051 [published Online First: 2015/03/17]
40. Gwilym SE, Filippini N, Douaud G, et al. Thalamic atrophy associated with painful osteoarthritis of the hip is reversible after arthroplasty: a longitudinal voxel-based morphometric study. *Arthritis Rheum* 2010;62(10):2930-40. doi: 10.1002/art.27585 [published Online First: 2010/06/03]
41. Brander V, Gondek S, Martin E, et al. Pain and depression influence outcome 5 years after knee replacement surgery. *Clin Orthop Relat Res* 2007;464:21-6. doi: 10.1097/BLO.0b013e318126c032 [published Online First: 2007/07/03]
42. Soni A, Kiran A, Hart DJ, et al. Prevalence of reported knee pain over twelve years in a community-based cohort. *Arthritis Rheum* 2012;64(4):1145-52. doi: 10.1002/art.33434 [published Online First: 2011/12/20]
43. White IR, Royston P, Wood AM. Multiple imputation using chained equations: Issues and guidance for practice. *Stat Med* 2011;30(4):377-99. doi: 10.1002/sim.4067 [published Online First: 2011/01/13]
44. Kristman V, Manno M, Côté P. Loss to Follow-Up in Cohort Studies: How Much is Too Much? *European Journal of Epidemiology* 2004;19(8):751-60. doi: 10.1023/B:EJEP.0000036568.02655.f8

For peer review only

45. Cutler RB, Fishbain DA, Cole B, et al. Identifying patients at risk for loss to follow-up after pain center treatment. *Pain Med* 2001;2(1):46-51. doi: 10.1046/j.1526-4637.2001.002001046.x [published Online First: 2004/04/23]

FIGURE LEGENDS

Figure 1 – Progression of health utility by chronic pain (CP) status 1-year after total knee replacement surgery, defined using a threshold score in the Oxford Knee Score pain subscale

Figure 2 – Yearly healthcare costs by chronic pain (CP) status 1-year after total knee replacement surgery, defined using a threshold score in the Oxford Knee Score pain subscale

Figure 3 – Progression of chronic pain status over five years after total knee replacement (TKR), showing participants in chronic pain (dark grey) and not in chronic pain (light grey). Chronic pain status set using a threshold value for the mean Oxford Knee Score pain subscale value across 50 imputations for each participant at each year.

For peer review only

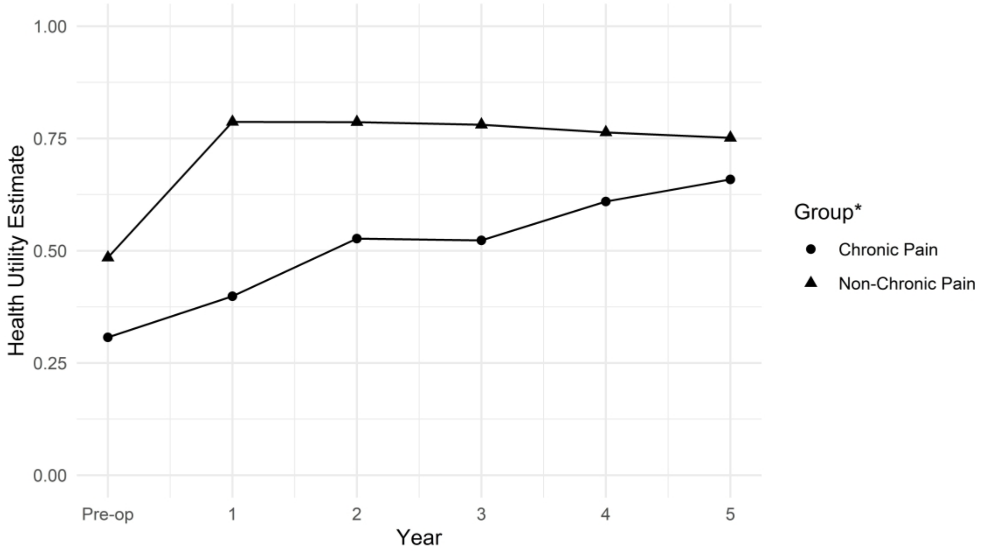

179x101mm (1200 x 1200 DPI)

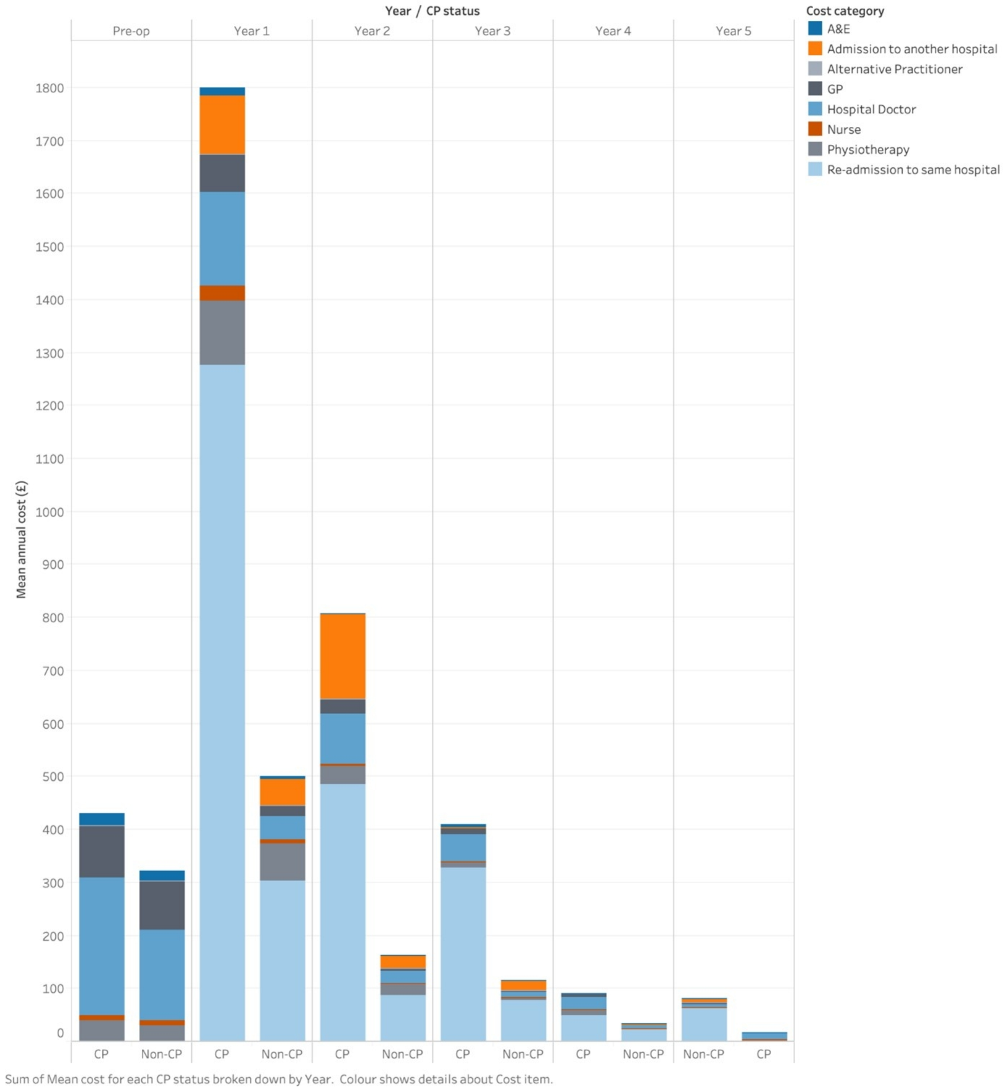

159x173mm (220 x 220 DPI)

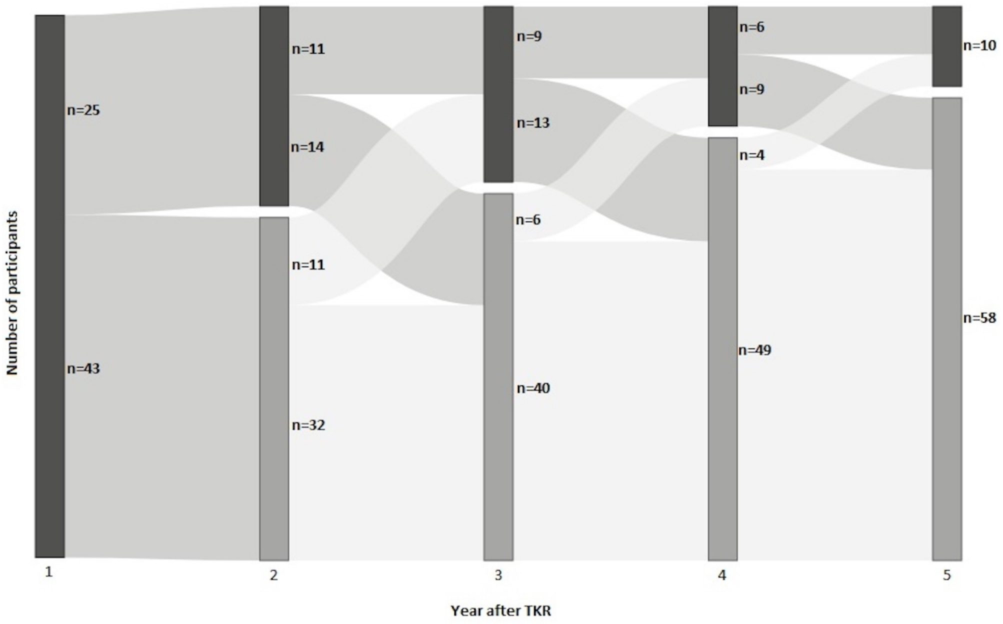

229x146mm (150 x 150 DPI)

ONLINE SUPPLEMENTAL MATERIAL

Tables

Table A.1: Observed and imputed health utility and Oxford Knee Score pain subscale (OKS-PS) scores per year

Observed						Imputed							
year	n	mean	SD	min	max	year	n	Pooled mean	Pooled SE	95% confidence interval		min	max
Health utility (EQ5D-3L) index													
pre-op	491	0.447	0.291	-0.239	1	pre-op	550	0.462	0.112	0.243	0.682	-0.349	1
1	531	0.742	0.253	-0.319	1	1	550	0.738	0.104	0.534	0.943	-0.319	1
2	447	0.765	0.265	-0.429	1	2	550	0.754	0.106	0.546	0.962	-0.429	1
3	403	0.761	0.253	-0.358	1	3	550	0.748	0.103	0.547	0.950	-0.358	1
4	344	0.759	0.263	-0.135	1	4	550	0.744	0.103	0.542	0.947	-0.239	1
5	299	0.751	0.272	-0.239	1	5	550	0.740	0.104	0.536	0.943	-0.239	1
OKS pain subscale score													
pre-op	488	10.09	4.59	0	26	pre-op	550	10.85	0.48	9.90	11.79	0	28
1	525	22.06	6.14	1	28	1	550	22.09	0.51	21.09	23.09	1	28
2	447	23.09	5.71	0	28	2	550	22.66	0.51	21.67	23.66	0	28
3	405	23.37	5.36	3	28	3	550	22.91	0.50	21.94	23.89	1	28
4	350	23.61	5.37	3	28	4	550	23.38	0.49	22.41	24.35	1	28
5	298	23.45	5.52	6	28	5	550	23.43	0.50	22.45	24.40	2	28

Pre-op: pre-operative; SD: standard deviation; SE: standard error

For peer review only

For peer review only - http://bmjopen.bmj.com/site/about/guidelines.xhtml

Table A.2: Year 5 study participant demographics

Variable	Observed OKS-PS at Year 5 (n=286)			Missing OKS-PS at Year 5 (n=264)		
**Age at total knee replacement (n, mean, mode)**						
	286	70	73	264	70	73
**Gender (n, %)**						
Female	159	55.6		155	58.7	
Male	127	44.4		109	41.3	
**IMD 2010 score (n, mean, mode)**						
	285	10.9	7.17	264	14.8	15.71
Missing (n (%))	1	(0.35)		0	(0.00)	
**BMI (n, mean, mode)**						
	284	30.3	26.79	262	31.0	30.9
Missing (n (%))	2	(0.70)		2	(0.76)	
**OKS at pre-operation (n, mean, (SD))**						
	251	20.1	(7.17)	233	18.4	(7.78)
Missing (n (%))	35	(12.24)		31	(11.74)	
**EQ5D at pre-operation (n, mean, (SD))**						
	252	0.4736	(0.276)	239	0.4180	(0.304)
Missing (n (%))	34	(11.89)		25	(9.47)	

For peer review only

Table A.3: Number of missing items during the 5 years of follow-up

	Total		Non-Chronic Pain		Chronic Pain		No OKS Category	
	n	%	n	%	n	%	n	%
**Number of years of missing OKS items**								
0	175	31.8	163	35.6	12	17.9	0	0
1	141	25.6	120	26.2	18	26.9	3	12
2	113	20.5	94	20.5	9	13.4	10	40
3	57	10.4	42	9.2	12	17.9	3	12
4	56	10.2	37	8.1	15	22.4	4	16
5	8	1.5	2	0.4	1	1.5	5	20
**Number of years of missing EQ5D items**								
0	219	39.8	201	43.9	14	20.9	4	16
1	116	21.1	95	20.7	14	20.9	7	28
2	100	18.2	81	17.7	14	20.9	5	20
3	56	10.2	41	9	13	19.4	2	8
4	53	9.6	37	8.1	11	16.4	5	20
5	6	1.1	3	0.7	1	1.5	2	8

For peer review only

Table A.4: Logistic regression p-values: preoperative variables associated with missingness - significant at a 5% level

	Missing OKS-PS				
	**Year 1**	**Year 2**	**Year 3**	**Year 4**	**Year 5**
**Age at operation**	0.227	0.790	0.906	0.483	0.537
**Gender**	0.058*	0.172	0.410	0.079*	0.475
**EQ5D dimension 1**	0.986	0.241	0.395	0.670	0.348
**EQ5D dimension 2**	**0.021**	**0.015**	**0.007**	**0.009**	**0.020**
**EQ5D dimension 3**	0.205	**0.010**	**0.037**	0.088*	0.211
**EQ5D dimension 4**	**0.025**	**0.008**	**0.025**	**0.002**	0.116
**EQ5D dimension 5**	0.474	**0.030**	0.133	0.404	0.101
**OKS Q1**	0.939	**0.026**	**0.024**	**0.016**	0.165
**OKS Q4**	0.052*	**0.000**	**0.003**	**0.000**	**0.003**
**OKS Q5**	0.361	0.138	0.173	**0.025**	0.633
**OKS Q6**	**0.039**	**0.016**	**0.021**	0.056*	0.997
**OKS Q8**	0.839	0.080*	0.424	**0.014**	0.641
**OKS Q9**	0.184	**0.004**	0.140	**0.001**	0.105
**OKS Q10**	0.086*	**0.000**	**0.000**	**0.001**	**0.006**

*significant at a 10% level only

For peer review only - http://bmjopen.bmj.com/site/about/guidelines.xhtml

Table A.5: Health utility scores per year by chronic pain group

	Pooled mean	Pooled SE	95% confidence interval	
**Chronic pain 1-year after total knee replacement**				
Pre-op	0.307	0.197	-0.079	0.694
Year 1	0.399	0.194	0.019	0.778
Year 2	0.527	0.193	0.148	0.906
Year 3	0.523	0.194	0.143	0.904
Year 4	0.610	0.186	0.245	0.974
Year 5	0.659	0.183	0.300	1.018
**Non-chronic pain 1-year after total knee replacement**				
Pre-op	0.485	0.114	0.262	0.708
Year 1	0.787	0.097	0.597	0.976
Year 2	0.786	0.105	0.581	0.991
Year 3	0.781	0.100	0.584	0.977
Year 4	0.764	0.104	0.559	0.968
Year 5	0.751	0.106	0.543	0.960

Pre-op: pre-operative; SE: standard error

For peer review only

Table A.6: Oxford Knee Score pain subscale (OKS-PS) scores per year by chronic pain group

	Pooled mean	Pooled SE	Confidence interval	
**Chronic pain 1-year after total knee replacement**				
Pre-op	8.04	0.79	6.50	9.59
Year 1	9.73	0.66	8.45	11.02
Year 2	16.06	0.98	14.15	17.98
Year 3	17.05	1.00	15.09	19.00
Year 4	19.17	1.01	17.19	21.15
Year 5	20.80	0.98	18.88	22.71
**Non-chronic pain 1-year after total knee replacement**				
Pre-op	11.25	0.50	10.27	12.22
Year 1	23.86	0.42	23.03	24.69
Year 2	23.61	0.49	22.65	24.56
Year 3	23.76	0.48	22.81	24.70
Year 4	23.98	0.48	23.03	24.93
Year 5	23.80	0.50	22.82	24.79

Pre-op: pre-operative; SE: standard error

For peer review only

For peer review only - http://bmjopen.bmj.com/site/about/guidelines.xhtml

Table A.7a: Primary care healthcare resource use by chronic pain (CP) group, defined 1-year after total knee replacement (Pre-op, Year 1 and Year 2)

	Pre-op		Year 1		Year 2	
	**CP (n=70)**	**Non-CP (n=482)**	**CP (n=70)**	**Non-CP (n=482)**	**CP (n=70)**	**Non-CP (n=482)**
**Saw a general practitioner (n (%))**						
Yes	58 (82.86)	390 (80.91)	44 (62.86)	135 (28.01)	24 (34.29)	46 (9.54)
No	8 (11.43)	43 (8.92)	23 (32.86)	335 (69.5)	22 (31.43)	361 (74.9)
Missing	*4 (5.71)*	*49 (10.17)*	*3 (4.29)*	*12 (2.49)*	*24 (34.29)*	*75 (15.56)*
Number of visits – last 12 months (mean (total))						
NHS	3.8 (209)	3.43 (1323)	2.66 (154)	0.64 (288)	1.29 (54)	0.21 (81)
Private	0 (0)	0.08 (4)	0.25 (6)	0.02 (6)	0 (0)	0 (0)
**Saw a physiotherapist**						
Yes	25 (35.71)	153 (31.74)	42 (60)	215 (44.61)	15 (21.43)	71 (14.73)
No	40 (57.14)	251 (52.07)	25 (35.71)	253 (52.49)	29 (41.43)	330 (68.46)
Missing	*5 (7.14)*	*78 (16.18)*	*3 (4.29)*	*14 (2.9)*	*26 (37.14)*	*81 (16.8)*
Number of visits – last 12 months (mean (total))						
NHS	1.35 (81)	1.12 (422)	4.08 (241)	2.28 (973)	1.65 (66)	0.81 (314)
Private	0.02 (1)	0.34 (92)	2.97 (86)	0.6 (167)	0 (0)	0.12 (39)
**Saw a nurse/practitioner**						
Yes	14 (20)	102 (21.16)	18 (25.71)	84 (17.43)	4 (5.71)	16 (3.32)
No	48 (68.57)	292 (60.58)	41 (58.57)	369 (76.56)	36 (51.43)	373 (77.39)
Missing	*8 (11.43)*	*88 (18.26)*	*11 (15.71)*	*29 (6.02)*	*30 (42.86)*	*93 (19.29)*
Number of visits – last 12 months (mean (total))						
NHS	0.7 (42)	0.72 (271)	2.34 (131)	0.47 (204)	0.43 (17)	0.08 (31)
Private	0 (0)	0 (0)	0 (0)	0 (0)	0 (0)	0 (1)
**Saw an alternative practitioner**						
Yes	3 (4.29)	41 (8.51)	7 (10)	19 (3.94)	4 (5.71)	5 (1.04)
No	61 (87.14)	386 (80.08)	62 (88.57)	456 (94.61)	42 (60)	406 (84.23)
Missing	*6 (8.57)*	*55 (11.41)*	*1 (1.43)*	*7 (1.45)*	*24 (34.29)*	*71 (14.73)*
Number of visits – last 12 months (mean (total))						
NHS	0.03 (2)	0.12 (46)	0 (0)	0.01 (3)	0.12 (5)	0 (0)
Private	0.13 (8)	0.32 (134)	0.32 (21)	0.11 (51)	0.2 (9)	0.11 (46)

For peer review only - http://bmjopen.bmj.com/site/about/guidelines.xhtml

Table A.7b: Primary care healthcare resource use by chronic pain (CP) group, defined 1-year after total knee replacement (Years 3-5)

	Year 3		Year 4		Year 5	
	**CP (n=70)**	**Non-CP (n=482)**	**CP (n=70)**	**Non-CP (n=482)**	**CP (n=70)**	**Non-CP (n=482)**
**Saw a general practitioner (n (%))**						
Yes	13 (18.57)	28 (5.81)	7 (10)	17 (3.53)	6 (8.57)	23 (4.77)
No	29 (41.43)	355 (73.65)	24 (34.29)	312 (64.73)	16 (22.86)	271 (56.22)
Missing	*28 (40)*	*99 (20.54)*	*39 (55.71)*	*153 (31.74)*	*48 (68.57)*	*188 (39)*
Number of visits – last 12 months (mean (total))						
NHS	0.69 (27)	0.16 (61)	0.63 (19)	0.11 (36)	0.33 (6)	0.13 (36)
Private	0 (0)	0 (0)	0 (0)	0 (0)	0.06 (1)	0 (0)
**Saw a physiotherapist**						
Yes	7 (10)	20 (4.15)	3 (4.29)	10 (2.07)	2 (2.86)	11 (2.28)
No	34 (48.57)	353 (73.24)	25 (35.71)	307 (63.69)	17 (24.29)	275 (57.05)
Missing	*29 (41.43)*	*109 (22.61)*	*42 (60)*	*165 (34.23)*	*51 (72.86)*	*196 (40.66)*
Number of visits – last 12 months (mean (total))						
NHS	29 (41.43)	355 (73.65)	24 (34.29)	312 (64.73)	16 (22.86)	271 (56.22)
Private	*28 (40)*	*99 (20.54)*	*39 (55.71)*	*153 (31.74)*	*48 (68.57)*	*188 (39)*
**Saw a nurse/practitioner**						
Yes	6 (8.57)	3 (0.62)	1 (1.43)	3 (0.62)	0 (0)	1 (0.21)
No	35 (50)	365 (75.73)	26 (37.14)	314 (65.15)	19 (27.14)	283 (58.71)
Missing	*29 (41.43)*	*114 (23.65)*	*43 (61.43)*	*165 (34.23)*	*51 (72.86)*	*198 (41.08)*
Number of visits – last 12 months (mean (total))						
NHS	0.16 (6)	0.02 (7)	0.15 (4)	0.02 (7)	0 (0)	0 (0)
Private	0 (0)	0 (0)	0 (0)	0 (0)	0 (0)	0 (0)
**Saw an alternative practitioner**						
Yes	6 (8.57)	6 (1.24)	2 (2.86)	7 (1.45)	1 (1.43)	3 (0.62)
No	37 (52.86)	374 (77.59)	28 (40)	322 (66.8)	20 (28.57)	290 (60.17)
Missing	*27 (38.57)*	*102 (21.16)*	*40 (57.14)*	*153 (31.74)*	*49 (70)*	*189 (39.21)*
Number of visits – last 12 months (mean (total))						
NHS	0.03 (1)	0.01 (5)	0 (0)	0.02 (6)	0 (0)	0 (0)
Private	0.48 (19)	0.11 (42)	0.07 (2)	0.05 (15)	0 (0)	0 (0)

For peer review only - http://bmjopen.bmj.com/site/about/guidelines.xhtml

For peer review only

Table A.8a: Hospital care resource use by chronic pain (CP) group, defined 1-year after total knee replacement (Pre-op, Year 1 and Year 2)

	Pre-op		Year 1		Year 2	
	**CP (n=70)**	**Non-CP (n=482)**	**CP (n=70)**	**Non-CP (n=482)**	**CP (n=70)**	**Non-CP (n=482)**
**Saw a hospital doctor**						
Yes	53 (75.71)	354 (73.44)	36 (51.43)	106 (21.99)	23 (32.86)	43 (8.92)
No	12 (17.14)	65 (13.49)	27 (38.57)	355 (73.65)	22 (31.43)	357 (74.07)
Missing	*5 (7.14)*	*63 (13.07)*	*7 (10)*	*21 (4.36)*	*25 (35.71)*	*82 (17.01)*
Number of visits – last 12 months (mean (total))						
NHS	2.52 (151)	1.8 (687)	1.72 (103)	0.4 (177)	1.3 (56)	0.22 (88)
Private	0.08 (1)	0.17 (13)	0 (0)	0 (1)	0 (0)	0 (0)
**Visited an accident and emergency department**						
Yes	9 (12.86)	51 (10.58)	6 (8.57)	15 (3.11)	1 (1.43)	5 (1.04)
No	55 (78.57)	373 (77.39)	62 (88.57)	462 (95.85)	44 (62.86)	403 (83.61)
Missing	*6 (8.57)*	*58 (12.03)*	*2 (2.86)*	*5 (1.04)*	*25 (35.71)*	*74 (15.35)*
Number of visits – last 12 months (mean (total))						
NHS	0.21 (13)	0.17 (72)	0.24 (16)	0.03 (12)	0.02 (1)	0.02 (7)
**Re-admitted to the (site-specific) hospital**						
Yes			15 (21.43)	29 (6.02)	6 (8.57)	8 (1.66)
No			54 (77.14)	447 (92.74)	40 (57.14)	409 (84.85)
Missing			*1 (1.43)*	*6 (1.24)*	*24 (34.29)*	*65 (13.49)*
Number of visits – last 12 months (mean (total))						
Days			1.07 (73)	0.25 (117)	0.59 (27)	0.04 (18)
**Admitted to any other hospital**						
Yes			1 (1.43)	6 (1.24)	2 (2.86)	2 (0.41)
No			67 (95.71)	470 (97.51)	45 (64.29)	412 (85.48)
Missing			*2 (2.86)*	*6 (1.24)*	*23 (32.86)*	*68 (14.11)*
Number of visits – last 12 months (mean (total))						
Days			0.1 (7)	0.04 (17)	0.43 (20)	0.01 (5)

For peer review only - http://bmjopen.bmj.com/site/about/guidelines.xhtml

Table A.8b: Hospital care resource use by chronic pain (CP) group, defined 1-year after total knee replacement (Years 3-5)

	**Year 3**		**Year 4**		**Year 5**	
	**CP (n=70)**	**Non-CP (n=482)**	**CP (n=70)**	**Non-CP (n=482)**	**CP (n=70)**	**Non-CP (n=482)**
**Saw a hospital doctor**						
Yes	14 (20)	19 (3.94)	7 (10)	10 (2.07)	6 (8.57)	16 (3.32)
No	31 (44.29)	355 (73.65)	24 (34.29)	311 (64.52)	16 (22.86)	274 (56.85)
Missing	*25 (35.71)*	*108 (22.41)*	*39 (55.71)*	*161 (33.4)*	*48 (68.57)*	*192 (39.83)*
Number of visits – last 12 months (mean (total))						
NHS	0.7 (30)	0.11 (41)	0.45 (13)	0.06 (20)	0.29 (6)	0.08 (23)
Private	0 (0)	0 (0)	0 (0)	0 (0)	0 (0)	0 (0)
**Visited an accident and emergency department**						
Yes	2 (2.86)	5 (1.04)	0 (0)	3 (0.62)	0 (0)	3 (0.62)
No	41 (58.57)	375 (77.8)	31 (44.29)	323 (67.01)	21 (30)	288 (59.75)
Missing	*27 (38.57)*	*102 (21.16)*	*39 (55.71)*	*156 (32.37)*	*49 (70)*	*191 (39.63)*
Number of visits – last 12 months (mean (total))						
NHS	0.09 (4)	0.01 (2)	0 (0)	0 (1)	0 (0)	0 (0)
**Re-admitted to the (site-specific) hospital**						
Yes	3 (4.29)	6 (1.24)	1 (1.43)	3 (0.62)	0 (0)	4 (0.83)
No	40 (57.14)	383 (79.46)	32 (45.71)	331 (68.67)	24 (34.29)	290 (60.17)
Missing	*27 (38.57)*	*93 (19.29)*	*37 (52.86)*	*148 (30.71)*	*46 (65.71)*	*188 (39)*
Number of visits – last 12 months (mean (total))						
Days	0.14 (6)	0.12 (46)	0.06 (2)	0.09 (31)	0 (0)	0.03 (8)
**Admitted to any other hospital**						
Yes	0 (0)	1 (0.21)	0 (0)	0 (0)	0 (0)	1 (0.21)
No	42 (60)	386 (80.08)	33 (47.14)	332 (68.88)	23 (32.86)	291 (60.37)
Missing	*28 (40)*	*95 (19.71)*	*37 (52.86)*	*150 (31.12)*	*47 (67.14)*	*190 (39.42)*
Number of visits – last 12 months (mean (total))						
Days	0 (0)	0 (1)	0 (0)	0 (0)	0 (0)	0 (1)

For peer review only

Table A.9a: Yearly healthcare costs by chronic pain (CP) (Pre-op, Year 1 and Year 2)

	CP at year 1 (n = 70)		Non-CP at year 1 (n = 482)	
	**Mean**	**(SD)**	**Mean**	**(SD)**
**Pre-operative - Healthcare professionals**				
General practitioner	98.53	(124.17)	90.58	(104.96)
Physiotherapist	40.50	(93.39)	30.64	(73.90)
Hospital doctor	258.86	(559.74)	171.04	(225.45)
Nurse	9.30	(27.60)	8.71	(29.74)
Alternative practitioner	1.00	(8.37)	3.34	(50.00)
**Pre-op - Hospital visits**				
Accident and emergency	21.60	(56.64)	17.78	(51.73)
Re-admitted to hospital				
Admitted to another hospital				
**Pre-op - Total**	**429.79**	**(611.41)**	**322.09**	**(337.22)**
**Year 1 - Healthcare professionals**				
General practitioner	72.60	(133.02)	19.72	(53.62)
Physiotherapist	120.50	(179.62)	70.65	(132.22)
Hospital doctor	176.57	(300.76)	44.07	(119.12)
Nurse	29.01	(118.95)	6.56	(24.31)
Alternative practitioner	0.00	(0.00)	0.22	(2.76)
**Year 1 - Hospital visits**				
Accident and emergency	14.40	(47.37)	5.23	(29.20)
Re-admitted to hospital	1276.42	(2647.66)	303.54	(1325.34)
Admitted to another hospital	109.47	(915.92)	51.14	(489.30)
**Year 1 - Total**	**1,798.97**	**(2981.69)**	**501.12**	**(1511.82)**
**Year 2 - Healthcare professionals**				
General practitioner	25.46	(48.06)	5.55	(24.42)
Physiotherapist	33.00	(95.13)	22.80	(73.23)
Hospital doctor	96.00	(220.61)	21.91	(109.69)
Nurse	3.76	(20.00)	1.00	(7.58)
Alternative practitioner	2.50	(20.92)	0.00	(0.00)
**Year 2 - Hospital visits**				
Accident and emergency	2.40	(20.08)	1.74	(17.04)
Re-admitted to hospital	486.42	(1825.23)	86.54	(791.78)
Admitted to another hospital	158.00	(996.48)	22.95	(381.51)
**Year 2 - Total**	**807.54**	**(2233.38)**	**162.48**	**(970.07)**

For peer review only - http://bmjopen.bmj.com/site/about/guidelines.xhtml

Table A.9b: Yearly healthcare costs by chronic pain (CP) (Years 3-5)

	CP at year 1 (n = 70)		Non-CP at year 1 (n = 482)	
	**Mean**	**(SD)**	**Mean**	**(SD)**
**Year 3 - Healthcare professionals**				
General practitioner	12.73	(36.97)	4.18	(24.51)
Physiotherapist	9.50	(46.89)	4.72	(28.11)
Hospital doctor	51.43	(136.40)	10.21	(64.63)
Nurse	1.33	(6.32)	0.23	(4.29)
Alternative practitioner	0.50	(4.18)	0.36	(7.97)
**Year 3 - Hospital visits**				
Accident and emergency	4.80	(28.19)	1.74	(17.04)
Re-admitted to hospital	328.42	(1563.26)	77.69	(728.20)
Admitted to another hospital	0.00	(0.00)	15.90	(349.05)
**Year 3 - Total**	**408.71**	**(1630.76)**	**115.02**	**(865.95)**
**Year 4 - Healthcare professionals**				
General practitioner	8.96	(33.20)	2.46	(21.45)
Physiotherapist	11.00	(57.94)	2.03	(22.70)
Hospital doctor	22.29	(89.74)	4.98	(42.79)
Nurse	0.89	(7.41)	0.23	(3.53)
Alternative practitioner	0.00	(0.00)	0.44	(8.13)
**Year 4 - Hospital visits**				
Accident and emergency	0.00	(0.00)	1.05	(13.23)
Re-admitted to hospital	48.53	(406.00)	22.95	(381.51)
Admitted to another hospital	0.00	(0.00)	0.00	(0.00)
**Year 4 - Total**	**91.66**	**(471.78)**	**34.13**	**(431.45)**
**Year 5 - Healthcare professionals**				
General practitioner	2.83	(17.54)	2.46	(17.63)
Physiotherapist	4.50	(27.90)	1.38	(14.12)
Hospital doctor	10.29	(39.53)	5.73	(43.74)
Nurse	0.00	(0.00)	0.00	(0.00)
Alternative practitioner	0.00	(0.00)	0.00	(0.00)
**Year 5 - Hospital visits**				
Accident and emergency	0.00	(0.00)	1.05	(13.23)
Re-admitted to hospital	0.00	(0.00)	63.59	(695.91)
Admitted to another hospital	0.00	(0.00)	7.05	(154.72)
**Year 5 - Total**	**17.61**	**(65.72)**	**81.26**	**(754.21)**

For peer review only - http://bmjopen.bmj.com/site/about/guidelines.xhtml

Table A.10: Mean costs for fluctuating and stable groups over chronic pain (CP) status between 1 and 2 years after total knee replacement – Year 1 CP

	Year 1 CP to Year 2 CP (n=25)				Year 1 CP to Year 2 Non-CP (n=19)			
	Year 1		Year 2		Year 1		Year 2	
	mean	SD	mean	SD	mean	SD	mean	SD
**Staff costs**								
General practitioner	52.8	82.50	42.2	49.06	78.2	188.36	33.0	65.08
Physiotherapist	96.6	159.84	60.2	135.37	71.8	89.97	38.7	88.00
Hospital doctor	201.6	399.08	158.4	253.90	113.7	176.52	138.9	283.59
Nurse	47.7	186.69	3.7	12.88	23.7	72.77	9.0	35.54
Alternative practitioner	0.0	0.00	0.0	0.00	0.0	0.00	9.2	40.15
**Hospital visit costs**								
Accident and emergency	13.4	46.52	0.0	0.00	8.8	38.54	8.8	38.54
Re-admitted to hospital	884.8	2244.93	748.9	2188.64	1210.0	2870.87	806.6	2416.20
Admitted to another hospital	306.5	1532.63	442.4	1650.37	0.0	0.00	0.0	0.00
**Total costs**	1603.5	2891.87	1455.9	2710.89	1506.2	3100.76	1044.3	2785.68

For peer review only - http://bmjopen.bmj.com/site/about/guidelines.xhtml

Table A.11: Mean costs for fluctuating and stable groups over chronic pain (CP) status between 1 and 2 years after total knee replacement – Year 1 Non-CP

	Year 1 Non-CP to Year 2 Non-CP (n=382)				Year 1 Non-CP to Year 2 CP (n=22)			
	Year 1		Year 2		Year 1		Year 2	
	mean	SD	mean	SD	mean	SD	mean	SD
**Staff costs**								
General practitioner	20.0	57.16	5.5	23.99	43.5	54.26	24.0	52.11
Physiotherapist	66.5	124.34	24.3	75.25	101.8	164.49	63.6	120.10
Hospital doctor	41.8	116.11	20.4	100.63	125.5	223.66	125.5	280.76
Nurse	6.2	25.23	0.9	7.52	8.5	18.97	2.8	10.30
Alternative practitioner	0.3	3.09	0.0	0.00	0.0	0.00	0.0	0.00
**Hospital visit costs**								
Accident and emergency	3.5	24.09	1.8	17.12	15.3	49.43	7.6	35.82
Re-admitted to hospital	307.3	1345.38	60.2	677.32	657.1	1855.51	851.1	2320.22
Admitted to another hospital	35.6	346.23	20.1	392.08	348.3	1633.78	154.4	724.22
**Total costs**	481.2	1488.25	133.2	835.38	1300.0	2512.02	1229.0	2755.09

For peer review only - http://bmjopen.bmj.com/site/about/guidelines.xhtml

Figure legends

Figure A. 1 Multiple imputation boxplot based on the mean health utility score across all 50 imputations for each participant

Figure A. 2: Multiple imputation histogram based on the mean Oxford Knee Score pain subscale (OKS-PS) across all 50 imputations for each participant

Figure A.3 Number of years of OKS-PS missing items by CP status

Figure A.4 Number of years of EQ-5D missing items by CP status

Figure A. 5: Oxford Knee Score pain subscale (OKS-PS) mean scores over the 5 years after total knee replacement surgery for those in chronic pain and those not in chronic pain 1-year after surgery (based on an OKS-PS threshold score).

For peer review only

For peer review only - http://bmjopen.bmj.com/site/about/guidelines.xhtml

Figures

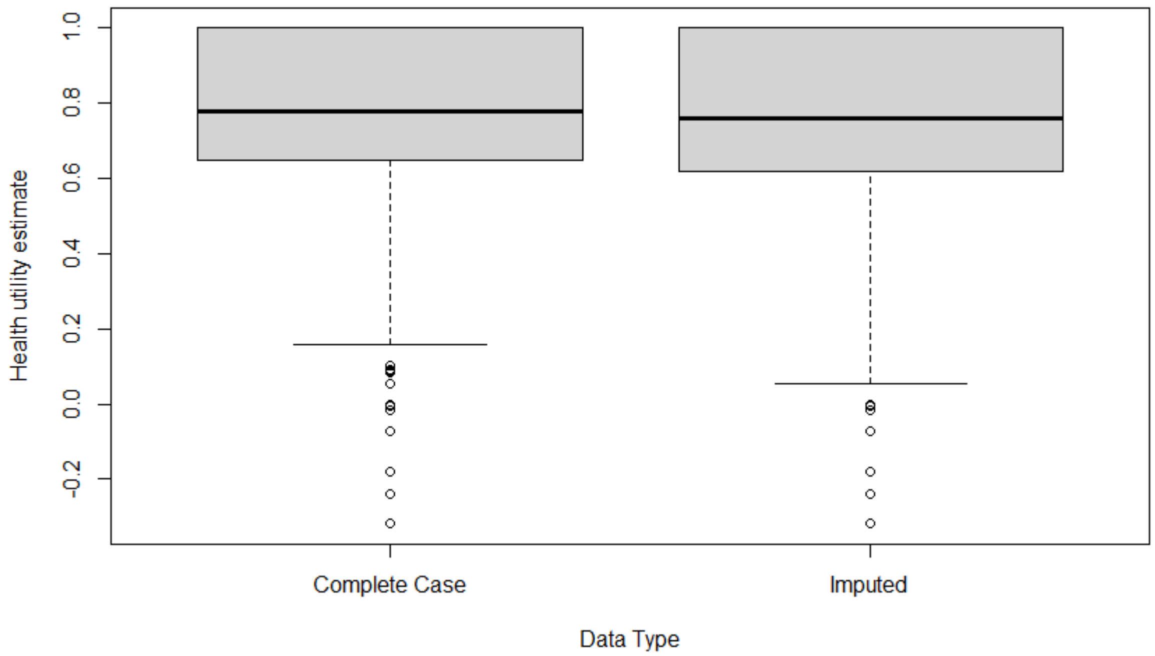

review only

For peer review only - http://bmjopen.bmj.com/site/about/guidelines.xhtml

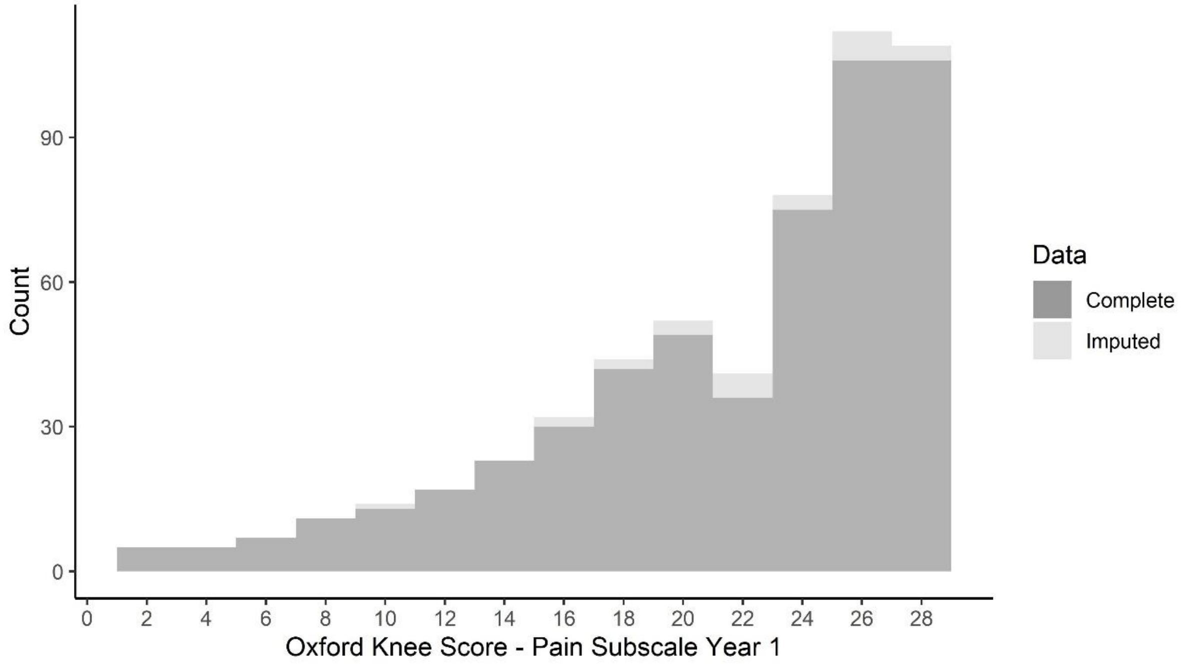

17

For peer review only - http://bmjopen.bmj.com/site/about/guidelines.xhtml

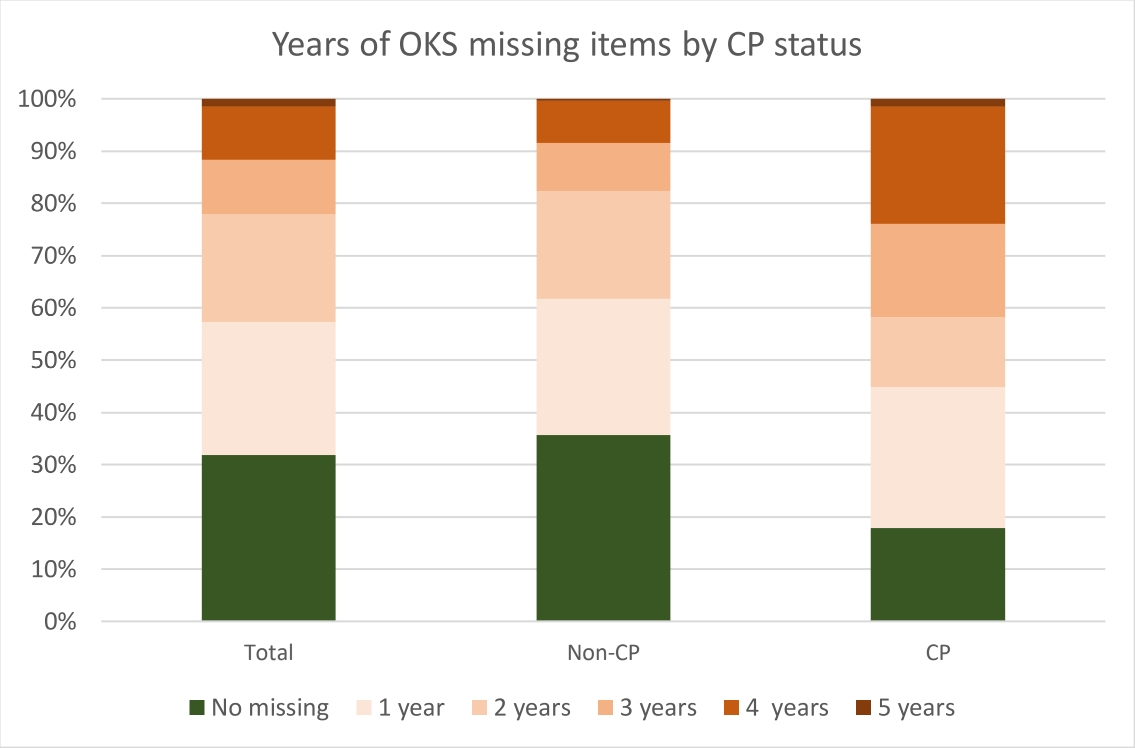

For peer review only - http://bmjopen.bmj.com/site/about/guidelines.xhtml

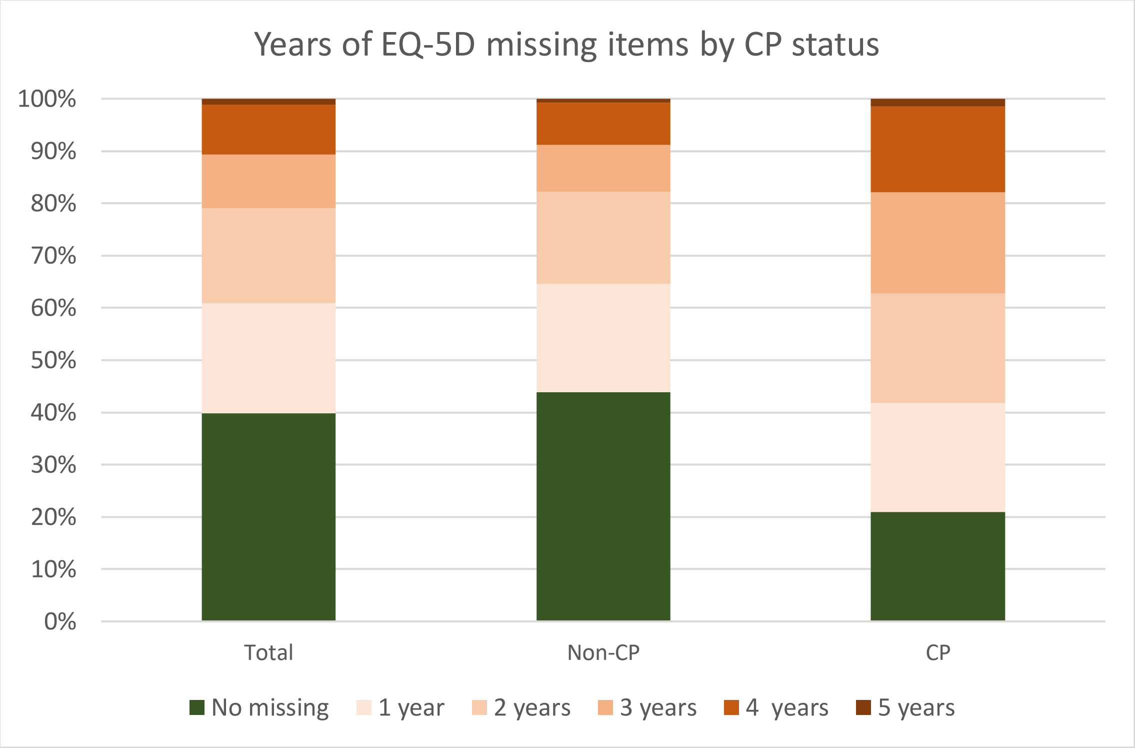

For peer review only

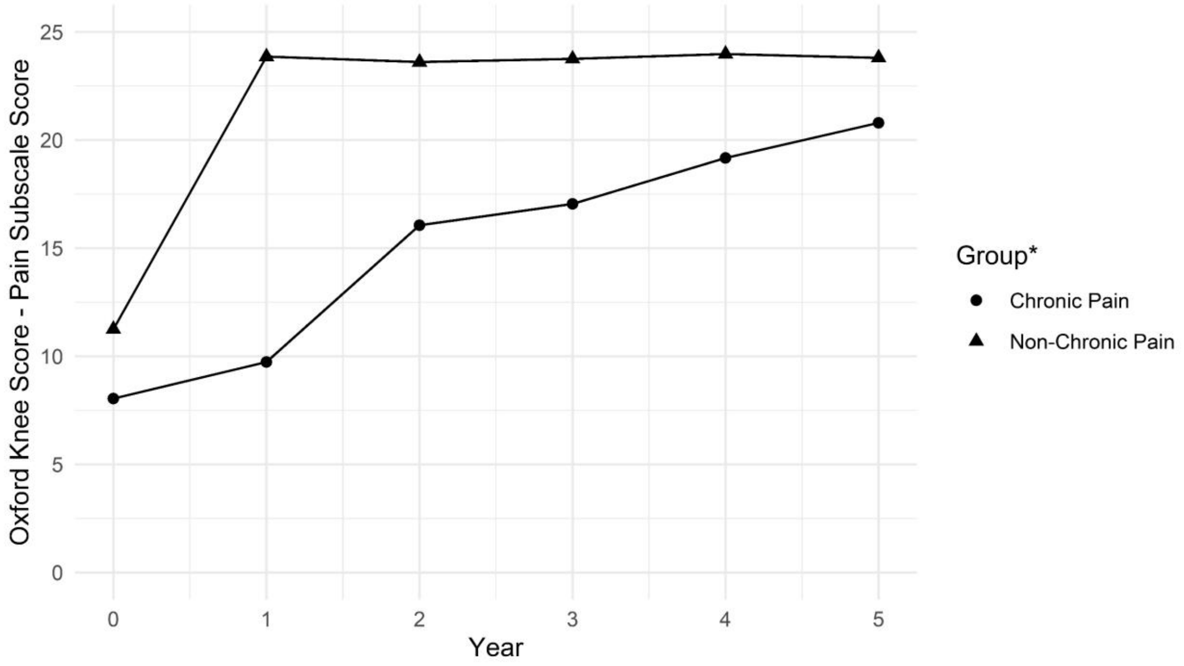

For peer review only

STROBE Statement—Checklist of items that should be included in reports of ***cohort studies***

	Item No	Recommendation	Page No
**Title and abstract**	1	(*a*) Indicate the study's design with a commonly used term in the title or the abstract<br>(*b*) Provide in the abstract an informative and balanced summary of what was done and what was found	1/3
**Introduction**			
Background/rationale	2	Explain the scientific background and rationale for the investigation being reported	4
Objectives	3	State specific objectives, including any prespecified hypotheses	5
**Methods**			
Study design	4	Present key elements of study design early in the paper	5
Setting	5	Describe the setting, locations, and relevant dates, including periods of recruitment, exposure, follow-up, and data collection	5
Participants	6	(*a*) Give the eligibility criteria, and the sources and methods of selection of participants. Describe methods of follow-up<br>(*b*) For matched studies, give matching criteria and number of exposed and unexposed	6
Variables	7	Clearly define all outcomes, exposures, predictors, potential confounders, and effect modifiers. Give diagnostic criteria, if applicable	7-8
Data sources/ measurement	8*	For each variable of interest, give sources of data and details of methods of assessment (measurement). Describe comparability of assessment methods if there is more than one group	5-8
Bias	9	Describe any efforts to address potential sources of bias	8
Study size	10	Explain how the study size was arrived at	6
Quantitative variables	11	Explain how quantitative variables were handled in the analyses. If applicable, describe which groupings were chosen and why	6-9
Statistical methods	12	(*a*) Describe all statistical methods, including those used to control for confounding<br>(*b*) Describe any methods used to examine subgroups and interactions<br>(*c*) Explain how missing data were addressed<br>(*d*) If applicable, explain how loss to follow-up was addressed<br>(*e*) Describe any sensitivity analyses	7-9
**Results**			
Participants	13*	(a) Report numbers of individuals at each stage of study—eg numbers potentially eligible, examined for eligibility, confirmed eligible, included in the study, completing follow-up, and analysed<br>(b) Give reasons for non-participation at each stage<br>(c) Consider use of a flow diagram	9-10
Descriptive data	14*	(a) Give characteristics of study participants (eg demographic, clinical, social) and information on exposures and potential confounders<br>(b) Indicate number of participants with missing data for each variable of interest<br>(c) Summarise follow-up time (eg, average and total amount)	10-12
Outcome data	15*	Report numbers of outcome events or summary measures over time	11-12

For peer review only - http://bmjopen.bmj.com/site/about/guidelines.xhtml

Main results	16	(*a*) Give unadjusted estimates and, if applicable, confounder-adjusted estimates and their precision (eg, 95% confidence interval). Make clear which confounders were adjusted for and why they were included<br>(*b*) Report category boundaries when continuous variables were categorized<br>(*c*) If relevant, consider translating estimates of relative risk into absolute risk for a meaningful time period	13-17
Other analyses	17	Report other analyses done—eg analyses of subgroups and interactions, and sensitivity analyses	
**Discussion**			
Key results	18	Summarise key results with reference to study objectives	17-19
Limitations	19	Discuss limitations of the study, taking into account sources of potential bias or imprecision. Discuss both direction and magnitude of any potential bias	19-21
Interpretation	20	Give a cautious overall interpretation of results considering objectives, limitations, multiplicity of analyses, results from similar studies, and other relevant evidence	22
Generalisability	21	Discuss the generalisability (external validity) of the study results	19-20
**Other information**			
Funding	22	Give the source of funding and the role of the funders for the present study and, if applicable, for the original study on which the present article is based	23-24

*Give information separately for exposed and unexposed groups.

Note: An Explanation and Elaboration article discusses each checklist item and gives methodological background and published examples of transparent reporting. The STROBE checklist is best used in conjunction with this article (freely available on the Web sites of PLoS Medicine at http://www.plosmedicine.org/, Annals of Internal Medicine at http://www.annals.org/, and Epidemiology at http://www.epidem.com/). Information on the STROBE Initiative is available at http://www.strobe-statement.org.

For peer review only

BMJ Open

Progression of chronic pain and associated health-related quality of life and healthcare resource use over 5 years after total knee replacement: evidence from a cohort study

Journal:	*BMJ Open*
Manuscript ID	bmjopen-2021-058044.R1
Article Type:	Original research
Date Submitted by the Author:	15-Mar-2022
Complete List of Authors:	Cole, Sophie; University of Oxford, Nuffield Department of Orthopaedics, Rheumatology and Musculoskeletal Sciences<br>Kolovos, Spyros; University of Oxford, Nuffield Department of Orthopaedics, Rheumatology and Musculoskeletal Sciences<br>Soni, Anushka; University of Oxford, Nuffield Department of Orthopaedics, Rheumatology and Musculoskeletal Sciences<br>Delmestri, Antonella; University of Oxford, Nuffield Department of Orthopaedics, Rheumatology and Musculoskeletal Sciences<br>Sanchez Santos, Maria ; University of Oxford, Nuffield Department of Orthopaedics, Rheumatology and Musculoskeletal Sciences<br>Judge, Andrew; University of Oxford, Nuffield Department of Orthopaedics, Rheumatology and Musculoskeletal Sciences; University of Bristol, National Institute for Health Research Bristol Biomedical Research Centre<br>Arden, Nigel; University of Oxford, Nuffield Department of Orthopaedics, Rheumatology and Musculoskeletal Sciences<br>Beswick, Andrew; University of Bristol, National Institute for Health Research Bristol Biomedical Research Centre; University of Bristol, Musculoskeletal Research Unit, Translational Health Sciences, Bristol Medical School<br>Wylde, Vikki; University of Bristol, National Institute for Health Research Bristol Biomedical Research Centre; University of Bristol, Musculoskeletal Research Unit, Translational Health Sciences, Bristol Medical School<br>Gooberman-Hill, Rachael; University of Bristol, National Institute for Health Research Bristol Biomedical Research Centre; University of Bristol, Musculoskeletal Research Unit, Translational Health Sciences, Bristol Medical School<br>Pinedo-Villanueva, Rafael; University of Oxford, Nuffield Department of Orthopaedics, Rheumatology and Musculoskeletal Sciences
**Primary Subject Heading**:	Health economics
Secondary Subject Heading:	Epidemiology, Health policy
Keywords:	HEALTH ECONOMICS, Health policy < HEALTH SERVICES ADMINISTRATION & MANAGEMENT, Quality in health care < HEALTH SERVICES ADMINISTRATION & MANAGEMENT, Knee < ORTHOPAEDIC & TRAUMA SURGERY, PAIN MANAGEMENT, EPIDEMIOLOGY

SCHOLARONE™
Manuscripts

I, the Submitting Author has the right to grant and does grant on behalf of all authors of the Work (as defined in the below author licence), an exclusive licence and/or a non-exclusive licence for contributions from authors who are: i) UK Crown employees; ii) where BMJ has agreed a CC-BY licence shall apply, and/or iii) in accordance with the terms applicable for US Federal Government officers or employees acting as part of their official duties; on a worldwide, perpetual, irrevocable, royalty-free basis to BMJ Publishing Group Ltd ("BMJ") its licensees and where the relevant Journal is co-owned by BMJ to the co-owners of the Journal, to publish the Work in this journal and any other BMJ products and to exploit all rights, as set out in our licence.

The Submitting Author accepts and understands that any supply made under these terms is made by BMJ to the Submitting Author unless you are acting as an employee on behalf of your employer or a postgraduate student of an affiliated institution which is paying any applicable article publishing charge ("APC") for Open Access articles. Where the Submitting Author wishes to make the Work available on an Open Access basis (and intends to pay the relevant APC), the terms of reuse of such Open Access shall be governed by a Creative Commons licence – details of these licences and which Creative Commons licence will apply to this Work are set out in our licence referred to above.

Other than as permitted in any relevant BMJ Author's Self Archiving Policies, I confirm this Work has not been accepted for publication elsewhere, is not being considered for publication elsewhere and does not duplicate material already published. I confirm all authors consent to publication of this Work and authorise the granting of this licence.

For peer review only

Progression of chronic pain and associated health-related quality of life and healthcare resource use over 5 years after total knee replacement: evidence from a cohort study

Sophie Cole, MSc[1], Spyros Kolovos, PhD[1], Anushka Soni, DPhil, BMBCh, MA(Hons)[1], Antonella Delmestri, PhD[1], Maria T Sanchez-Santos, MSc[1], Andrew Judge, PhD[1,2], Nigel Arden, MD[1], Andrew Beswick, BSc[2,3], Vikki Wylde, PhD[2,3], Rachael Gooberman-Hill, PhD[2,3], Rafael Pinedo-Villanueva, PhD[1]

Corresponding author: Rafael Pinedo-Villanueva[1]

Email: rafael.pinedo@ndorms.ox.ac.uk

Postal address: NDORMS, Botnar Research Centre, Old Road, Oxford, OX3 7LD

[1] Nuffield Department of Orthopaedics, Rheumatology and Musculoskeletal Sciences, Nuffield Orthopaedic Centre, University of Oxford, Windmill Road, Oxford, OX3 7LD, UK

[2] National Institute for Health Research Bristol Biomedical Research Centre, University Hospitals Bristol NHS Foundation Trust and University of Bristol, Bristol, UK

[3] Musculoskeletal Research Unit, Translational Health Sciences, Bristol Medical School, University of Bristol, Learning and Research Building, Level 1, Southmead Hospital, Bristol, BS10 5NB, UK

Word count: 4443

For peer review only

ARTICLE SUMMARY

Strengths and limitations of this study

- A strength of the study is that participant-level data were collected over 5 years after TKR enabling a detailed analysis of changes over time.
- The study cohort offered data on patient-reported outcome measures, allowing for a classification of participants according to their chronic pain status following surgery, and linked healthcare resource use to include in the analysis.
- This study followed pain trajectories of participants with post-surgical chronic pain which has not previously been explored.
- An important limitation was that many follow-up questionnaires were not returned, which generated an important level of missing data, addressed using standard methods of multiple imputation.
- The longitudinal study questionnaires did not ask participants about informal care, productivity losses, or their use of privately funded healthcare other than physiotherapists, which may play an important role in associated costs.

For peer review only

ABSTRACT

Objective: As part of the STAR Programme, a comprehensive study exploring long-term pain after surgery, we investigated how pain and function, health-related quality of life (HRQL), and healthcare resource use evolved over 5 years after total knee replacement (TKR) for those with and without chronic pain 1-year after their primary surgery.

Methods: We used data from the COASt prospective cohort study, which followed patients undergoing TKR from two English hospitals for 5 years. Chronic pain was defined using the Oxford Knee Score pain subscale (OKS-PS) where participants reporting a score of 14 or lower were classified as having chronic pain 1-year post-surgery . Pain and function were measured with the OKS, HRQL using the EQ-5D, resource use from yearly questionnaires, and costs estimated from a healthcare system perspective. We analysed the changes in OKS-PS, HRQL and resource use over a 5-year follow-up period. Multiple imputation accounted for missing data.

Results: Chronic pain was reported in 70/552 operated knees (12.7%) 1-year after surgery. The chronic pain group had worse pain, function, and HRQL pre- and post-surgery than the non-chronic pain group. Those without chronic pain markedly improved right after surgery, then plateaued. Those with chronic pain improved slowly but steadily. Participants with chronic pain reported greater healthcare resource use and costs than those without, especially 1-year after surgery, and mostly from hospital readmissions. 64.7% of those in chronic pain recovered during the following 4 years, while 30.9% fluctuated in and out of chronic pain.

Conclusion: Although TKR is often highly beneficial, some patients experienced chronic pain post-surgery. Although many fluctuated in their pain levels and most recovered over time,

For peer review only

identifying people most likely to have chronic pain and supporting their recovery would benefit patients and healthcare systems.

For peer review only

INTRODUCTION

Total knee replacement (TKR) provides pain relief and increases function for many people with advanced-stage knee osteoarthritis, which improves health-related quality of life (HRQL). However, a distinct, important group of people report long-term (chronic) pain after their TKR [1-5]. Chronic post-surgical pain is defined as pain that persists at least 3 months after surgery, beyond the healing process [6]. There is currently limited understanding of how chronic pain (CP) after a TKR changes over time, shapes people's lives, and affects HRQL and healthcare service use.

Although pain trajectories after TKR have previously been explored [7], those who report post-surgical CP have not been followed specifically. It's not yet known what proportion of individuals who experience CP in the first year after TKR find that their pain dissipates over the following few years, remain in CP, or find that their pain fluctuates. As CP is a complex construct, it would be useful to compare people who transition between these groups to understand whether they are more similar to the group they left or the group they joined. Better understanding of CP trajectories would help discern how CP evolves, giving patients clearer information about how likely they are to recover from their CP, and helping clinicians gain greater insight into the condition, hopefully contributing to finding ways to treat patients more effectively.

Assessing clinical and cost-effectiveness is a critical step in informing decision-making about the wider implementation of new interventions. For cost-effectiveness studies of potential new interventions for CP following surgery to be carried out, our understanding of the progression of CP must be accompanied by an assessment of HRQL and costs. HRQL is affected by a number of factors including the severity of pain and its consequences and is expected to vary as people's pain improves, worsens, or fluctuates. Changes in pain are also

For peer review only

likely to impact healthcare use, such as follow-up hospital visits, GP appointments, or prescriptions of analgesia. Healthcare resource use and costs after a TKR have been previously described [8 9] but to our knowledge these have not been examined specifically for those in CP, hence leaving an important research gap which needs to be closed to enable future cost-effectiveness assessments of interventions addressing CP after TKR. This study aimed to describe how pain and function, HRQL, and healthcare resource use evolved over the first five years after TKR, for patients with and without CP 1-year after surgery, using data collected in a published prospective cohort study [9]. A secondary objective was to map how the CP status of those with CP changed and assess how changes in HRQL and healthcare costs for those recovering from CP after the first year compared to those who did not recover or never had CP.

PATIENTS AND METHODS

COASt

The Clinical Outcomes in Arthroplasty Study (COASt), a prospective cohort study, tested outcome prediction models' performance [9] by collecting pre- and post-operative outcome data from patients undergoing hip and knee replacements with yearly follow-up. It was extended to 5 years to capture long-term pain and function, HRQL, and healthcare resource use.

COASt recruited patients receiving a joint replacement from the Nuffield Orthopaedic Centre in Oxford from 2010 and the Southampton General Hospital from 2011 (both in England). Patients were invited to participate after they were placed on the waiting list for a primary or revision knee replacement regardless of age, gender, BMI, or underlying cause for the surgery. Baseline information was collected in pre-operative questionnaires that

For peer review only

covered participant demographics such as the Index of Multiple Deprivation (IMD) (a measure of relative deprivation at a small local area level in England) and patient-reported outcome measures (PROMs): HRQL (EuroQuol EQ5D-3L) and pain and function (Oxford Knee Score, OKS). COASt participants were followed-up with yearly postal questionnaires for 5 years post-surgery.

We included COASt participants who underwent a primary TKR, returned their questionnaires 1-year after surgery, and completed the OKS pain subscale in that questionnaire for the resource use analysis. We excluded bilateral surgeries to be able to track levels of pain of a single operated knee. We did not have information about which patients had a revision surgery, which is highly unlikely during the first years after primary, or whether they had a contralateral operation. For the pain, function, and HRQoL analysis we also included participants who did not complete their OKS-PS at 1-year, in which cases missing data were imputed. The COASt study was approved by the Oxford Research Ethics Committee A (reference number 10/H0604/91). Patients listed for hip or knee replacement surgeries in the two participating centres (Southampton and Oxford) were potentially eligible for inclusion. Once identified, potential participants were sent a recruitment pack, consisting of a patient information sheet, a sample consent form, and a recruitment letter. They were later contacted by telephone by a member of the COASt team around two weeks after the pack was sent to discuss study details and answer any question they might have had. The COASt team member then took verbal consent if patients were satisfied and willing to participate in the study [9]. Research complied with the Helsinki Declaration.

Patient and Public Involvement

Patient and Public Involvement (PPI) members were actively involved in the design and recruitment of the STAR programme. PPI members participated in over 20 meetings of the

For peer review only

STAR Patient Forum to discuss and improve the participant experience. They gave feedback and recommended changes for both recruitment and study materials for the STAR trial, which was further informed by the results of this study. Findings were disseminated to study participants via regular programme bulletins and through STAR Patient Forum meetings.

Pain and function

Pain and functional ability were measured with the OKS, a patient-reported questionnaire consisting of 12 items assessing a patient's perspective of their joint pain and function after a TKR [10]. Each item has five possible responses, with higher values indicating better outcomes, and contributes 0-4 to the total score, which ranges 0-48.

The OKS pain subscale (OKS-PS) was used to identify those in CP, following work by some of our co-authors. The OKS-PS includes the 7 OKS questions relating to pain and is summarised by a score of 0-28 [11]. Participants reporting an OKS-PS of 14 or lower 1-year after surgery were classified as having CP, while those scoring above 14 were classified as non-CP [4]. We considered CP 1-year post-operatively to give patients sufficient time to recover from the operation and ensure that any pain reported was not due to the operation itself. While those classified as having no-CP may still experience some pain, they have been found to report much higher quality of life and greater satisfaction with the result of the operation[4].

Health-related quality of life

HRQL was measured using the EQ5D-3L questionnaire, which examines five dimensions of health: mobility, self-care, usual activities, pain/discomfort, and anxiety/depression. This instrument was completed at baseline (just before surgery) and annually after the TKR for 5 years. A health utility estimate, anchored at 0 representing death and with 1 representing full health, was calculated by applying a social preference tariff collected from a sample of the UK general population [12].

For peer review only

Healthcare resource use

Healthcare resource use was measured using participants' answers to questions about their visits to general practitioners, nurses, physiotherapists, alternative practitioners, and admissions to hospitals, because of their operated knee. These questions were included in the baseline and all follow-up questionnaires and covered the preceding 12 months.

The value of healthcare resources was estimated from the NHS perspective by applying mean unit costs sourced from the Personal Social Services Research Unit [13] and National Cost Collection [14]. To calculate the cost of readmittance to hospital, we used a binary variable and information from a free text section in the questionnaire explaining the readmittance. The submitted free text was used to identify participants who were treated for a knee infection or underwent a knee procedure. Unit costs were applied according to this categorisation and costs estimated by taking a weighted average across a number of reported knee procedures and treatments for knee infections [14].

Missing data

Loss to follow-up during the 5 years led to increasing amounts of missing data. Missing data and attrition are an important concern for longitudinal studies. It has been argued that the imputation of missing covariates data in medical research is always better than the complete case[15]. Excluding subjects with missing values leads to a reduction in the sample study size and may diminish the predictive power of the working statistical model. Therefore, multiple imputation assuming data missing at random was implemented to reduce the potential biases arising from missing data. Fifty imputed datasets were generated, with missing OKS-PS and EQ-5D values replaced by imputed ones [16]. We used the imputation by chain equation and applied the predictive mean matching two-level imputation method to account for each participant completing the follow-up questionnaires

For peer review only

multiple times. To ensure that imputation did not introduce bias into the results, we compared the outcome distribution in the observed and imputed datasets [17]. We also analysed the participant demographics for the observed and missing data at year 5 follow-up, how many years the participants had missing OKS-PS or EQ-5D values, and investigated our assumption of the missing data being missing at random with logistic regression. We censored participants who died within the 5 years of follow-up (n = 30) so that we did not impute data beyond their death. Summary statistics for the imputed dataset's outcomes were combined with Rubin's rule [18].

Analysis

We characterised HRQL and pain outcomes, resource use, and costs by CP group (CP and non-CP, based on 1-year post-operative outcomes) over 5 years by reporting the mean and standard deviation of the imputed OKS, OKS-PS, health utility, number of healthcare visits, and healthcare costs from pre-surgery to 5 years post-surgery. In addition, for OKS-PS and health utility we reported 95% confidence intervals. We considered pre-surgery as well as post-surgery to investigate whether those with CP post-surgery report distinct differences even prior to surgery.

To investigate the trajectory of CP, we tracked CP-group participants' movement in and out of CP and report the percentage that remained in CP for the entire period, recovered from CP at any point, and fluctuated between CP and non-CP over the 5 years. A participant's CP group status at each time point was determined by their observed OKS-PS, if available, or the mean across the 50 imputed scores, if missing. Transitions in and out of CP are illustrated in a Sankey diagram.

For peer review only

To investigate whether leaving the CP group after year 1 led to changes in HRQL or costs, we compared observed health utility and healthcare costs of CP-group participants who recovered from CP by year 2 after surgery and those who did not.

All analyses were conducted in R version 4.0.3[19] using multiple packages for data cleaning and statistical analysis [20-27], performing multiple imputation [28-31], and producing figures [32-34]. We followed the STROBE statement as a reporting guideline for this study [35].

For peer review only

RESULTS

Of the 1,025 knee procedures enrolled in COASt, all 580 corresponding to a TKR returned their year-1 follow-up questionnaire. We excluded 28 who did not report their OKS-PS in that questionnaire, giving a final sample of 552. Seventy (of 552, 12.7%) were classified as in CP 1-year after surgery using the OKS-PS. We refer to those classified as in CP 1-year after TKR as the CP group, and those not in CP 1-year after TKR as the non-CP group.

Table 1 shows that the CP and non-CP groups had similar demographics. Both had a mean age of 70 years at surgery and similar mean BMIs, although higher in the CP group. A greater proportion of the CP group were female (69%) than the non-CP group (54%). Both groups comprised a higher proportion of people living in less deprived areas than the national distribution. The CP group reported worse pre-operative pain, function, and HRQL scores than the non-CP group (Table 2). In addition, similar statistically significant differences were observed with linear regressions controlling for both gender and BMI. The amount of missing data due to loss to follow-up is reported in Tables 1 and 2.

Table 1: Study participants demographics

Table 1 – Study participant demographics

Variable		Total at Y1 (n = 552)			CP at Y1 (n = 70)			Non-CP at Y1 (n = 482)			P-value
**Age at total knee replacement**		N	Mean	Range	N	Mean	Range	N	Mean	Range	
Total		552	70	(38-90)	70	70	(42-88)	482	70	(38-90)	0.914
	Below 61	62	54	(38-60)	7	54	(42-58)	55	54	(38-60)	
	61-70	210	66	(61-70)	28	66	(61-70)	182	66	(61-70)	
	70-80	227	75	(71-80)	27	75	(71-79)	200	75	(71-80)	
	Above 80	53	84	(81-90)	8	84	(81-88)	45	84	(81-90)	
**Gender**		N	%		N	%		N	%		
Female		308	55.8		48	68.6		260	53.9		0.021
Male		244	44.2		22	31.4		222	46.1		
**IMD 2010 decile**		N	%		N	%		N	%		
1 (least deprived)		122	22.1		15	21.4		107	22.2		
2-5		294	53.3		33	47.1		261	54.2		0.187
6-9		130	23.6		20	28.5		110	22.8		
10 (most deprived)		5	0.9		2	2.9		3	0.6		
Missing (n, (%))		1	(0.2)		0	0		1	(0.2)		
**BMI**		N	Mean	SD	N	Mean	SD	N	Mean	SD	
Total		548	30.7	5.5	70	32.4	5.9	478	30.4	5.4	0.010
	Below 25	76	23.1	1.6	6	22.2	2.5	70	23.1	1.5	
	25 - 29.9	199	27.6	1.4	18	27.9	1.4	181	27.6	1.4	
	30 - 34.9	148	32.2	1.4	21	32.0	1.5	127	32.2	1.4	
	Above 34.9	125	38.5	3.4	25	38.4	3.9	100	38.5	3.3	
Missing (n, (%))		4	(0.7)		0	(0.00)		4	(0.8)		

BMI: body mass index; CP: chronic pain; IMD: Index of Multiple Deprivation; SD: standard deviation; Y: year
Differences in continuous and categorical variables between the CP and Non-CP groups were tested with a T-test and Chi-squared test, respectively.

For peer review only - http://bmjopen.bmj.com/site/about/guidelines.xhtml

Table 2: Study participants health outcomes

Table 2 – Study participants health outcomes

Variable		Total at Y1 (n=552)			CP at Y1 (n=70)			Non-CP at Y1 (n=482)			P-value
**OKS**		N	Mean	SD	N	Mean	SD	N	Mean	SD	
Baseline		490	19.4	7.7	62	14.1	6.8	428	20.2	7.5	<0.001
	Missing (n, (%))	62	(11.2%)		8	(11.4%)		54	(11.2%)		
Year 1		539	36.2	10.0	67	16.9	6.0	472	38.9	6.9	<0.001
	Missing (n, (%))	13	(2.4%)		3	(4.3%)		10	(2.1%)		
Year 2		435	37.8	9.5	44	22.1	8.7	391	39.6	7.8	<0.001
	Missing (n, (%))	117	(21.2%)		26	(37.1%)		91	(18.9%)		
Year 3		386	38.2	9.0	41	24.4	9.3	345	39.8	7.4	<0.001
	Missing (n, (%))	166	(30.1%)		29	(41.4%)		137	(28.4%)		
Year 4		320	38.6	8.9	30	26.0	10.4	290	39.9	7.6	<0.001
	Missing (n, (%))	232	(42.0%)		40	(57.1%)		192	(39.8%)		
Year 5		274	38.3	9.2	20	25.3	9.6	254	39.4	8.3	<0.001
	Missing (n, (%))	278	(50.4%)		50	(71.4%)		228	(47.3%)		
**OKS-PS**		N	Mean	SD	N	Mean	SD	N	Mean	SD	
Baseline		493	10.2	4.7	62	7.1	4.0	431	10.6	4.6	<0.001
	Missing (n, (%))	59	(10.7%)		8	(11.4%)		51	(10.6%)		
Year 1		552	22	6.1	70	9.7	3.6	482	23.8	4.0	<0.001
	Missing (n, (%))	0	(0.0%)		0	(0.0%)		0	(0.0%)		
Year 2		448	23.1	5.7	44	13.4	5.8	404	24.2	4.6	<0.001
	Missing (n, (%))	104	(18.8%)		26	(37.1%)		78	(16.2%)		
Year 3		402	23.3	5.5	41	14.8	6.1	361	24.3	4.5	<0.001
	Missing (n, (%))	150	(27.2%)		29	(41.4%)		121	(25.1%)		
Year 4		341	23.7	5.4	31	15.9	6.6	310	24.5	4.5	<0.001
	Missing (n, (%))	211	(38.2%)		39	(55.7%)		172	(35.7%)		
Year 5		286	23.5	5.5	21	15.7	5.9	265	24.1	5.0	<0.001
	Missing (n, (%))	266	(48.2%)		49	(70.0%)		217	(45.0%)		
**Health utility estimate**		N	Mean	SD	N	Mean	SD	N	Mean	SD	
Baseline		494	0.452	0.295	64	0.2732	0.315	430	0.4789	0.283	<0.001
	Missing (n, (%))	58	(10.5%)		6	(8.6%)		52	(10.8%)		
Year 1		538	0.74	0.254	67	0.3857	0.310	471	0.7909	0.199	<0.001
	Missing (n, (%))	14	(2.5%)		3	(4.3%)		11	(2.3%)		
Year 2		449	0.766	0.269	47	0.4422	0.321	402	0.804	0.235	<0.001
	Missing (n, (%))	103	(18.7%)		23	(32.9%)		80	(16.6%)		
Year 3		398	0.76	0.255	41	0.4209	0.314	357	0.7987	0.216	<0.001
	Missing (n, (%))	154	(27.9%)		29	(41.4%)		125	(25.9%)		
Year 4		336	0.761	0.265	31	0.4953	0.298	305	0.7877	0.247	<0.001
	Missing (n, (%))	216	(39.1%)		39	(55.7%)		177	(36.7%)		
Year 5		289	0.756	0.270	23	0.5398	0.326	266	0.7748	0.257	0.003
	Missing (n, (%))	263	(47.6%)		47	(67.1%)		216	(44.8%)		

CP: chronic pain; OKS: Oxford Knee Score; OKS-PS: Oxford Knee Score pain subscale; SD: standard deviation; Y: year

Differences in continuous variables between the CP and Non-CP groups were tested with a T-test

Characterisation of HRQL and pain outcomes, resource use, and costs

Observed and imputed data showed similar mean health utility and OKS-PS scores (Electronic Supplementary Material Table A.1 and Figures A.1 and A.2). Observed and missing data at year 5 of follow-up reported similar preoperative demographics and mean health utility and OKS-PS scores (Electronic Supplementary Material Table A.2). 77.9% of participants had between 0 and 2 missing years of OKS-PS across the 5 years of follow-up (Supplementary material Table A.3). Those in CP reported a higher number of years of missing items for both OKS-PS and EQ-5D (Electronic Supplementary Material Figures A.3 and A.4). Logistic regression showed that dimensions of EQ-5D and OKS-PS measured preoperatively had a statistically significant association with the missing OKS-PS across the 5 years of follow-up (Electronic Supplementary Material Table A.4).

The CP and non-CP groups had different mean health utility estimate scores before surgery (Figure 1) although their confidence intervals overlapped (Electronic Supplementary Material Table A.5). Participants in CP 1-year after TKR had a pre-operative health utility estimate of 0.307, whereas those who would not be in CP had a pre-operative score of 0.485. The two groups had noticeably different changes in health utility over the next 5 years. On average, the non-CP group improved significantly during the first year after surgery, reaching a score of 0.787 (compared to a pre-operative score of 0.485), and stayed at a similarly high level until follow-up ended (0.751 after 5 years). The CP group slowly improved their average health utility (except between years 2 and 3), going from 0.399 1-year after surgery to 0.656 5 years after surgery (Figure 1 and Electronic Supplementary Material Table A.5).

Figure 1: EQ5D-3L scores over time

For peer review only

There were similar patterns in pain progression, measured with the OKS-PS (Electronic Supplementary Material Figure A.5). The non-CP group started with a higher average pre-operative score (11.3), improved significantly during the first year after surgery (23.9), and then stabilised (23.8 after 5 years). The CP group started with a lower average pre-operative score (8.0) and slowly but steadily improved, from 9.7 after 1-year to 20.8 after 5 years (Electronic Supplementary Material Table A.6).

Tables A.7a-A.8b in the Electronic Supplementary Material show participants' use of healthcare resources. In the year before surgery, the groups made comparable numbers of visits to an NHS general practitioner (GP), with the CP group reporting an average 3.8 visits and the non-CP group an average 3.4. However, their use patterns differed after surgery. One year after surgery, mean yearly visits were 2.7 for the CP group and 0.6 for the non-CP group. The mean number of NHS GP visits then fell steadily for both groups to an average of 0.3 visits for the CP group and 0.1 for the non-CP group 5 years after surgery.

The groups' average annual number of visits to physiotherapists, hospital doctors, nurses, and alternative practitioners differed by healthcare specialist, but showed similar patterns. The CP and non-CP groups made similar numbers of visits before surgery; numbers surged for both groups during the 12 months after surgery, especially for the CP group. Visits then progressively decreased over the rest of the follow-up period for both groups. Although most physiotherapy visits were to NHS practitioners, during the first year post-surgery the CP group reported an average of 4 visits to NHS physiotherapists and 3 visits to private physiotherapists, whereas the non-CP group respectively made 2.2 and 0.6 visits. The groups' number of visits to Accident and Emergency, readmissions to the same hospital, and admission to any other hospital all showed similar patterns over time as the visits to non-GP healthcare professionals.

For peer review only

Participants in the two groups had similar mean healthcare costs in the year before surgery: £430 (SD = 611.41) for the CP group and £322 (SD = 337.22) for the non-CP group. Figure 2 shows the change in mean yearly costs by category over time. Consistent with the changes in resource use, mean costs increased to £1,799 (SD = 2981.69) for those in the CP group 1-year after TKR and £501 (SD = 1511.82) for those in the non-CP group. Readmission to hospital was responsible for 75% of the differences between the groups (£973 of £1,298) during this first year after surgery. After the first year, mean healthcare costs fell steadily for both cohorts. Electronic Supplementary Material Tables A.9a and A.9b show yearly healthcare costs by CP status from pre-operation to 5 years after TKR.

Figure 2 – Yearly healthcare costs by chronic pain (CP) status 1-year after total knee replacement surgery

Progression of chronic pain status

Figure 3 shows the change in CP status over the 5 years after TKR, based on yearly OKS-PS scores. Only 4.4% of those who reported experiencing CP after 1-year remained in CP throughout the 5 years. Almost a third of participants (30.9%) fluctuated in and out of CP. Most (64.7%) of those in CP 1-year post-surgery left CP within the next 4 years and did not experience CP again. Most of those who recovered from CP did so during the second year after surgery (26/44), with fewer people leaving CP each year thereafter except for the final year.

Figure 3: Sankey diagram of those in CP 1-year after surgery

Changes between year one and year two

Mean health utility remained stable for those who remained in the same chronic pain group between years 1 and 2, whilst those who moved into CP saw their health utility drop and those who recovered from it reported a clear improvement (Table 3).

For peer review only

Table 3: Mean health outcomes for fluctuating and stable groups over chronic pain (CP) status between 1 and 2 years after total knee replacement

Table 3: Mean health outcomes for fluctuating and stable groups over chronic pain (CP) status between 1 and 2 years after total knee replacement

	n	Mean	SD
**Health utility estimate**			
Year 1 CP to Year 2 CP			
Year 1	25	0.365	0.308
Year 2	25	0.326	0.328
Year 1 CP to Year 2 Non-CP			
Year 1	17	0.524	0.276
Year 2	19	0.619	0.216
Year 1 Non-CP to Year 2 Non-CP			
Year 1	374	0.811	0.189
Year 2	368	0.826	0.212
Year 1 Non-CP to Year 2 CP			
Year 1	21	0.604	0.175
Year 2	21	0.461	0.317
**OKS-PS**			
Year 1 CP to Year 2 CP			
Year 1	25	9.4	3.73
Year 2	25	9.4	3.93
Year 1 CP to Year 2 Non-CP			
Year 1	19	11.2	3.08
Year 2	19	18.7	2.75
Year 1 Non-CP to Year 2 Non-CP			
Year 1	382	24.3	3.63
Year 2	382	24.9	3.56
Year 1 Non-CP to Year 2 CP			
Year 1	22	17.2	2.48
Year 2	22	11.7	2.34

OKS-PS: Oxford Knee Score pain subscale; SD: standard deviation

For peer review only

The groups' healthcare costs followed similar patterns to their health utility scores (Electronic Supplementary Material Table A.10 and Table A.11). Participants who remained in CP had the highest costs in both the first (£1,600) and second (£1,450) years post-surgery. Those who recovered from CP after the first year reported a drop in costs from £1,500 in the first year to £1,000 in the second year. Those who developed CP in the second year saw their costs drop from £1,300 to £1,200. Those who remained free of CP had the lowest costs in both the first (£481) and second (£133) years.

DISCUSSION

Our analysis of data from COASt found that, on average, TKR led to large HRQL improvements, with health utility scores improving from 0.447 before surgery to 0.742 1-year after surgery. This improvement is consistent with what has been reported in previous studies [36], including average health utility gains of 0.334 over 6 months in 2018/2019 reported by the national English NHS PROMs [37]. However, these results were for all participants and hide the experience of those in CP for months after TKR. We found that 12.7% of participants reported CP 1-year after surgery, which is consistent with previous studies that used different mechanisms to ascertain CP [1-3 5].

Our characterisation of pain and function, HRQL, and healthcare resource use and costs by participants with and without CP 1-year after surgery suggests that these groups are distinct. However, confidence intervals are wide and overlap. Study participants in the non-CP group reported rapid, significant improvements in OKS-PS, OKS, and EQ-5D over the 5 years after surgery, whereas those with CP improved much less and much more slowly. It's not surprising that the pain, composite pain and function, and HRQL measures changed in similar ways, given their close association [38]. A key distinction between the groups was their

For peer review only

pre-operative health status. Those in the CP group had lower pre-operative scores, indicating worse health, than those without CP for pain (mean OKS-PS = 8 and 11, respectively) and HRQL (mean health utility = 0.307 and 0.485, respectively). Those in the CP group accessed more community healthcare and hospital services than those in the non-CP group at all time points, most notably during the first year after surgery (average healthcare costs: £1,800 for CP, £500 for non-CP). The main driver for the difference was readmission to the same hospital. Although the questionnaires used in COASt did not record the reason for readmissions, they were likely due to re-operations.

Two-thirds (64.7%) of participants with CP recovered and were no longer in CP within 5 years after surgery. This result suggests that CP after TKR is not a permanent condition and can improve with time as patients access routine care. There can be many reasons for these changes. They could be linked to individual characteristics, day-to-day life, or the severity of the original condition. Qualitative work from the STAR programme suggests that acceptance and self-management could potentially play a role, although this is an area that needs more research[39]. Another speculative hypothesis is that the fact that the CP group had more severe pain and reduced function prior to surgery may indicate that they were more deconditioned prior to surgery. This could explain the longer time to recovery, which would be supported by the fact that they had higher number of physiotherapy visits in the post-op period. Furthermore, it may be that some individuals learn to live with the pain overtime and choose self-management approaches[39 40].

Most of those who recovered from CP did so during the second year after surgery. One possible explanation for the prolonged recovery period from post-surgical pain may be in relation to the different pain mechanisms which are involved in OA [41]. Although the current study does not include any measures of centrally mediated pain, the presence of central

For peer review only

sensitisation has been previously demonstrated in patients with OA awaiting knee and hip arthroplasty and is also likely to be associated with worse outcome following arthroplasty [42-44]. . Whilst preliminary data suggest that some features of altered brain morphology associated with pain in osteoarthritis are potentially reversible within the first year after surgery [45], the nature and timing of any potential resolution of central sensitisation after surgery requires further investigation. Studies which have utilised questionnaire-based screening methods to identify features of central sensitisation in the pre and post-operative periods suggest it is likely to persist at least up to 2-years after surgery [46-49].

Only 4.4% of participants reported OKS-PS scores that indicated CP for all 5 years after surgery. In a 2007 study, Brander et al. [50] also found most patients with heightened, unexplained pain 1-year after knee replacement demonstrated subsequent improvement over several years. They found that depression was an important determinant of long-term outcomes.

A third (30.9%) of participants reported fluctuating OPS-PS scores that classified them as experiencing CP at only certain points during the study period. Relief from CP may not always be permanent, which is consistent with the variability in temporal fluctuation in knee pain seen without surgical intervention [51]. Although most participants eventually recovered from CP and made associated gains in HRQL, those who experienced CP 1-year after surgery did not reach the same level of health utility 5 years after surgery as without CP (0.659 vs 0.787, respectively). To our knowledge, this is the first study applying a CP criterion to examine whether and when people with CP after TKR recover.

Participants in CP after the first year after surgery who recovered by the second year improved their health utility more than those who stayed in CP, but not as much as those who were not in CP in either year. Improved OKS-PS is a sign of less pain. As it's likely

associated with improved mobility, self-care, and conduct of usual activities, it would be expected to lead to improved EQ-5D scores. However, participants switching CP groups also reported changes in use of healthcare resources that separated them from the CP group and brought them closer, albeit not directly in line with, participants without CP. These findings suggest progressive improvement as people come out of CP. The OKS-PS cut-off used to identify CP may therefore be sensitive enough to identify distinct groups as they transition between CP categories.

Our results suggest that under current clinical practice, many patients leave CP over time by gradually improving. Identifying these patients early will allow clinicians to provide them with tailored support to make that transition as quickly as possible. Better understanding which patients remain in CP or only recover after many years of struggling with pain will also help clinicians to design interventions to improve and manage these patients' pain, allow them to continue with their lives with the least interference possible.

This study has several potential limitations. As our analyses used data collected from patients recruited from two hospitals in Southampton and Oxford, the findings are not necessarily generalisable to the rest of the country. However, the participants' health gains after TKR agreed with those reported by the NHS PROMs programme[37], which invites all patients undergoing a knee replacement funded by the NHS in England to take part, suggesting a degree of generalisability. It is also possible that contralateral replacements and revisions, although unlikely, might have influenced the findings.

Our categorisation of CP was made using an OKS-PS cut-off score. As pain is a complex construct, it might not be appropriate to dichotomise it via a threshold on an instrument designed to measure improvement after knee replacement, rather than capturing the diverse dimensions of the pain experience. However, the use of this cut-off point to identify

For peer review only

people with and without CP effectively distinguished between groups with significantly different health outcomes (including HRQL), resource use, and healthcare costs [4], which can impact future guidelines for clinical care for people with CP. It should also be highlighted that chronic pain post-surgery will not be the only factor than influences the HRQL, healthcare resource use and costs of an individuals who has undergone a TKR and there may be additional causes of these changes.

The questionnaires used in COASt resulted in many study limitations, only some of which could be mitigated against. Many follow-up questionnaires were not returned, which generated an important level of missing data. It's difficult to know the direction of the impact this may have had as participants may not return questionnaires for multiple reasons, including both being very dissatisfied with their surgery or doing so well they could not be bothered to respond. We addressed this issue by applying multiple imputation to reduce bias and improve our study's power [17 52]. We also analysed the observed and missing data at 5-year follow-up and did not find a significant difference between the groups. Previous research has shown that assuming that missing data are either missing at random or missing completely at random, even with loss to follow-up of 60%, does not necessarily lead to significant bias of the results [53]. Research has also shown that loss to follow-up can be predicted by measures of pain and functioning, which supports our findings that dimensions of EQ-5D and OKS-PS are associated with missing data [54].

The questionnaires did not allow us to identify specific interventions received by participants after TKR, only unspecified hospital readmissions. We were therefore unable to identify whether the improvements observed were due to any particular interventions or treatments. Moreover, lacking specific intervention details was a limitation to estimate

For peer review only

costs to the NHS, but we addressed this by using average unit costs weighted by their relative use in the NHS [14].

As the resource-use questionnaires asked patients to report events that had occurred over the previous 12 months, they were potentially subject to recall bias. However, we are confident that most hospitalisations are sufficiently significant events for participants to have recalled them all. However, this may not be the case for less significant events such as a routine GP visit. Due to the potential bias from the self-reported resource use and the influence from factors not available for this study, the costs estimation should be used with caution and considered a broad estimate.

The questionnaires did not ask participants about informal care, productivity losses, or their use of privately funded healthcare other than physiotherapists. Although these issues are relevant for TKR patients, excluding this information was consistent with the perspective adopted for this analysis, i.e. that of the healthcare payer (NHS).

This study's main strength is the rich longitudinal dataset used. Unlike the national PROMs programme, which only collects PROMs before and 6 months after surgery, COASt collected data for 5 years after TKR. It also offered data on both PROMs and healthcare resource use, allowing us to explore the patterns in both.

Further research building on our findings is warranted. Identifying how best to assess pre- and post-surgical factors may help to provide more detailed understanding of variation in and potential predictors of CP and the likelihood and speed of recovery. We believe that including measures such as neuropathic pain, catastrophising, sleep disturbance, anxiety, depression, pain medication intake, self-prescription, and physical activity, among others, would help build a comprehensive, informative picture. In the meantime, clinicians can reassure patients that, though many fluctuate in their levels of pain, CP after TKR is

For peer review only

generally not permanent and that improvement over the five year after their primary is likely for many, if not most.

CONCLUSION

We identified a significant, rapid improvement in HRQL for people undergoing a TKR. This overall trend, however, appears to hide the slow and gradual improvement experienced by people with CP one year after their primary. They differed from those not in CP in terms of their progression of pain and function, HRQL, healthcare resource use, and costs. Our study suggests that the majority of people with CP after the first year eventually recovered by the fifth year after TKR, reducing their need for health care. Further research is needed to understand the reasons for this difference in progression as well as who is most likely to develop CP after TKR and how best to support patients in their long-term recovery, benefiting patients, their families, and healthcare systems.

For peer review only

CONTRIBUTORSHIP STATEMENT

RPV led the design of the work with substantial contributions from SC, SK, AS, and RGH. SC, SK, AS, AD, MSS, AJ, NA, AB, VW, RGH, and RPV contributed to the planning of the study. SC, SK, and RPV completed the data extraction criteria and AD curated and extracted the data. SC and SK undertook the data cleaning, SC and MSS conducted the multiple imputation, and SC did the analyses under the supervision of RPV. SC, SK, MSS, and RPV led the reporting of the methods and findings. RPV met with patients to gather PPI feedback. SC, SK, AS, AD, MSS, AJ, NA, AB, VW, RGH, and RPV contributed to the interpretation of data. SC and RPV wrote the original draft and SK, AS, AD, MSS, AJ, NA, AB, VW, and RGH made substantial contributions to subsequent versions and revised it critically for important intellectual content. SC, SK, AS, AD, MSS, AJ, NA, AB, VW, RGH, and RPV approved the final version of the manuscript and agree to be accountable for all aspects of the work by ensuring that questions related to the accuracy or integrity of any of its parts are appropriately investigated and resolved.

ACKNOWLEDGMENTS

We thank the participants of the COASt study in Southampton General Hospital and Nuffield Orthopaedic Centre in Oxford as well as all healthcare professionals and researchers involved. As a study conducted as part of the STAR programme, we acknowledge all members and affiliated members of the team: Aideen Ahern, Ashley Blom, Amanda Burston, Jane Dennis, Kirsty Garfield, Athene Lane, Fiona MacKichan, Sian Noble, Andrew Moore, Tim Peters, Emily Sanderson, Joanne Simon, Jodi Taylor, Paul Dieppe, Chris Eccleston, Nick Ambler, Wendy Bertram, Susan Bridgewater, Nick Howells, Leigh Morrison, Gemma

For peer review only

Munkenbeck, Candida McCabe, Andrew Toms, Rowenna Stroud, Kate Button, Simon White, Andrew Price, David Walsh, Julie Bruce, Stewart Long, Joanne Adams, Ben Burston, Vikran Desai, Tim Board, Colin Esler, Michael Parry, and the Patient Experience Partnership in Research (PEP-R) at the University of Bristol.

FUNDING

The STAR Programme was funded by the National Institute for Health Research (NIHR) [Programme Grant for Applied Research (Grant Reference Number RP-PG-0613-20001)] and supported by the NIHR Biomedical Research Centre at University Hospitals Bristol and Weston NHS Foundation Trust and the University of Bristol. The views expressed are those of the authors and not necessarily those of the NIHR or the Department of Health and Social Care. We acknowledge English language editing by Dr Jennifer A. de Beyer of the Centre for Statistics in Medicine, University of Oxford.

COMPETING INTERESTS

Prof Arden reports consultancy fees outside the scope of this work. All other authors report no competing interests.

DATA SHARING STATEMENT

No additional data available.

REFERENCES

1. Beswick AD, Wylde V, Gooberman-Hill R, et al. What proportion of patients report long-term pain after total hip or knee replacement for osteoarthritis? A systematic review of prospective

For peer review only

studies in unselected patients. *BMJ Open* 2012;2(1):e000435. doi: 10.1136/bmjopen-2011-000435
2. Rice DA, Kluger MT, McNair PJ, et al. Persistent postoperative pain after total knee arthroplasty: a prospective cohort study of potential risk factors. *Br J Anaesth* 2018;121(4):804-12. doi: 10.1016/j.bja.2018.05.070 [published Online First: 2018/09/22]
3. Gungor S, Fields K, Aiyer R, et al. Incidence and risk factors for development of persistent postsurgical pain following total knee arthroplasty: A retrospective cohort study. *Medicine (Baltimore)* 2019;98(28):e16450. doi: 10.1097/md.0000000000016450 [published Online First: 2019/07/16]
4. Pinedo-Villanueva R, Khalid S, Wylde V, et al. Identifying individuals with chronic pain after knee replacement: a population-cohort, cluster-analysis of Oxford knee scores in 128,145 patients from the English National Health Service. *BMC Musculoskelet Disord* 2018;19(1):354. doi: 10.1186/s12891-018-2270-9 [published Online First: 2018/10/05]
5. Vina ER, Hannon MJ, Kwoh CK. Improvement following total knee replacement surgery: Exploring preoperative symptoms and change in preoperative symptoms. *Semin Arthritis Rheum* 2016;45(5):547-55. doi: 10.1016/j.semarthrit.2015.10.002 [published Online First: 2015/11/18]
6. Schug SA, Lavand'homme P, Barke A, et al. The IASP classification of chronic pain for ICD-11: chronic postsurgical or posttraumatic pain. *Pain* 2019;160(1):45-52. doi: 10.1097/j.pain.0000000000001413 [published Online First: 2018/12/27]
7. Lenguerrand E, Wylde V, Gooberman-Hill R, et al. Trajectories of Pain and Function after Primary Hip and Knee Arthroplasty: The ADAPT Cohort Study. *PLoS One* 2016;11(2):e0149306. doi: 10.1371/journal.pone.0149306 [published Online First: 2016/02/13]
8. Bozic KJ, Stacey B, Berger A, et al. Resource utilization and costs before and after total joint arthroplasty. *BMC Health Serv Res* 2012;12:73. doi: 10.1186/1472-6963-12-73 [published Online First: 2012/03/27]
9. Arden N, Altman D, Beard D, et al. Lower limb arthroplasty: can we produce a tool to predict outcome and failure, and is it cost-effective? An epidemiological study. Southampton (UK): NIHR Journals Library 2017.
10. Dawson J, Fitzpatrick R, Murray D, et al. Questionnaire on the perceptions of patients about total knee replacement. *J Bone Joint Surg Br* 1998;80(1):63-9. doi: 10.1302/0301-620x.80b1.7859 [published Online First: 1998/02/14]
11. Harris K, Dawson J, Doll H, et al. Can pain and function be distinguished in the Oxford Knee Score in a meaningful way? An exploratory and confirmatory factor analysis. *Quality of life research : an international journal of quality of life aspects of treatment, care and rehabilitation* 2013;22(9):2561-8. doi: 10.1007/s11136-013-0393-x [published Online First: 2013/03/26]
12. Dolan P. Modeling valuations for EuroQol health states. *Med Care* 1997;35(11):1095-108. doi: 10.1097/00005650-199711000-00002 [published Online First: 1997/11/21]
13. Curtis L, Burns A. Unit Costs of Health and Social Care 2018, Personal Social Services Research Unit. University of Kent, Canterbury, 2018.
14. NHS. 2018/19 National Cost Collection data - National schedule of NHS costs 2020 [Available from: https://improvement.nhs.uk/resources/national-cost-collection/ accessed 17/03/2020.
15. Janssen KJ, Donders AR, Harrell FE, Jr., et al. Missing covariate data in medical research: to impute is better than to ignore. *J Clin Epidemiol* 2010;63(7):721-7. doi: 10.1016/j.jclinepi.2009.12.008 [published Online First: 20100324]
16. Sterne JA, White IR, Carlin JB, et al. Multiple imputation for missing data in epidemiological and clinical research: potential and pitfalls. *BMJ* 2009;338:b2393. doi: 10.1136/bmj.b2393 [published Online First: 2009/07/01]

For peer review only

17. Nguyen CD, Carlin JB, Lee KJ. Model checking in multiple imputation: an overview and case study. *Emerg Themes Epidemiol* 2017;14:8. doi: 10.1186/s12982-017-0062-6 [published Online First: 2017/08/31]
18. Rubin DB. Multiple Imputation for Nonresponse in Surveys. United States: Wiley 2004.
19. R: A Language and Environment for Statistical Computing [program]. Vienna, Austria: R Foundation for Statistical Computing, 2020.
20. Dowle M, Srinivasan A. data.table: Extension of `data.frame`. R package version 1.13.2. https://CRAN.R-project.org/package=data.table. 2020
21. Wickham H, François R, Henry L, et al. dplyr: A Grammar of Data Manipulation. R package version 1.0.2. https://CRAN.R-project.org/package=dplyr. 2020
22. Wickham H. tidyr: Tidy Messy Data. R package version 1.1.2. https://CRAN.R-project.org/package=tidyr. 2020
23. naniar: Data Structures, Summaries, and Visualisations for Missing Data. R package version 0.6.0. https://CRAN.R-project.org/package=naniar [program], 2020.
24. Wickham H. Reshaping Data with the {reshape} Package. *Journal of Statistical Software* 2007;21(12):1-20.
25. Zeileis A, Grothendieck G. zoo: S3 Infrastructure for Regular and Irregular Time Series. *Journal of Statistical Software* 2005;14(6):1-27. doi: 10.18637/jss.v014.i06
26. Lüdecke D. sjmisc: Data and Variable Transformation Functions. *Journal of Open Source Software* 2018;3(26):754. doi: 10.21105/joss.00754
27. Morton F, Singh Nijjar J. eq5d: Methods for Calculating 'EQ-5D' Utility Index Scores. R package version 0.8.0. https://CRAN.R-project.org/package=eq5d. 2020
28. van Buuren S, Groothuis-Oudshoorn K. {mice}: Multivariate Imputation by Chained Equations in R. *Journal of Statistical Software* 2011;45(3):1-67.
29. Robitzsch A, Grund S. miceadds: Some Additional Multiple Imputation Functions, Especially for 'mice'. R package version 3.10-28. https://CRAN.R-project.org/package=miceadds. 2020
30. Bates D, Maechler M, Bolker B, et al. Fitting Linear Mixed-Effects Models Using {lme4}. *Journal of Statistical Software* 2015;67(1):1-48. doi: 10.18637/jss.v067.i01
31. JH. Z, Schafer JL. pan: Multiple imputation for multivariate panel or clustered dataR package version 1.6. 2018
32. Wickham H. ggplot2: Elegant Graphics for Data Analysis: Springer-Verlag New York 2016.
33. Wickham H. scales: Scale Functions for Visualization. R package version 1.1.1. https://CRAN.R-project.org/package=scales. 2020
34. Sievert C. Interactive Web-Based Data Visualization with R, plotly, and shiny: Chapman and Hall/CRC 2020.
35. von Elm E, Altman DG, Egger M, et al. The Strengthening the Reporting of Observational Studies in Epidemiology (STROBE) statement: guidelines for reporting observational studies. *PLoS Med* 2007;4(10):e296. doi: 10.1371/journal.pmed.0040296 [published Online First: 2007/10/19]
36. Dakin H, Gray A, Fitzpatrick R, et al. Rationing of total knee replacement: a cost-effectiveness analysis on a large trial data set. *BMJ Open* 2012;2(1):e000332. doi: 10.1136/bmjopen-2011-000332
37. NHS Digital. Health Gain for Knee Replacements. Finalised Patient Reported Outcome Measures (PROMs) in England for Hip & Knee Replacements, April 2018 – March 2019 2020 [Available from: https://digital.nhs.uk/data-and-information/publications/statistical/patient-reported-outcome-measures-proms/finalised-hip--knee-replacements-april-2018---march-2019/average-health-gain-copy accessed 15/12/2020.
38. Dakin H, Gray A, Murray D. Mapping analyses to estimate EQ-5D utilities and responses based on Oxford Knee Score. *Quality of life research : an international journal of quality of life aspects of treatment, care and rehabilitation* 2013;22(3):683-94. doi: 10.1007/s11136-012-0189-4 [published Online First: 2012/05/05]

For peer review only

39. Moore AJ, Gooberman-Hill R. Why don't patients seek help for chronic post-surgical pain after knee replacement? A qualitative investigation. *Health Expect* 2020;23(5):1202-12. doi: 10.1111/hex.13098 [published Online First: 20200709]
40. Jeffery AE, Wylde V, Blom AW, et al. “It's there and I'm stuck with it”: Patients' experiences of chronic pain following total knee replacement surgery. *Arthritis Care & Research* 2011;63(2):286-92. doi: https://doi.org/10.1002/acr.20360
41. Cohen E, Lee YC. A Mechanism-Based Approach to the Management of Osteoarthritis Pain. *Curr Osteoporos Rep* 2015;13(6):399-406. doi: 10.1007/s11914-015-0291-y
42. Gwilym SE, Keltner JR, Warnaby CE, et al. Psychophysical and functional imaging evidence supporting the presence of central sensitization in a cohort of osteoarthritis patients. *Arthritis Rheum* 2009;61(9):1226-34. doi: 10.1002/art.24837
43. Soni A, Wanigasekera V, Mezue M, et al. Central Sensitization in Knee Osteoarthritis: Relating Presurgical Brainstem Neuroimaging and PainDETECT-Based Patient Stratification to Arthroplasty Outcome. *Arthritis Rheumatol* 2019;71(4):550-60. doi: 10.1002/art.40749 [published Online First: 2018/10/09]
44. Brummett CM, Urquhart AG, Hassett AL, et al. Characteristics of fibromyalgia independently predict poorer long-term analgesic outcomes following total knee and hip arthroplasty. *Arthritis Rheumatol* 2015;67(5):1386-94. doi: 10.1002/art.39051 [published Online First: 2015/03/17]
45. Gwilym SE, Filippini N, Douaud G, et al. Thalamic atrophy associated with painful osteoarthritis of the hip is reversible after arthroplasty: a longitudinal voxel-based morphometric study. *Arthritis Rheum* 2010;62(10):2930-40. doi: 10.1002/art.27585 [published Online First: 2010/06/03]
46. Koh IJ, Kang BM, Kim MS, et al. How Does Preoperative Central Sensitization Affect Quality of Life Following Total Knee Arthroplasty? *J Arthroplasty* 2020;35(8):2044-49. doi: 10.1016/j.arth.2020.04.004 [published Online First: 20200410]
47. Casado-Adam P, Jiménez-Vílchez AJ, Güler-Caamaño I, et al. [Pain evolution in patients with central sensitization and osteoarthritis after knee arthroplasty]. *Rehabilitacion (Madr)* 2022;56(1):47-55. doi: 10.1016/j.rh.2021.06.002 [published Online First: 20210711]
48. Ohashi Y, Fukushima K, Uchida K, et al. Adverse Effects of Higher Preoperative Pain at Rest, a Central Sensitization-Related Symptom, on Outcomes After Total Hip Arthroplasty in Patients with Osteoarthritis. *J Pain Res* 2021;14:3345-52. doi: 10.2147/jpr.S322314 [published Online First: 20211020]
49. Kim MS, Koh IJ, Sohn S, et al. Central Sensitization Is a Risk Factor for Persistent Postoperative Pain and Dissatisfaction in Patients Undergoing Revision Total Knee Arthroplasty. *J Arthroplasty* 2019;34(8):1740-48. doi: 10.1016/j.arth.2019.03.042 [published Online First: 20190328]
50. Brander V, Gondek S, Martin E, et al. Pain and depression influence outcome 5 years after knee replacement surgery. *Clin Orthop Relat Res* 2007;464:21-6. doi: 10.1097/BLO.0b013e318126c032 [published Online First: 2007/07/03]
51. Soni A, Kiran A, Hart DJ, et al. Prevalence of reported knee pain over twelve years in a community-based cohort. *Arthritis Rheum* 2012;64(4):1145-52. doi: 10.1002/art.33434 [published Online First: 2011/12/20]
52. White IR, Royston P, Wood AM. Multiple imputation using chained equations: Issues and guidance for practice. *Stat Med* 2011;30(4):377-99. doi: 10.1002/sim.4067 [published Online First: 2011/01/13]
53. Kristman V, Manno M, Côté P. Loss to Follow-Up in Cohort Studies: How Much is Too Much? *European Journal of Epidemiology* 2004;19(8):751-60. doi: 10.1023/B:EJEP.0000036568.02655.f8

For peer review only

54. Cutler RB, Fishbain DA, Cole B, et al. Identifying patients at risk for loss to follow-up after pain center treatment. *Pain Med* 2001;2(1):46-51. doi: 10.1046/j.1526-4637.2001.002001046.x [published Online First: 2004/04/23]

FIGURE LEGENDS

Figure 1 – Progression of health utility by chronic pain (CP) status 1-year after total knee replacement surgery, defined using a threshold score in the Oxford Knee Score pain subscale

Figure 2 – Yearly healthcare costs by chronic pain (CP) status 1-year after total knee replacement surgery, defined using a threshold score in the Oxford Knee Score pain subscale

Figure 3 – Progression of chronic pain status over five years after total knee replacement (TKR), showing participants in chronic pain (dark grey) and not in chronic pain (light grey). Chronic pain status set using a threshold value for the mean Oxford Knee Score pain subscale value across 50 imputations for each participant at each year.

For peer review only

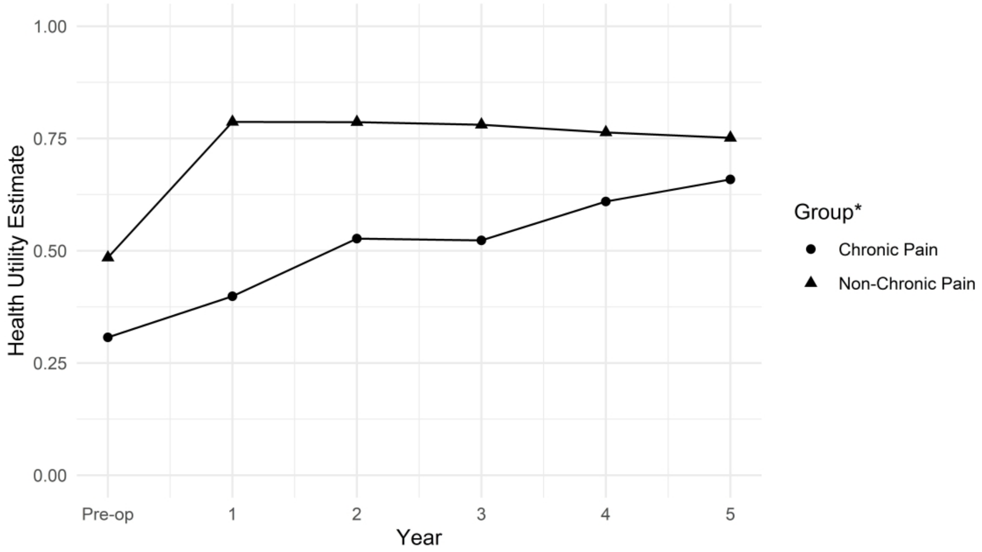

179x101mm (1200 x 1200 DPI)

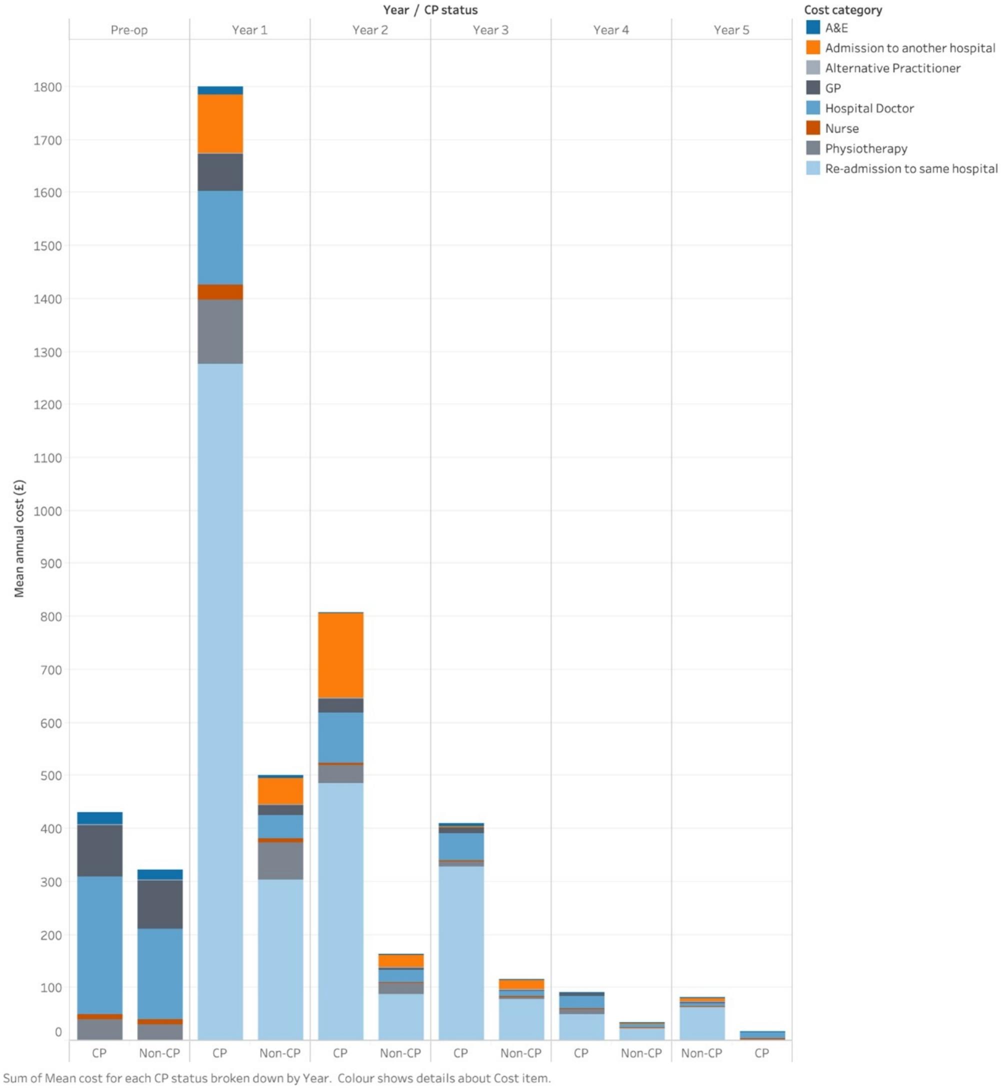

159x173mm (220 x 220 DPI)

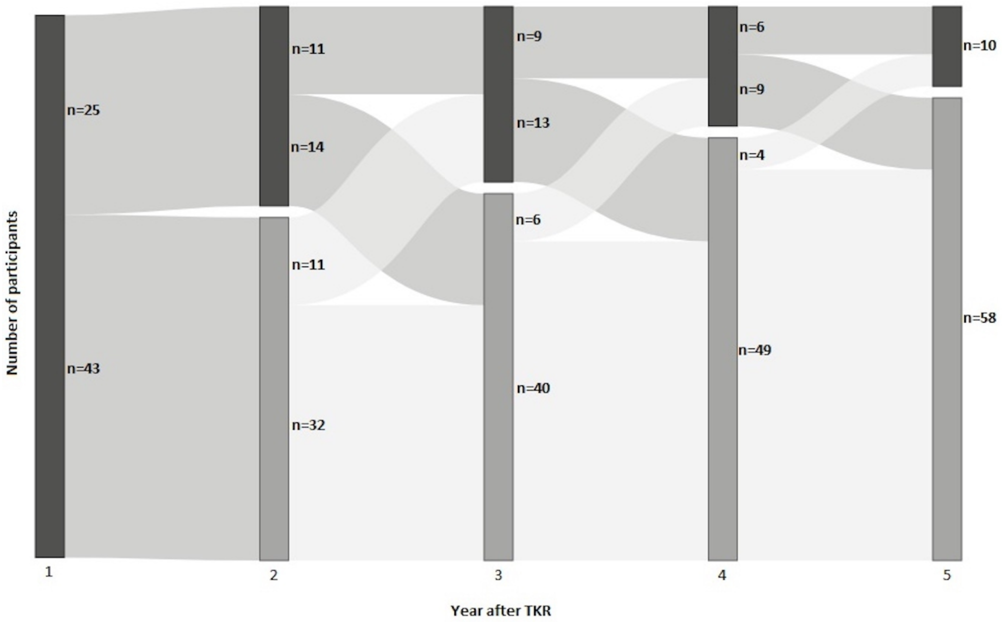

229x146mm (150 x 150 DPI)

ONLINE SUPPLEMENTAL MATERIAL

Tables

Table A.1: Observed and imputed health utility and Oxford Knee Score pain subscale (OKS-PS) scores per year

Observed						Imputed								
year	n	mean	SD	min	max	year	n	Pooled mean	Pooled SE	95% confidence interval		min	max
Health utility (EQ5D-3L) index													
pre-op	491	0.447	0.291	-0.239	1	pre-op	550	0.462	0.112	0.243	0.682	-0.349	1
1	531	0.742	0.253	-0.319	1	1	550	0.738	0.104	0.534	0.943	-0.319	1
2	447	0.765	0.265	-0.429	1	2	550	0.754	0.106	0.546	0.962	-0.429	1
3	403	0.761	0.253	-0.358	1	3	550	0.748	0.103	0.547	0.950	-0.358	1
4	344	0.759	0.263	-0.135	1	4	550	0.744	0.103	0.542	0.947	-0.239	1
5	299	0.751	0.272	-0.239	1	5	550	0.740	0.104	0.536	0.943	-0.239	1
OKS pain subscale score													
pre-op	488	10.09	4.59	0	26	pre-op	550	10.85	0.48	9.90	11.79	0	28
1	525	22.06	6.14	1	28	1	550	22.09	0.51	21.09	23.09	1	28
2	447	23.09	5.71	0	28	2	550	22.66	0.51	21.67	23.66	0	28
3	405	23.37	5.36	3	28	3	550	22.91	0.50	21.94	23.89	1	28
4	350	23.61	5.37	3	28	4	550	23.38	0.49	22.41	24.35	1	28
5	298	23.45	5.52	6	28	5	550	23.43	0.50	22.45	24.40	2	28

Pre-op: pre-operative; SD: standard deviation; SE: standard error

For peer review only - http://bmjopen.bmj.com/site/about/guidelines.xhtml

Table A.2: Year 5 study participant demographics

Variable	Observed OKS-PS at Year 5 (n=286)			Missing OKS-PS at Year 5 (n=264)		
**Age at total knee replacement (n, mean, mode)**						
	286	70	73	264	70	73
**Gender (n, %)**						
Female	159	55.6		155	58.7	
Male	127	44.4		109	41.3	
**IMD 2010 score (n, mean, mode)**						
	285	10.9	7.17	264	14.8	15.71
Missing (n (%))	1	(0.35)		0	(0.00)	
**BMI (n, mean, mode)**						
	284	30.3	26.79	262	31.0	30.9
Missing (n (%))	2	(0.70)		2	(0.76)	
**OKS at pre-operation (n, mean, (SD))**						
	251	20.1	(7.17)	233	18.4	(7.78)
Missing (n (%))	35	(12.24)		31	(11.74)	
**EQ5D at pre-operation (n, mean, (SD))**						
	252	0.4736	(0.276)	239	0.4180	(0.304)
Missing (n (%))	34	(11.89)		25	(9.47)	

For peer review only

Table A.3: Number of missing items during the 5 years of follow-up

	Total		Non-Chronic Pain		Chronic Pain		No OKS Category	
	n	%	n	%	n	%	n	%
**Number of years of missing OKS items**								
0	175	31.8	163	35.6	12	17.9	0	0
1	141	25.6	120	26.2	18	26.9	3	12
2	113	20.5	94	20.5	9	13.4	10	40
3	57	10.4	42	9.2	12	17.9	3	12
4	56	10.2	37	8.1	15	22.4	4	16
5	8	1.5	2	0.4	1	1.5	5	20
**Number of years of missing EQ5D items**								
0	219	39.8	201	43.9	14	20.9	4	16
1	116	21.1	95	20.7	14	20.9	7	28
2	100	18.2	81	17.7	14	20.9	5	20
3	56	10.2	41	9	13	19.4	2	8
4	53	9.6	37	8.1	11	16.4	5	20
5	6	1.1	3	0.7	1	1.5	2	8

For peer review only

Table A.4: Logistic regression p-values: preoperative variables associated with missingness - significant at a 5% level

	Missing OKS-PS				
	**Year 1**	**Year 2**	**Year 3**	**Year 4**	**Year 5**
**Age at operation**	0.227	0.790	0.906	0.483	0.537
**Gender**	0.058*	0.172	0.410	0.079*	0.475
**EQ5D dimension 1**	0.986	0.241	0.395	0.670	0.348
**EQ5D dimension 2**	**0.021**	**0.015**	**0.007**	**0.009**	**0.020**
**EQ5D dimension 3**	0.205	**0.010**	**0.037**	0.088*	0.211
**EQ5D dimension 4**	**0.025**	**0.008**	**0.025**	**0.002**	0.116
**EQ5D dimension 5**	0.474	**0.030**	0.133	0.404	0.101
**OKS Q1**	0.939	**0.026**	**0.024**	**0.016**	0.165
**OKS Q4**	0.052*	**0.000**	**0.003**	**0.000**	**0.003**
**OKS Q5**	0.361	0.138	0.173	**0.025**	0.633
**OKS Q6**	**0.039**	**0.016**	**0.021**	0.056*	0.997
**OKS Q8**	0.839	0.080*	0.424	**0.014**	0.641
**OKS Q9**	0.184	**0.004**	0.140	**0.001**	0.105
**OKS Q10**	0.086*	**0.000**	**0.000**	**0.001**	**0.006**

*significant at a 10% level only

For peer review only

Table A.5: Health utility scores per year by chronic pain group

	Pooled mean	Pooled SE	95% confidence interval	
**Chronic pain 1-year after total knee replacement**				
Pre-op	0.307	0.197	-0.079	0.694
Year 1	0.399	0.194	0.019	0.778
Year 2	0.527	0.193	0.148	0.906
Year 3	0.523	0.194	0.143	0.904
Year 4	0.610	0.186	0.245	0.974
Year 5	0.659	0.183	0.300	1.018
**Non-chronic pain 1-year after total knee replacement**				
Pre-op	0.485	0.114	0.262	0.708
Year 1	0.787	0.097	0.597	0.976
Year 2	0.786	0.105	0.581	0.991
Year 3	0.781	0.100	0.584	0.977
Year 4	0.764	0.104	0.559	0.968
Year 5	0.751	0.106	0.543	0.960

Pre-op: pre-operative; SE: standard error

For peer review only

Table A.6: Oxford Knee Score pain subscale (OKS-PS) scores per year by chronic pain group

	Pooled mean	Pooled SE	Confidence interval	
**Chronic pain 1-year after total knee replacement**				
Pre-op	8.04	0.79	6.50	9.59
Year 1	9.73	0.66	8.45	11.02
Year 2	16.06	0.98	14.15	17.98
Year 3	17.05	1.00	15.09	19.00
Year 4	19.17	1.01	17.19	21.15
Year 5	20.80	0.98	18.88	22.71
**Non-chronic pain 1-year after total knee replacement**				
Pre-op	11.25	0.50	10.27	12.22
Year 1	23.86	0.42	23.03	24.69
Year 2	23.61	0.49	22.65	24.56
Year 3	23.76	0.48	22.81	24.70
Year 4	23.98	0.48	23.03	24.93
Year 5	23.80	0.50	22.82	24.79

Pre-op: pre-operative; SE: standard error

For peer review only

For peer review only - http://bmjopen.bmj.com/site/about/guidelines.xhtml

Table A.7a: Primary care healthcare resource use by chronic pain (CP) group, defined 1-year after total knee replacement (Pre-op, Year 1 and Year 2)

	Pre-op		Year 1		Year 2	
	**CP (n=70)**	**Non-CP (n=482)**	**CP (n=70)**	**Non-CP (n=482)**	**CP (n=70)**	**Non-CP (n=482)**
**Saw a general practitioner (n (%))**						
Yes	58 (82.86)	390 (80.91)	44 (62.86)	135 (28.01)	24 (34.29)	46 (9.54)
No	8 (11.43)	43 (8.92)	23 (32.86)	335 (69.5)	22 (31.43)	361 (74.9)
Missing	*4 (5.71)*	*49 (10.17)*	*3 (4.29)*	*12 (2.49)*	*24 (34.29)*	*75 (15.56)*
Number of visits – last 12 months (mean (total))						
NHS	3.8 (209)	3.43 (1323)	2.66 (154)	0.64 (288)	1.29 (54)	0.21 (81)
Private	0 (0)	0.08 (4)	0.25 (6)	0.02 (6)	0 (0)	0 (0)
**Saw a physiotherapist**						
Yes	25 (35.71)	153 (31.74)	42 (60)	215 (44.61)	15 (21.43)	71 (14.73)
No	40 (57.14)	251 (52.07)	25 (35.71)	253 (52.49)	29 (41.43)	330 (68.46)
Missing	*5 (7.14)*	*78 (16.18)*	*3 (4.29)*	*14 (2.9)*	*26 (37.14)*	*81 (16.8)*
Number of visits – last 12 months (mean (total))						
NHS	1.35 (81)	1.12 (422)	4.08 (241)	2.28 (973)	1.65 (66)	0.81 (314)
Private	0.02 (1)	0.34 (92)	2.97 (86)	0.6 (167)	0 (0)	0.12 (39)
**Saw a nurse/practitioner**						
Yes	14 (20)	102 (21.16)	18 (25.71)	84 (17.43)	4 (5.71)	16 (3.32)
No	48 (68.57)	292 (60.58)	41 (58.57)	369 (76.56)	36 (51.43)	373 (77.39)
Missing	*8 (11.43)*	*88 (18.26)*	*11 (15.71)*	*29 (6.02)*	*30 (42.86)*	*93 (19.29)*
Number of visits – last 12 months (mean (total))						
NHS	0.7 (42)	0.72 (271)	2.34 (131)	0.47 (204)	0.43 (17)	0.08 (31)
Private	0 (0)	0 (0)	0 (0)	0 (0)	0 (0)	0 (1)
**Saw an alternative practitioner**						
Yes	3 (4.29)	41 (8.51)	7 (10)	19 (3.94)	4 (5.71)	5 (1.04)
No	61 (87.14)	386 (80.08)	62 (88.57)	456 (94.61)	42 (60)	406 (84.23)
Missing	*6 (8.57)*	*55 (11.41)*	*1 (1.43)*	*7 (1.45)*	*24 (34.29)*	*71 (14.73)*
Number of visits – last 12 months (mean (total))						
NHS	0.03 (2)	0.12 (46)	0 (0)	0.01 (3)	0.12 (5)	0 (0)
Private	0.13 (8)	0.32 (134)	0.32 (21)	0.11 (51)	0.2 (9)	0.11 (46)

For peer review only - http://bmjopen.bmj.com/site/about/guidelines.xhtml

Table A.7b: Primary care healthcare resource use by chronic pain (CP) group, defined 1-year after total knee replacement (Years 3-5)

	**Year 3**		**Year 4**		**Year 5**	
	**CP (n=70)**	**Non-CP (n=482)**	**CP (n=70)**	**Non-CP (n=482)**	**CP (n=70)**	**Non-CP (n=482)**
**Saw a general practitioner (n (%))**						
Yes	13 (18.57)	28 (5.81)	7 (10)	17 (3.53)	6 (8.57)	23 (4.77)
No	29 (41.43)	355 (73.65)	24 (34.29)	312 (64.73)	16 (22.86)	271 (56.22)
Missing	*28 (40)*	*99 (20.54)*	*39 (55.71)*	*153 (31.74)*	*48 (68.57)*	*188 (39)*
Number of visits – last 12 months (mean (total))						
NHS	0.69 (27)	0.16 (61)	0.63 (19)	0.11 (36)	0.33 (6)	0.13 (36)
Private	0 (0)	0 (0)	0 (0)	0 (0)	0.06 (1)	0 (0)
**Saw a physiotherapist**						
Yes	7 (10)	20 (4.15)	3 (4.29)	10 (2.07)	2 (2.86)	11 (2.28)
No	34 (48.57)	353 (73.24)	25 (35.71)	307 (63.69)	17 (24.29)	275 (57.05)
Missing	*29 (41.43)*	*109 (22.61)*	*42 (60)*	*165 (34.23)*	*51 (72.86)*	*196 (40.66)*
Number of visits – last 12 months (mean (total))						
NHS	29 (41.43)	355 (73.65)	24 (34.29)	312 (64.73)	16 (22.86)	271 (56.22)
Private	*28 (40)*	*99 (20.54)*	*39 (55.71)*	*153 (31.74)*	*48 (68.57)*	*188 (39)*
**Saw a nurse/practitioner**						
Yes	6 (8.57)	3 (0.62)	1 (1.43)	3 (0.62)	0 (0)	1 (0.21)
No	35 (50)	365 (75.73)	26 (37.14)	314 (65.15)	19 (27.14)	283 (58.71)
Missing	*29 (41.43)*	*114 (23.65)*	*43 (61.43)*	*165 (34.23)*	*51 (72.86)*	*198 (41.08)*
Number of visits – last 12 months (mean (total))						
NHS	0.16 (6)	0.02 (7)	0.15 (4)	0.02 (7)	0 (0)	0 (0)
Private	0 (0)	0 (0)	0 (0)	0 (0)	0 (0)	0 (0)
**Saw an alternative practitioner**						
Yes	6 (8.57)	6 (1.24)	2 (2.86)	7 (1.45)	1 (1.43)	3 (0.62)
No	37 (52.86)	374 (77.59)	28 (40)	322 (66.8)	20 (28.57)	290 (60.17)
Missing	*27 (38.57)*	*102 (21.16)*	*40 (57.14)*	*153 (31.74)*	*49 (70)*	*189 (39.21)*
Number of visits – last 12 months (mean (total))						
NHS	0.03 (1)	0.01 (5)	0 (0)	0.02 (6)	0 (0)	0 (0)
Private	0.48 (19)	0.11 (42)	0.07 (2)	0.05 (15)	0 (0)	0 (0)

For peer review only - http://bmjopen.bmj.com/site/about/guidelines.xhtml

Table A.8a: Hospital care resource use by chronic pain (CP) group, defined 1-year after total knee replacement (Pre-op, Year 1 and Year 2)

	Pre-op		Year 1		Year 2	
	**CP (n=70)**	**Non-CP (n=482)**	**CP (n=70)**	**Non-CP (n=482)**	**CP (n=70)**	**Non-CP (n=482)**
**Saw a hospital doctor**						
Yes	53 (75.71)	354 (73.44)	36 (51.43)	106 (21.99)	23 (32.86)	43 (8.92)
No	12 (17.14)	65 (13.49)	27 (38.57)	355 (73.65)	22 (31.43)	357 (74.07)
Missing	*5 (7.14)*	*63 (13.07)*	*7 (10)*	*21 (4.36)*	*25 (35.71)*	*82 (17.01)*
Number of visits – last 12 months (mean (total))						
NHS	2.52 (151)	1.8 (687)	1.72 (103)	0.4 (177)	1.3 (56)	0.22 (88)
Private	0.08 (1)	0.17 (13)	0 (0)	0 (1)	0 (0)	0 (0)
**Visited an accident and emergency department**						
Yes	9 (12.86)	51 (10.58)	6 (8.57)	15 (3.11)	1 (1.43)	5 (1.04)
No	55 (78.57)	373 (77.39)	62 (88.57)	462 (95.85)	44 (62.86)	403 (83.61)
Missing	*6 (8.57)*	*58 (12.03)*	*2 (2.86)*	*5 (1.04)*	*25 (35.71)*	*74 (15.35)*
Number of visits – last 12 months (mean (total))						
NHS	0.21 (13)	0.17 (72)	0.24 (16)	0.03 (12)	0.02 (1)	0.02 (7)
**Re-admitted to the (site-specific) hospital**						
Yes			15 (21.43)	29 (6.02)	6 (8.57)	8 (1.66)
No			54 (77.14)	447 (92.74)	40 (57.14)	409 (84.85)
Missing			*1 (1.43)*	*6 (1.24)*	*24 (34.29)*	*65 (13.49)*
Number of visits – last 12 months (mean (total))						
Days			1.07 (73)	0.25 (117)	0.59 (27)	0.04 (18)
**Admitted to any other hospital**						
Yes			1 (1.43)	6 (1.24)	2 (2.86)	2 (0.41)
No			67 (95.71)	470 (97.51)	45 (64.29)	412 (85.48)
Missing			*2 (2.86)*	*6 (1.24)*	*23 (32.86)*	*68 (14.11)*
Number of visits – last 12 months (mean (total))						
Days			0.1 (7)	0.04 (17)	0.43 (20)	0.01 (5)

For peer review only - http://bmjopen.bmj.com/site/about/guidelines.xhtml

Table A.8b: Hospital care resource use by chronic pain (CP) group, defined 1-year after total knee replacement (Years 3-5)

	Year 3		Year 4		Year 5	
	**CP (n=70)**	**Non-CP (n=482)**	**CP (n=70)**	**Non-CP (n=482)**	**CP (n=70)**	**Non-CP (n=482)**
**Saw a hospital doctor**						
Yes	14 (20)	19 (3.94)	7 (10)	10 (2.07)	6 (8.57)	16 (3.32)
No	31 (44.29)	355 (73.65)	24 (34.29)	311 (64.52)	16 (22.86)	274 (56.85)
Missing	*25 (35.71)*	*108 (22.41)*	*39 (55.71)*	*161 (33.4)*	*48 (68.57)*	*192 (39.83)*
Number of visits – last 12 months (mean (total))						
NHS	0.7 (30)	0.11 (41)	0.45 (13)	0.06 (20)	0.29 (6)	0.08 (23)
Private	0 (0)	0 (0)	0 (0)	0 (0)	0 (0)	0 (0)
**Visited an accident and emergency department**						
Yes	2 (2.86)	5 (1.04)	0 (0)	3 (0.62)	0 (0)	3 (0.62)
No	41 (58.57)	375 (77.8)	31 (44.29)	323 (67.01)	21 (30)	288 (59.75)
Missing	*27 (38.57)*	*102 (21.16)*	*39 (55.71)*	*156 (32.37)*	*49 (70)*	*191 (39.63)*
Number of visits – last 12 months (mean (total))						
NHS	0.09 (4)	0.01 (2)	0 (0)	0 (1)	0 (0)	0 (0)
**Re-admitted to the (site-specific) hospital**						
Yes	3 (4.29)	6 (1.24)	1 (1.43)	3 (0.62)	0 (0)	4 (0.83)
No	40 (57.14)	383 (79.46)	32 (45.71)	331 (68.67)	24 (34.29)	290 (60.17)
Missing	*27 (38.57)*	*93 (19.29)*	*37 (52.86)*	*148 (30.71)*	*46 (65.71)*	*188 (39)*
Number of visits – last 12 months (mean (total))						
Days	0.14 (6)	0.12 (46)	0.06 (2)	0.09 (31)	0 (0)	0.03 (8)
**Admitted to any other hospital**						
Yes	0 (0)	1 (0.21)	0 (0)	0 (0)	0 (0)	1 (0.21)
No	42 (60)	386 (80.08)	33 (47.14)	332 (68.88)	23 (32.86)	291 (60.37)
Missing	*28 (40)*	*95 (19.71)*	*37 (52.86)*	*150 (31.12)*	*47 (67.14)*	*190 (39.42)*
Number of visits – last 12 months (mean (total))						
Days	0 (0)	0 (1)	0 (0)	0 (0)	0 (0)	0 (1)

For peer review only - http://bmjopen.bmj.com/site/about/guidelines.xhtml

Table A.9a: Yearly healthcare costs by chronic pain (CP) (Pre-op, Year 1 and Year 2)

	CP at year 1 (n = 70)		Non-CP at year 1 (n = 482)	
	**Mean**	**(SD)**	**Mean**	**(SD)**
**Pre-operative - Healthcare professionals**				
General practitioner	98.53	(124.17)	90.58	(104.96)
Physiotherapist	40.50	(93.39)	30.64	(73.90)
Hospital doctor	258.86	(559.74)	171.04	(225.45)
Nurse	9.30	(27.60)	8.71	(29.74)
Alternative practitioner	1.00	(8.37)	3.34	(50.00)
**Pre-op - Hospital visits**				
Accident and emergency	21.60	(56.64)	17.78	(51.73)
Re-admitted to hospital				
Admitted to another hospital				
**Pre-op - Total**	**429.79**	**(611.41)**	**322.09**	**(337.22)**
**Year 1 - Healthcare professionals**				
General practitioner	72.60	(133.02)	19.72	(53.62)
Physiotherapist	120.50	(179.62)	70.65	(132.22)
Hospital doctor	176.57	(300.76)	44.07	(119.12)
Nurse	29.01	(118.95)	6.56	(24.31)
Alternative practitioner	0.00	(0.00)	0.22	(2.76)
**Year 1 - Hospital visits**				
Accident and emergency	14.40	(47.37)	5.23	(29.20)
Re-admitted to hospital	1276.42	(2647.66)	303.54	(1325.34)
Admitted to another hospital	109.47	(915.92)	51.14	(489.30)
**Year 1 - Total**	**1,798.97**	**(2981.69)**	**501.12**	**(1511.82)**
**Year 2 - Healthcare professionals**				
General practitioner	25.46	(48.06)	5.55	(24.42)
Physiotherapist	33.00	(95.13)	22.80	(73.23)
Hospital doctor	96.00	(220.61)	21.91	(109.69)
Nurse	3.76	(20.00)	1.00	(7.58)
Alternative practitioner	2.50	(20.92)	0.00	(0.00)
**Year 2 - Hospital visits**				
Accident and emergency	2.40	(20.08)	1.74	(17.04)
Re-admitted to hospital	486.42	(1825.23)	86.54	(791.78)
Admitted to another hospital	158.00	(996.48)	22.95	(381.51)
**Year 2 - Total**	**807.54**	**(2233.38)**	**162.48**	**(970.07)**

For peer review only

For peer review only - http://bmjopen.bmj.com/site/about/guidelines.xhtml

Table A.9b: Yearly healthcare costs by chronic pain (CP) (Years 3-5)

	CP at year 1 (n = 70)		Non-CP at year 1 (n = 482)	
	Mean	(SD)	Mean	(SD)
**Year 3 - Healthcare professionals**				
General practitioner	12.73	(36.97)	4.18	(24.51)
Physiotherapist	9.50	(46.89)	4.72	(28.11)
Hospital doctor	51.43	(136.40)	10.21	(64.63)
Nurse	1.33	(6.32)	0.23	(4.29)
Alternative practitioner	0.50	(4.18)	0.36	(7.97)
**Year 3 - Hospital visits**				
Accident and emergency	4.80	(28.19)	1.74	(17.04)
Re-admitted to hospital	328.42	(1563.26)	77.69	(728.20)
Admitted to another hospital	0.00	(0.00)	15.90	(349.05)
**Year 3 - Total**	**408.71**	**(1630.76)**	**115.02**	**(865.95)**
**Year 4 - Healthcare professionals**				
General practitioner	8.96	(33.20)	2.46	(21.45)
Physiotherapist	11.00	(57.94)	2.03	(22.70)
Hospital doctor	22.29	(89.74)	4.98	(42.79)
Nurse	0.89	(7.41)	0.23	(3.53)
Alternative practitioner	0.00	(0.00)	0.44	(8.13)
**Year 4 - Hospital visits**				
Accident and emergency	0.00	(0.00)	1.05	(13.23)
Re-admitted to hospital	48.53	(406.00)	22.95	(381.51)
Admitted to another hospital	0.00	(0.00)	0.00	(0.00)
**Year 4 - Total**	**91.66**	**(471.78)**	**34.13**	**(431.45)**
**Year 5 - Healthcare professionals**				
General practitioner	2.83	(17.54)	2.46	(17.63)
Physiotherapist	4.50	(27.90)	1.38	(14.12)
Hospital doctor	10.29	(39.53)	5.73	(43.74)
Nurse	0.00	(0.00)	0.00	(0.00)
Alternative practitioner	0.00	(0.00)	0.00	(0.00)
**Year 5 - Hospital visits**				
Accident and emergency	0.00	(0.00)	1.05	(13.23)
Re-admitted to hospital	0.00	(0.00)	63.59	(695.91)
Admitted to another hospital	0.00	(0.00)	7.05	(154.72)
**Year 5 - Total**	**17.61**	**(65.72)**	**81.26**	**(754.21)**

For peer review only

For peer review only - http://bmjopen.bmj.com/site/about/guidelines.xhtml

Table A.10: Mean costs for fluctuating and stable groups over chronic pain (CP) status between 1 and 2 years after total knee replacement – Year 1 CP

	Year 1 CP to Year 2 CP (n=25)				Year 1 CP to Year 2 Non-CP (n=19)			
	Year 1		Year 2		Year 1		Year 2	
	mean	SD	mean	SD	mean	SD	mean	SD
**Staff costs**								
General practitioner	52.8	82.50	42.2	49.06	78.2	188.36	33.0	65.08
Physiotherapist	96.6	159.84	60.2	135.37	71.8	89.97	38.7	88.00
Hospital doctor	201.6	399.08	158.4	253.90	113.7	176.52	138.9	283.59
Nurse	47.7	186.69	3.7	12.88	23.7	72.77	9.0	35.54
Alternative practitioner	0.0	0.00	0.0	0.00	0.0	0.00	9.2	40.15
**Hospital visit costs**								
Accident and emergency	13.4	46.52	0.0	0.00	8.8	38.54	8.8	38.54
Re-admitted to hospital	884.8	2244.93	748.9	2188.64	1210.0	2870.87	806.6	2416.20
Admitted to another hospital	306.5	1532.63	442.4	1650.37	0.0	0.00	0.0	0.00
**Total costs**	1603.5	2891.87	1455.9	2710.89	1506.2	3100.76	1044.3	2785.68

For peer review only - http://bmjopen.bmj.com/site/about/guidelines.xhtml

Table A.11: Mean costs for fluctuating and stable groups over chronic pain (CP) status between 1 and 2 years after total knee replacement – Year 1 Non-CP

	Year 1 Non-CP to Year 2 Non-CP (n=382)				Year 1 Non-CP to Year 2 CP (n=22)			
	Year 1		Year 2		Year 1		Year 2	
	mean	SD	mean	SD	mean	SD	mean	SD
**Staff costs**								
General practitioner	20.0	57.16	5.5	23.99	43.5	54.26	24.0	52.11
Physiotherapist	66.5	124.34	24.3	75.25	101.8	164.49	63.6	120.10
Hospital doctor	41.8	116.11	20.4	100.63	125.5	223.66	125.5	280.76
Nurse	6.2	25.23	0.9	7.52	8.5	18.97	2.8	10.30
Alternative practitioner	0.3	3.09	0.0	0.00	0.0	0.00	0.0	0.00
**Hospital visit costs**								
Accident and emergency	3.5	24.09	1.8	17.12	15.3	49.43	7.6	35.82
Re-admitted to hospital	307.3	1345.38	60.2	677.32	657.1	1855.51	851.1	2320.22
Admitted to another hospital	35.6	346.23	20.1	392.08	348.3	1633.78	154.4	724.22
**Total costs**	481.2	1488.25	133.2	835.38	1300.0	2512.02	1229.0	2755.09

For peer review only

Figure legends

Figure A. 1 Multiple imputation boxplot based on the mean health utility score across all 50 imputations for each participant

Figure A. 2: Multiple imputation histogram based on the mean Oxford Knee Score pain subscale (OKS-PS) across all 50 imputations for each participant

Figure A.3 Number of years of OKS-PS missing items by CP status

Figure A.4 Number of years of EQ-5D missing items by CP status

Figure A. 5: Oxford Knee Score pain subscale (OKS-PS) mean scores over the 5 years after total knee replacement surgery for those in chronic pain and those not in chronic pain 1-year after surgery (based on an OKS-PS threshold score).

For peer review only

For peer review only - http://bmjopen.bmj.com/site/about/guidelines.xhtml

Figures

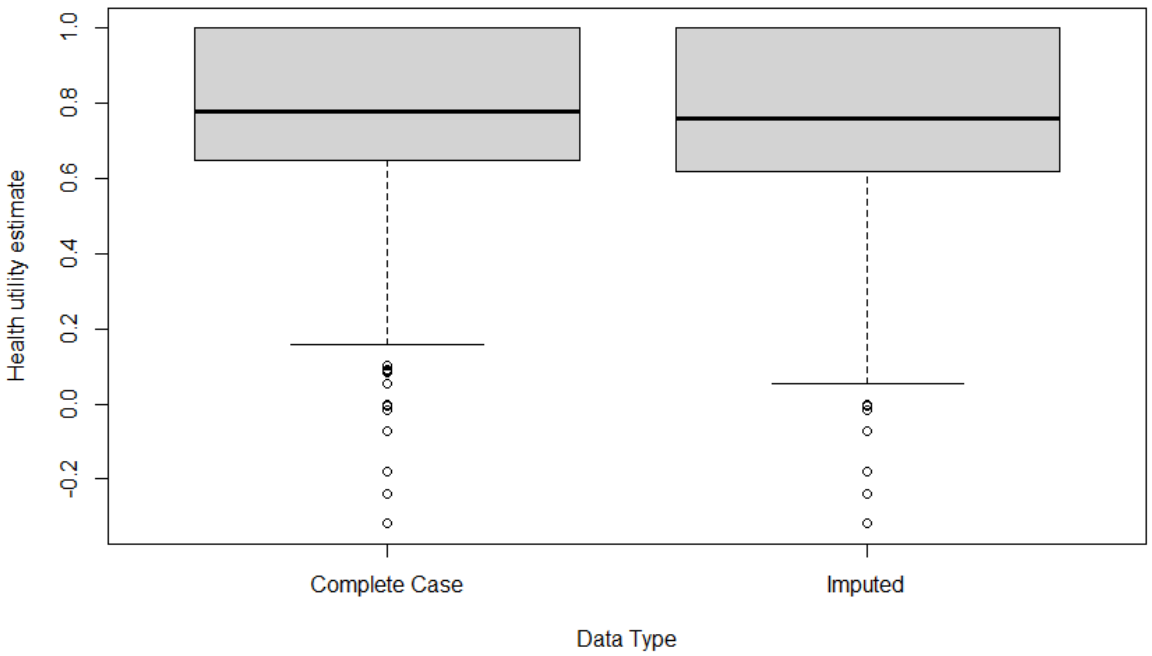

review only

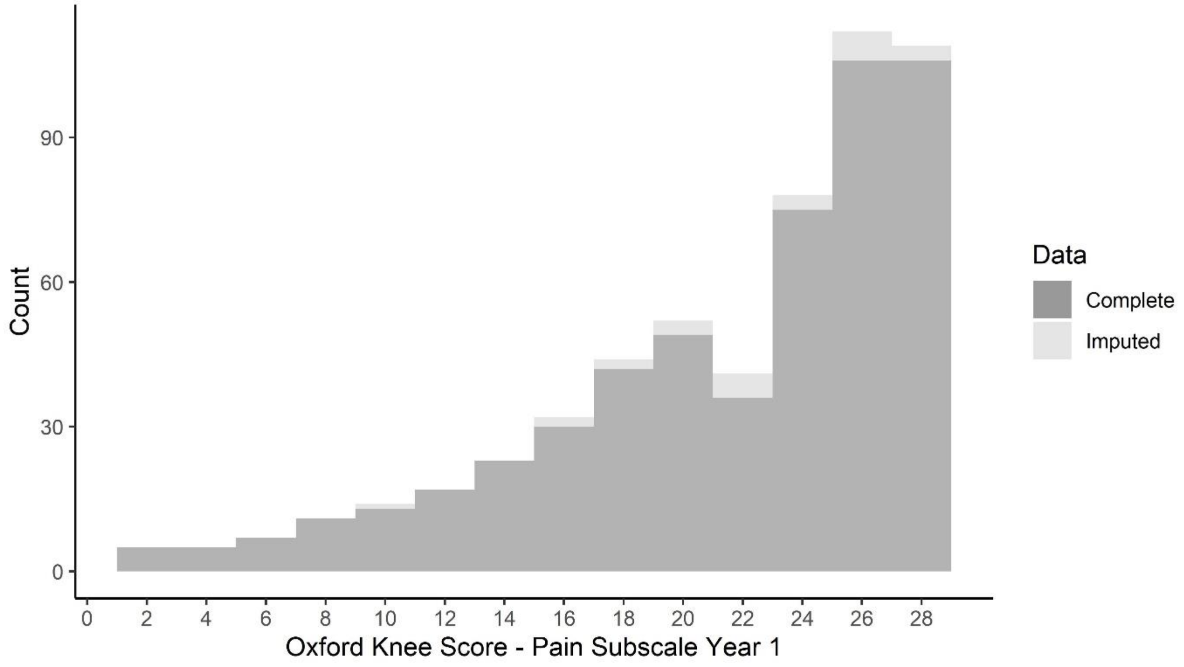

For peer review only - http://bmjopen.bmj.com/site/about/guidelines.xhtml

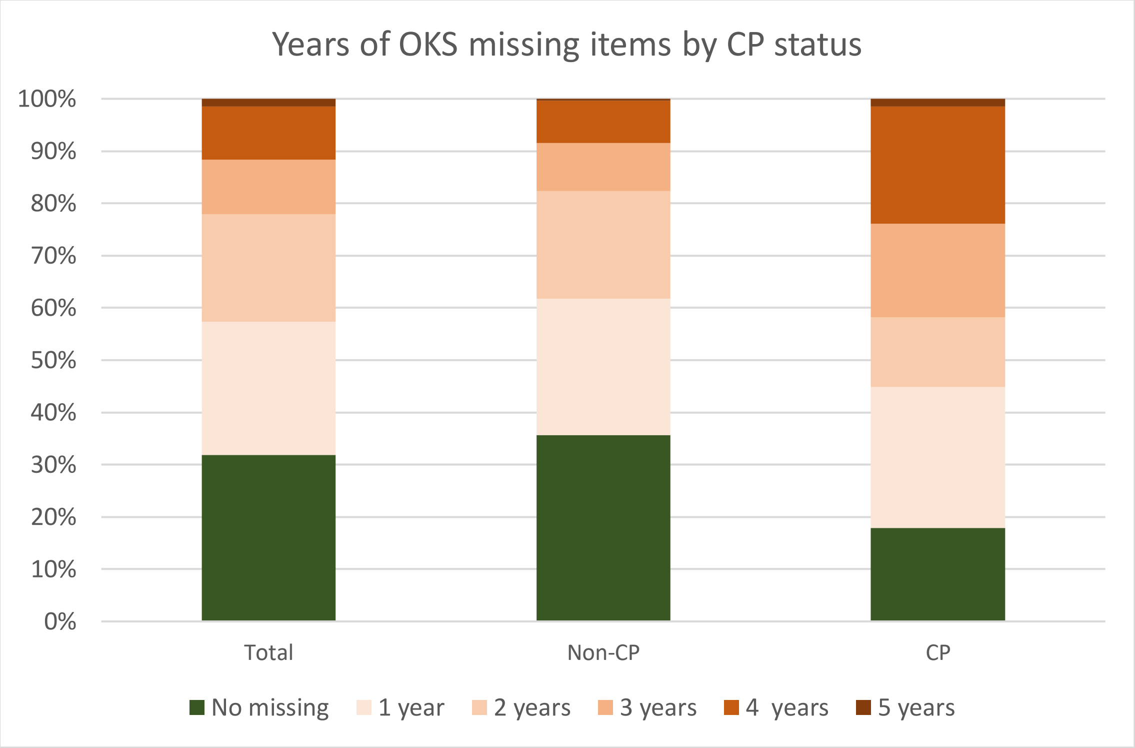

For peer review only - http://bmjopen.bmj.com/site/about/guidelines.xhtml

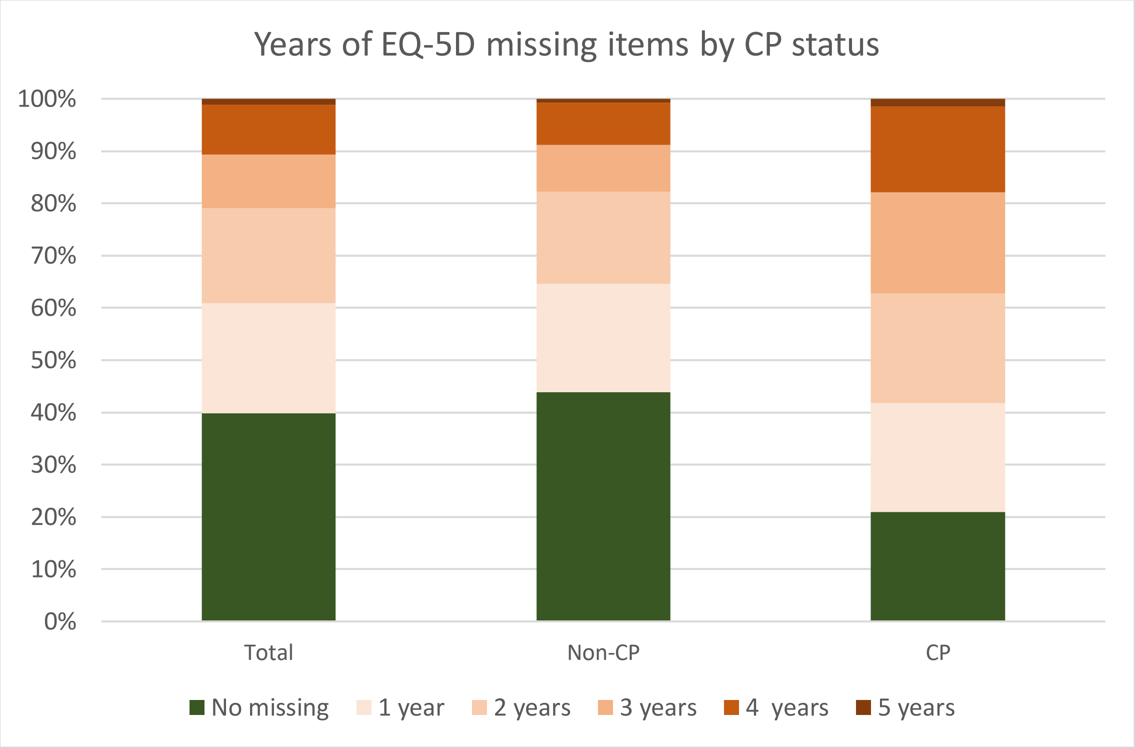

For peer review only - http://bmjopen.bmj.com/site/about/guidelines.xhtml

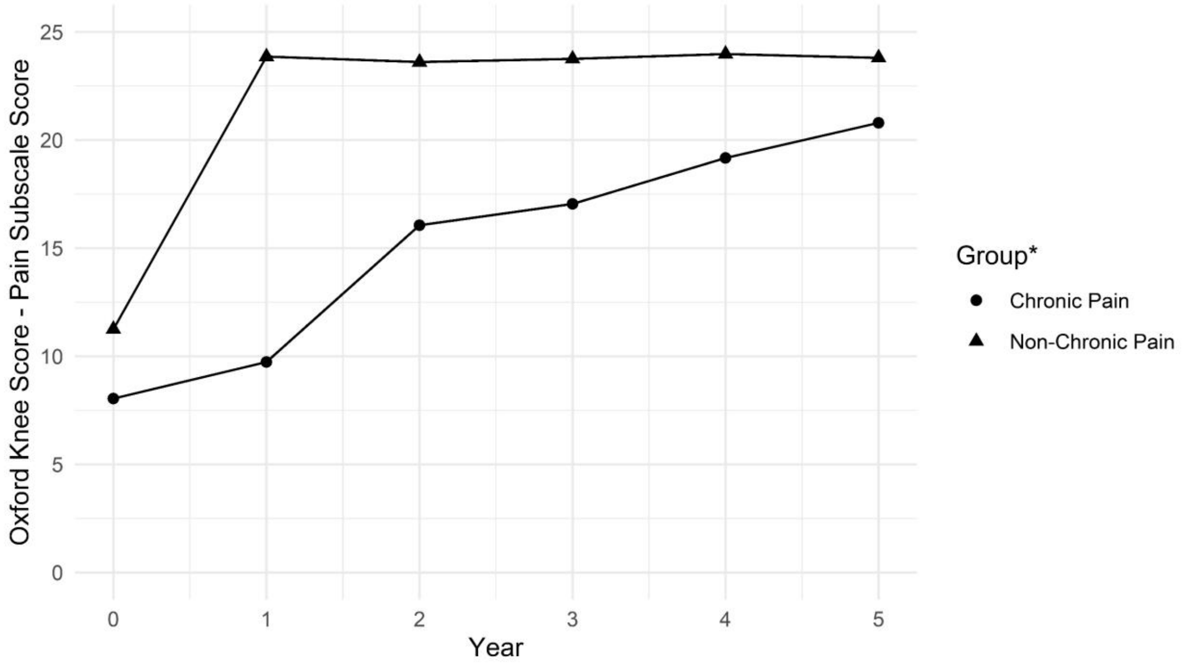

For peer review only

STROBE Statement—Checklist of items that should be included in reports of ***cohort studies***

	Item No	Recommendation	Page No
**Title and abstract**	1	(*a*) Indicate the study's design with a commonly used term in the title or the abstract<br>(*b*) Provide in the abstract an informative and balanced summary of what was done and what was found	1/3
**Introduction**			
Background/rationale	2	Explain the scientific background and rationale for the investigation being reported	4
Objectives	3	State specific objectives, including any prespecified hypotheses	5
**Methods**			
Study design	4	Present key elements of study design early in the paper	5
Setting	5	Describe the setting, locations, and relevant dates, including periods of recruitment, exposure, follow-up, and data collection	5
Participants	6	(*a*) Give the eligibility criteria, and the sources and methods of selection of participants. Describe methods of follow-up<br>(*b*) For matched studies, give matching criteria and number of exposed and unexposed	6
Variables	7	Clearly define all outcomes, exposures, predictors, potential confounders, and effect modifiers. Give diagnostic criteria, if applicable	7-8
Data sources/ measurement	8*	For each variable of interest, give sources of data and details of methods of assessment (measurement). Describe comparability of assessment methods if there is more than one group	5-8
Bias	9	Describe any efforts to address potential sources of bias	8
Study size	10	Explain how the study size was arrived at	6
Quantitative variables	11	Explain how quantitative variables were handled in the analyses. If applicable, describe which groupings were chosen and why	6-9
Statistical methods	12	(*a*) Describe all statistical methods, including those used to control for confounding<br>(*b*) Describe any methods used to examine subgroups and interactions<br>(*c*) Explain how missing data were addressed<br>(*d*) If applicable, explain how loss to follow-up was addressed<br>(*e*) Describe any sensitivity analyses	7-9
**Results**			
Participants	13*	(a) Report numbers of individuals at each stage of study—eg numbers potentially eligible, examined for eligibility, confirmed eligible, included in the study, completing follow-up, and analysed<br>(b) Give reasons for non-participation at each stage<br>(c) Consider use of a flow diagram	9-10
Descriptive data	14*	(a) Give characteristics of study participants (eg demographic, clinical, social) and information on exposures and potential confounders<br>(b) Indicate number of participants with missing data for each variable of interest<br>(c) Summarise follow-up time (eg, average and total amount)	10-12
Outcome data	15*	Report numbers of outcome events or summary measures over time	11-12

For peer review only

Main results	16	(*a*) Give unadjusted estimates and, if applicable, confounder-adjusted estimates and their precision (eg, 95% confidence interval). Make clear which confounders were adjusted for and why they were included<br>(*b*) Report category boundaries when continuous variables were categorized<br>(*c*) If relevant, consider translating estimates of relative risk into absolute risk for a meaningful time period	13-17
Other analyses	17	Report other analyses done—eg analyses of subgroups and interactions, and sensitivity analyses	
**Discussion**			
Key results	18	Summarise key results with reference to study objectives	17-19
Limitations	19	Discuss limitations of the study, taking into account sources of potential bias or imprecision. Discuss both direction and magnitude of any potential bias	19-21
Interpretation	20	Give a cautious overall interpretation of results considering objectives, limitations, multiplicity of analyses, results from similar studies, and other relevant evidence	22
Generalisability	21	Discuss the generalisability (external validity) of the study results	19-20
**Other information**			
Funding	22	Give the source of funding and the role of the funders for the present study and, if applicable, for the original study on which the present article is based	23-24

*Give information separately for exposed and unexposed groups.

Note: An Explanation and Elaboration article discusses each checklist item and gives methodological background and published examples of transparent reporting. The STROBE checklist is best used in conjunction with this article (freely available on the Web sites of PLoS Medicine at http://www.plosmedicine.org/, Annals of Internal Medicine at http://www.annals.org/, and Epidemiology at http://www.epidem.com/). Information on the STROBE Initiative is available at http://www.strobe-statement.org.

For peer review only
